# Prey-Impatience-Driven Sand Cat Swarm Optimization with Perturbation Learning for Global Optimization and Engineering Applications

**DOI:** 10.3390/biomimetics11080567

**Published:** 2026-08-08

**Authors:** Jiawen Wang, Jiayue Cai, Xuefei Xie, Yang Shen, Fanxing Meng, Yanxiu Yu, Dongman Cao

**Affiliations:** 1 School of Electronic Engineering and Automation, Dalian University of Engineering, Dalian 116102, China; wang1136@dlutci.edu.cn (J.W.); yu0777@dlutci.edu.cn (Y.Y.); 2 Zhan Tianyou College, Dalian Jiaotong University, Dalian 116102, China; caijaiyueovo@163.com (J.C.); 15703894726@163.com (X.X.); m18834052126@163.com (Y.S.); 3 Faculty of Electrical and Control Engineering, Liaoning Technical University, Fuxin 125105, China; 47221024@stu.lntu.edu.cn

**Keywords:** sand cat swarm optimization, good point set, opposition-based learning, random reuse, impatience coefficient, benchmark test functions, engineering design problems

## Abstract

Sand Cat Swarm Optimization (SCSO) is a swarm intelligence algorithm characterized by a simple structure and a small number of control parameters. However, when solving complex optimization problems, SCSO suffers from several limitations, including an uneven initial population distribution, excessive dependence on the current best individual during the search process, insufficient local exploitation accuracy, and susceptibility to local optima. To address these limitations, a Collaborative Multi-Strategy Sand Cat Swarm Optimization algorithm (CMSCSO) is proposed. The good point set method is adopted to generate a uniformly distributed initial population. An adaptive random reuse strategy is designed to selectively inherit dimensional information from the best individual according to differences in individual fitness. A prey impatience coefficient is introduced to dynamically adjust the local search intensity according to the distance between the population and the current best solution. In addition, a refractive-mechanism-based opposition-based learning strategy for the worst individuals is incorporated to update low-quality individuals and improve the ability of the algorithm to escape from local optima. CMSCSO was evaluated using the 30-dimensional CEC2017 and 10-dimensional CEC2022 benchmark suites. Its performance was compared with that of SCSO and several recently developed metaheuristic algorithms. The experimental results show that CMSCSO achieved the best mean values on 24 of the 29 CEC2017 benchmark functions and on 10 of the 12 CEC2022 benchmark functions. In the Wilcoxon tests conducted on CEC2017 and CEC2022, CMSCSO achieved 220 and 90 statistically significant wins, respectively. It also ranked first in the Friedman tests for both benchmark suites. For engineering optimization problems, the results obtained from six types of engineering design problems demonstrate that CMSCSO can consistently obtain high-quality feasible solutions that satisfy the specified constraints. In two-dimensional and three-dimensional wireless sensor network coverage optimization problems, coverage rates of 96.30% and 89.54% were achieved. For photovoltaic model parameter identification, CMSCSO achieved the highest identification accuracy. The numerical and engineering test results demonstrate that CMSCSO provides high optimization accuracy, strong stability, and good adaptability to complex engineering problems. It can therefore serve as an effective solution method for optimization tasks in structural design, mechanical engineering, and other related fields.

## 1. Introduction

Optimization is essentially a systematic process for solving extremum problems under complex constraints through mathematical modeling and deduction. It has been widely applied in parameter identification [1], production optimization [2], structural design [3], and other fields. As optimization problems become increasingly complex and large-scale, a large number of nonlinear factors and multiple constraints are introduced. As a result, traditional numerical methods can no longer meet the requirements for solving such optimization problems [4,5,6].

Metaheuristic optimization algorithms, inspired by natural phenomena or biological populations, have broken through the framework of traditional numerical methods. They do not rely on specific problems or initial values. Instead, they obtain the global optimum through random search and local exploitation within the boundary range. Therefore, they can be used to optimize problems in different fields. According to their underlying mechanisms, metaheuristic optimization algorithms can be divided into evolutionary algorithms, physics-based methods, and swarm intelligence algorithms. Evolutionary algorithms are based on Darwinian evolutionary theory. They use the concept of survival of the fittest to guide individual recombination or mutation, thereby enabling the population to evolve toward the optimal direction [7]. Typical examples of this type of algorithm include the Genetic Algorithm (GA) [8], Differential Evolution (DE) [9], and the Imperialist Competitive Algorithm (ICA) [10]. Through encoding mechanisms, evolutionary algorithms have been widely applied to scheduling, path planning, and related problems. However, their convergence accuracy is greatly affected by the encoding scheme and parameter settings [11,12]. Physics-based methods design algorithmic frameworks according to physical laws or physical phenomena in nature. They achieve stochastic search for the optimal solution. Representative algorithms include the Gravitational Search Algorithm (GSA) [13], Black Hole Algorithm (BHO) [14], and Artificial Electric Field Algorithm (AEFA) [15]. These methods have clear principles and require few hyperparameters. However, they often lack effective strategies for maintaining population diversity. This may lead to premature convergence. Swarm intelligence algorithms are inspired by the social behaviors of biological groups in nature. Compared with evolutionary algorithms and physics-based methods, swarm intelligence algorithms have the advantages of simple principles, easy implementation, and strong optimization capability [16].

In 1989, Beni first proposed the concept of swarm intelligence, which marked the beginning of swarm intelligence algorithms [17]. In 2014, Mirjalili et al. proposed the Grey Wolf Optimizer (GWO) for the first time. GWO simulates the strictly hierarchical social system and encircling hunting strategy of grey wolves. This algorithm possesses a compact parameter structure and a good balance between exploration and exploitation. Therefore, it has been widely applied to various engineering problems [18]. In 2019, Heidari et al. proposed the Harris Hawks Optimization (HHO) algorithm. HHO simulates the unique predation behavior of Harris hawks. The automatic transition between exploration and exploitation is achieved through the escape energy E. This algorithm has the advantages of high accuracy and strong robustness. However, it also suffers from excessive parameters and slow convergence [19]. In 2020, Xue et al. proposed the Sparrow Search Algorithm (SSA). This algorithm is inspired by the division of labor between foraging and anti-predation behaviors in sparrows. It has rapid convergence and strong local escape capability. However, its population diversity decreases significantly in the later stage. This makes the algorithm prone to premature convergence [20]. In recent years, swarm intelligence algorithms based on population behaviors have become one of the current research hotspots. Researchers have proposed many classic and efficient algorithms, such as the Beaver Behavior Optimizer (BBO) [21], Crocodile Ambush Optimization Algorithm (CAOA) [22], Fungal Growth Optimizer (FGO) [23], Mantis Shrimp Optimization Algorithm (MShOA) [24], Tetragonula Carbonaria Optimization Algorithm (TGCOA) [25], Tornado Optimizer with Coriolis Force (TOC) [26], and Zahhak Optimization Algorithm (ZOA) [27].

The Sand Cat Swarm Optimization algorithm was first proposed by Seyyedabbasi et al. in 2022 [28]. Owing to its concise algorithmic structure, SCSO can achieve global optimization using only two simple position updating formulas. SCSO simulates the auditory system of sand cats and realizes an adaptive balance between exploration and exploitation through the variation of the parameter *R*. Due to its excellent performance and low computational cost, SCSO has been widely applied to path planning [29], image segmentation [30], integrated energy system scheduling [31], and industrial prediction [32,33]. However, with the growing number of variables and constraints of real-world engineering problems, the limitations of SCSO have become more evident, as is the case with other metaheuristic algorithms.

(1) In the initialization phase, the population distribution is uneven, which reduces population diversity.

(2) In the prey search phase, namely the exploration phase, SCSO does not make full use of information from other individuals. Once the current best solution is unreliable, the whole population may deviate from the correct search direction.

(3) In the prey attack phase, namely the exploitation phase, SCSO relies only on the best individual. As the iterations proceed, the algorithm can easily fall into a local optimum and fail to find a better position.

(4) SCSO has insufficient ability to escape from local optima. It does not contain a dedicated mechanism for escaping local optima. Instead, it only relies on random numbers and random angles to maintain population diversity. As the iterations proceed, this escape ability decreases significantly.

To overcome the above limitations, Reference [34] incorporated chaotic maps to improve the quality of the initial population distribution and optimize the transition between exploration and exploitation, thereby significantly improving solution accuracy. Reference [35] modified the original SCSO using the golden sine strategy to achieve rapid convergence and avoid local optima. Reference [36] proposed an adaptive decay function to modify the position update formula of the population in the exploitation phase, which improved the global search capability of the algorithm. This method was further combined with GraphSAGE-GRU to substantially enhance prediction performance. Reference [37] integrated the Genetic Algorithm (GA) into the exploitation phase of SCSO to improve population diversity and evaluated its performance in a data classification task. Although the above methods have improved the performance of SCSO, most existing studies only address a single limitation and do not provide a comprehensive solution from an overall perspective. In addition, although the integration of other intelligent optimization algorithms can markedly improve the performance of SCSO, it also disrupts the original framework of SCSO. Therefore, how to comprehensively and collaboratively improve SCSO remains an important research topic.

To address these issues, this paper proposes a Collaborative Multi-Strategy Sand Cat Swarm Optimization (CMSCSO) algorithm to realize coordinated improvements in different phases of SCSO. The specific improvements are shown in Table 1.

Compared with existing improved SCSO methods, the collaborative search strategy proposed in this paper is not a simple superposition of multiple optimization operators. Instead, it is specifically designed according to the limitations of the original SCSO in different phases. In the initialization phase, the good point set strategy is adopted to improve population uniformity. In the exploration and exploitation phases, a random reuse strategy is introduced to reduce blind search. Meanwhile, a prey impatience coefficient is proposed to dynamically adjust the local search step size according to the distance between the predator and the prey, thereby improving local search accuracy. In the population update phase, worst-value opposition-based learning is used to improve the quality of inferior individuals. Each strategy corresponds to a different weakness in the search process of SCSO. Thus, the proposed method achieves a coordinated improvement in global search, local exploitation, and the ability to escape from local optima. Therefore, the innovation of this method does not lie in simply increasing the number of strategies. Instead, it lies in constructing a problem-oriented and multi-stage collaborative improvement framework based on the search mechanism of SCSO.

To comprehensively evaluate the performance of the algorithm, CMSCSO is compared with other recent metaheuristic algorithms. Statistical experiments are conducted on CMSCSO using the CEC2017 and CEC2022 benchmark test suites. On this basis, six engineering optimization problems, two WSN coverage optimization problems and photovoltaic model parameter identification problems are used to further verify the practicality of CMSCSO.

The main contributions of this paper are summarized as follows:

1. A multi-strategy collaborative search scheme is proposed. Targeted improvement strategies are designed for three key stages, namely initialization, exploration and exploitation, and escaping from local optima.

2. Extensive experiments are conducted on the CEC2017 and CEC2022 benchmark test suites to verify the effectiveness of the proposed CMSCSO.

3. Six engineering optimization problems, two WSN coverage optimization problems and photovoltaic model parameter identification problems are used to further validate the robustness and practicality of CMSCSO.

## 2. Standard SCSO and Proposed Method

### 2.1. Standard SCSO

The sand cat swarm optimization algorithm was first proposed by Seyyedabbasi [28]. Inspired by the auditory ability of sand cats, this algorithm simulates their hunting and predation behaviors. Corresponding mathematical models are established to solve optimization problems.

During initialization, SCSO generates the initial positions with a pseudo-random manner, as in other intelligent optimization algorithms:

(1) $$x_{i,j}=lb_{j}+r_{1}\cdot \left(ub_{j}-lb_{j}\right)$$

where *x_i_*_,_*_j_* denotes the *j*-th dimensional variable of the *i*-th sand cat, *lb_j_* and *ub_j_* represent the lower and upper bounds of the *j*-th dimensional variable, respectively, and *r*_1_ is a random number in [0, 1].

Sand cats rely on their acute auditory ability to hunt and capture prey. The auditory sensitivity *r_s_* is controlled as follows:

(2) $$r_{s}=2-2\frac{t}{T}$$

where *t* denotes the current iteration number and *T* denotes the maximum number of iterations. In a sand cat swarm, each sand cat has a different auditory ability. Therefore, the actual auditory sensitivity of each sand cat is expressed as follows:

(3) $$r=r_{s}\cdot r_{2}$$

where *r*_2_ is a random number in [0, 1]. The exploration and exploitation behaviors of SCSO are controlled by the transition coefficient *R*:

(4) $$R=2r_{s}\cdot r_{3}-r_{s}$$

where *r*_3_ is a random number in [0, 1]. As shown in (4), *R* decreases randomly within the interval [−2, 2]. When |*R*| > 1, SCSO performs exploration to enhance its global search capability. The position is updated as follows:

(5) $$X(t+1)=r(X_{best}(t)-r_{4}\cdot X(t))$$

where *X*(*t*) denotes the current position of the sand cat, *X_best_*(*t*) denotes the current best candidate position of the sand cat, and *r*_4_ is a random number in [0, 1].

When |R| ≤ 1, SCSO performs exploitation to refine the optimal solution:

(6) $$X_{r}(t)=\left|r_{5}\cdot X_{best}(t)-X(t)\right|$$

(7) $$X(t+1)=X_{best}(t)-rX_{r}(t)\cos\theta$$

where *θ* denotes the moving direction of the sand cat. Its value is selected from [0°, 360°] using the roulette wheel mechanism.

### 2.2. CMSCSO

Although SCSO has good performance, it still has certain limitations when dealing with high-dimensional, multimodal, and highly nonlinear problems. Therefore, further improvement is necessary. In this paper, SCSO is improved according to the framework shown in Table 1.

#### 2.2.1. Good Point Set Strategy

SCSO randomly generates the initial population using (1). The generated population is directly affected by the random number *r*_1_, which may lead to an uneven distribution in the solution space. This reduces the utilization efficiency of the entire solution space by SCSO. To address this problem, the good point set strategy is adopted to initialize the population distribution. The good point set strategy was first proposed by Hua Luogeng. In an *s*-dimensional Euclidean space, there exists a good point set *P_n_*(*k*):

(8) $$P_{n}(k)=\left\{\left(\left\{r_{1}^{\left(n\right)}\cdot k\right\},\left\{r_{2}^{\left(n\right)}\cdot k\right\},\cdots \left\{r_{s}^{\left(n\right)}\cdot k\right\}\right),1\leq k\leq n\right\}$$

where *k* denotes the sample index and *r_i_*^(^*^n^*^)^ denotes the base vector of the *n*-order *s*-dimensional good point. The notation { } represents the operation of taking the fractional part. The value of the good point set *r_i_* is defined as follows:

(9) $$r_{i}=2\cos\frac{2\pi i}{p},1\leq i\leq s$$

where *p* is the smallest prime number satisfying (*p* − 3)/2 ≥ *s*. Based on the good point set theory, the population initialization *xp_i_*_,_*_j_* of SCSO is expressed as follows:

(10) $$xp_{i,j}=lb_{j}+\left\{P_{n}(k)\right\}\cdot \left(ub_{j}-lb_{j}\right)$$

**Figure 1:**
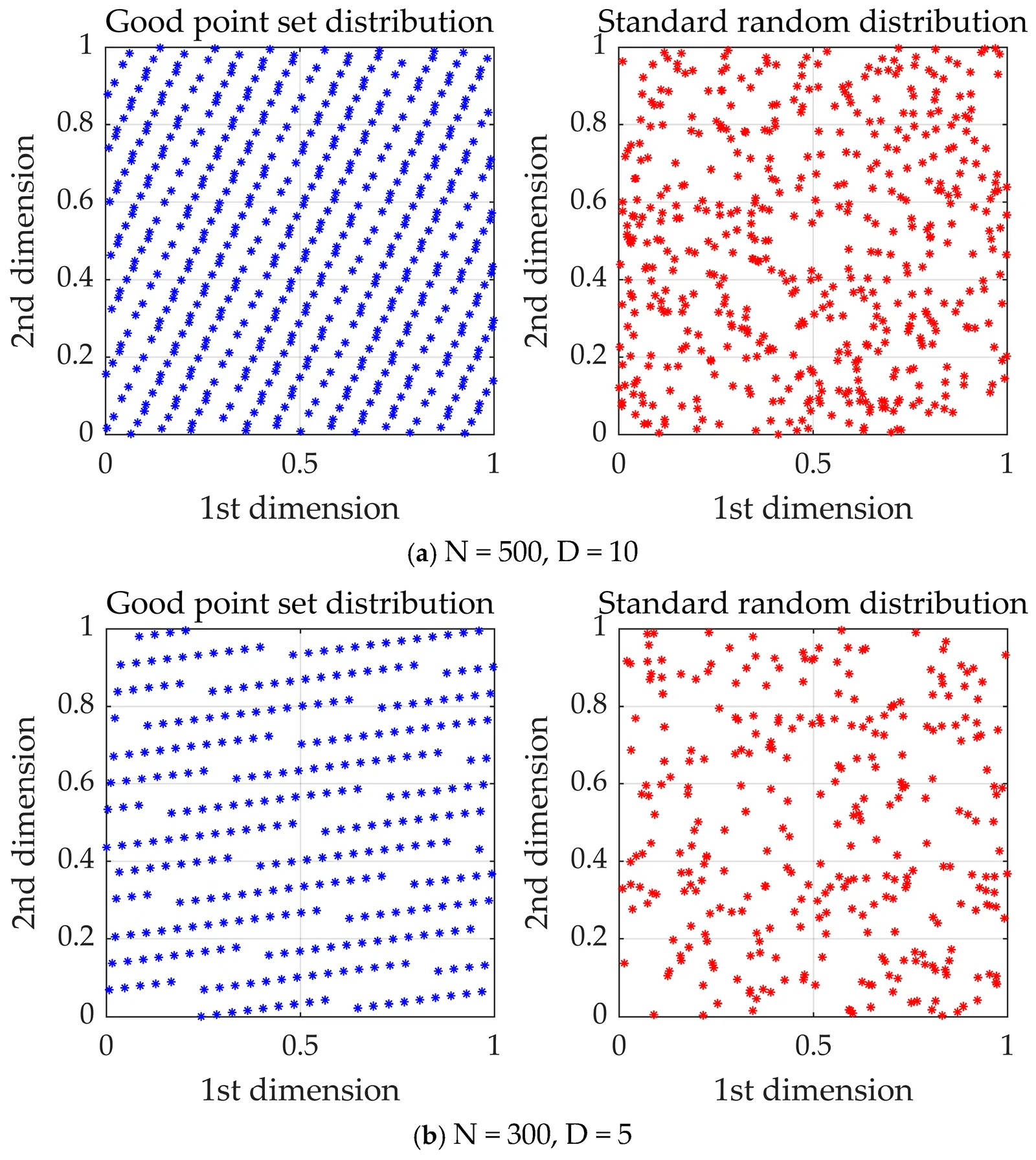
Comparison diagram of population generation distribution between good point set and standard random: (**a**) population size: 500, dimension: 10; (**b**) population size: 300, dimension: 5.

Figure 1 presents the population distribution obtained by the good point set strategy and the standard random strategy. Compared with the standard random strategy, the good point set strategy exhibits superior spatial distribution uniformity. Under different population sizes and dimensional settings, the good point set consistently maintains a stable and uniform distribution pattern. Therefore, using the good point set to update the initial population positions can effectively alleviate the problem of uneven initial population distribution.

#### 2.2.2. Random Reuse Strategy

As the iterations proceed, some sand cat individuals may suffer from insufficient search in certain dimensions, resulting in low overall search accuracy. To address this problem, an adaptive random reuse strategy is proposed in this paper. In this strategy, part of the information of the current individual is replaced with the information of the best individual with a certain probability. This improves the overall convergence accuracy and efficiency of the algorithm.

To prevent the random reuse strategy from excessively relying on the current best solution in the early stage of the algorithm, an adaptive triggering probability *P_R_* is introduced. The random reuse strategy is activated when the generated random number is less than *P_R_*:

(11) $$P_{R}=P_{\min}+\left(P_{\max}-P_{\min}\right)\frac{t}{T}$$

where *P*_min_ and *P*_max_ denote the minimum and maximum probabilities of activating the random reuse strategy, respectively. This design reduces the frequency of random reuse in the early stage of iteration, allowing the algorithm to focus mainly on global exploration. In the later stage, the frequency of random reuse is increased to enhance the utilization of the best individual and improve convergence accuracy. In addition, to enable individuals with different qualities to have different learning abilities, a fitness difference factor is introduced to further improve population diversity:

(12) $$\lambda =\frac{F_{i}-F_{best}}{F_{worst}-F_{best}}$$

where *F_i_*, *F_best_*, and *F_worst_* denote the fitness of the *i*-th individual, the current best fitness, and the current worst fitness, respectively. When the fitness of an individual is poor, its learning degree from the best solution increases. Otherwise, the learning degree decreases. Accordingly, the improved position update formula of the random reuse strategy is expressed as follows:

(13) $$\left\{\begin{array}{ll} X_{i,j}^{\left(t+1\right)}=X_{i,j}^{best}+D\alpha \left(ub-lb\right)r_{6}\lambda & r_{5}<0.5\\ X_{i,randj}^{\left(t+1\right)}=X_{i,randj}^{\left(t\right)}+r_{6}\lambda \left(X_{randj}^{best}-X_{i,randj}^{\left(t\right)}\right) & else \end{array}\right.$$

where $X_{i,randj}^{\left(t\right)}$ denotes the randomly selected *j*-th dimensional variable of the *i*-th individual and the $X_{randj}^{best}$ denotes the randomly selected *j*-th dimensional variable of the current best individual. *D* is a direction coefficient, and its value is either 1 or −1, controlled by a random number; *r*_5_ and *r*_6_ are random numbers in [0, 1]. The perturbation parameter $\alpha =5e^{-16{\left(t/T\right)}^{2}}/\sqrt{2\pi }$ is related to the iteration time. The parameter *α* is relatively large in the early stage of iteration, which ensures a large perturbation near the optimal solution and enhances the exploration capability of the algorithm. As *α* gradually decreases, the perturbation is reduced, thereby improving the accuracy of local exploitation. This strategy combines randomness with the inheritance of optimal information. It can enhance the utilization of promising search directions and guide some individuals to move toward the best individual, thereby improving convergence efficiency.

#### 2.2.3. Prey Impatience Coefficient

In the exploitation phase, the original SCSO algorithm mainly relies on random parameters and the current best individual to guide local exploitation. However, it lacks a feedback mechanism for regulating the degree of population aggregation. In real hunting scenarios, when a sand cat approaches its prey, the prey tends to become highly agitated and escape rapidly. When the sand cat is far from the prey, the prey remains relatively stable. From the perspective of the prey, this paper designs an impatience coefficient to control the exploitation capability of the population. First, the minimum Euclidean distance *d*_min_ between all individuals and the current best individual is defined. The search-space distance scale (M_c) is also defined as follows:

(14) $$d_{\min}=\min\left({\left\|X_{i}-X_{best}\right\|}_{2}\right)$$

(15) $$M_{c}=\frac{{\left\|ub-lb\right\|}_{2}}{N}$$

where || ||_2_ denotes the Euclidean norm of a vector and *N* denotes the population size. The impatience coefficient is then defined as follows:

(16) $$z=e^{-\frac{d_{\min}}{M_{c}}}$$

where z∈(0,1]. To avoid the trivial zero distance caused by the current best individual itself, the best individual is excluded when calculating the *d*_min_. Equation (16) indicates that when individuals approach the optimal solution, *d*_min_ becomes smaller and *z* becomes larger. This mechanism prevents excessive population aggregation, maintains local search activity, and reduces the risk of premature convergence. When the population is relatively far from the current best solution, a smaller value of *z* weakens the local perturbation and preserves a broader search range. Accordingly, Equation (7) is modified as follows:

(17) $$X(t+1)=X_{best}(t)-r\cdot (1+z)\left|r_{5}\cdot X_{best}(t)-X(t)\right|\cos\theta$$

This strategy dynamically adjusts the local search intensity according to the distance between the population and the optimal solution. It helps improve the convergence speed of the algorithm in the later stage.

#### 2.2.4. Opposition-Based Learning for the Worst Individual

During the iterative process of swarm intelligence algorithms, some individuals with poor fitness may remain in low-quality search regions for a long time. This reduces population diversity. To reduce the influence of inferior individuals on population evolution, an opposition-based learning strategy is introduced in this paper to update the worst individual. The basic idea of opposition-based learning is to expand the search range by considering the opposite solution corresponding to the current solution. This helps improve the global search capability and convergence speed of the algorithm. This method was first proposed by Tizhoosh H. R. et al. However, the opposite solutions generated by traditional opposition-based learning lack randomness. Therefore, it is difficult to effectively balance population diversity and convergence within the search space.

**Figure 2:**
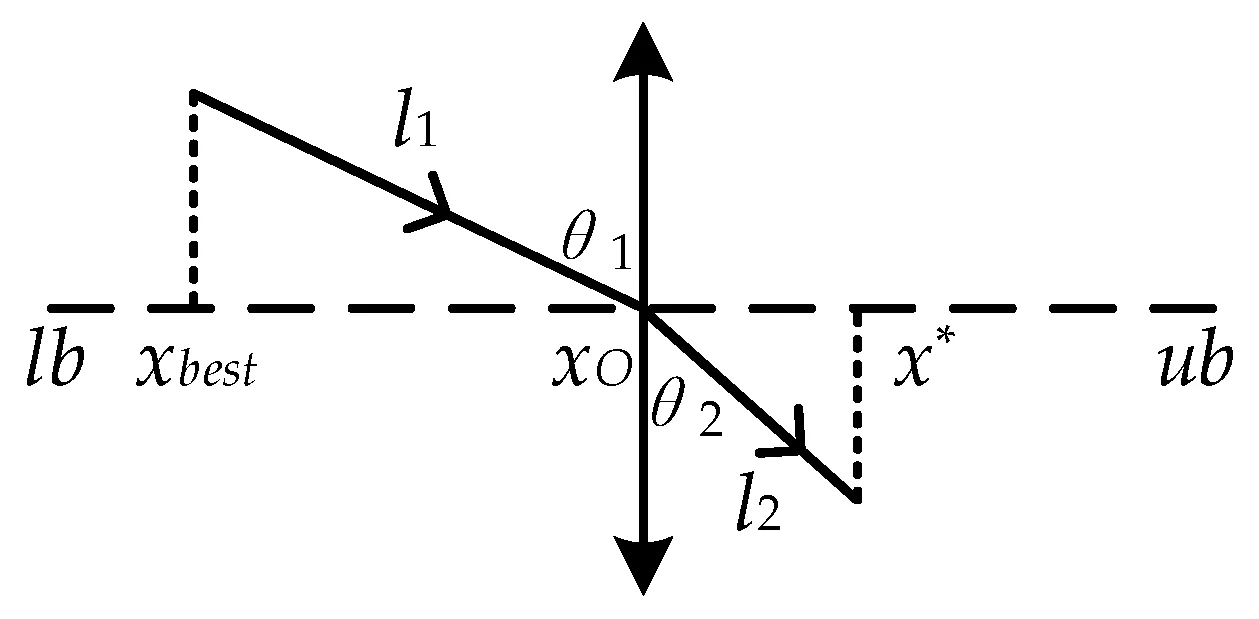
Schematic diagram of light refraction.

In this paper, the law of refraction is introduced into the opposition-based learning strategy. Through light refraction, the corresponding opposite solution is generated. This serves to widen the exploration scope of the algorithm while mitigating the risk of premature convergence to local optima. The principle is shown in Figure 2.

According to Snell’s law, the following relationship can be obtained:

(18) $$\frac{\sin\theta_{1}}{\sin\theta_{2}}=n_{21}$$

where *θ*_1_ and *θ*_2_ denote the angle of incidence and the angle of refraction, respectively, and *n*_21_ denotes the relative refractive index of the medium. According to the geometric relationships shown in the figure, the following equations can be obtained:

(19) $$\sin\theta_{1}=\frac{x_{O}-x_{best}}{l_{1}}$$

(20) $$\sin\theta_{2}=\frac{x^{*}-x_{O}}{l_{2}}$$

(21) $$x_{O}=\frac{ub+lb}{2}$$

where *x_best_* denotes the current best solution, x* denotes the opposite solution, *x_O_* denotes the refraction point, and *l*_1_ and *l*_2_ denote the lengths of the incident ray and the refracted ray, respectively. Based on the above equations, the following expression can be derived:

(22) $$x^{*}=x_{O}+\frac{x_{O}-x_{best}}{m}$$

where *m* = *n*_21_∙*l*_1_/*l*_2_. To simulate the refraction phenomenon in different media, *m* is defined as tan(rand*π/2). If the opposite solution is better than the current worst individual, the worst individual is replaced by the opposite solution. Otherwise, the original individual is retained.

### 2.3. Flowchart and Pseudocode of CMSCSO

The standard SCSO primarily maintains population diversity through random parameters and randomly generated search angles. As the iterations proceed, the population gradually converges toward the current best solution, which increases the risk of becoming trapped in a local optimum. CMSCSO alleviates this limitation through the synergistic integration of multiple mechanisms. The good point set initialization strategy enables the initial individuals to be distributed more uniformly throughout the search space. This increases the probability of exploring different regions during the early search stage and primarily reduces the risk of premature convergence. The adaptive random reuse strategy reconstructs selected dimensions of each individual. The triggering probability of this mechanism varies dynamically with the iterative process, while the reuse intensity is adaptively adjusted according to the fitness difference of each individual. Consequently, different individuals can retain distinct portions of their original feature information and generate diverse dimensional combinations. This mechanism continuously introduces small perturbations into the population, thereby preventing excessive population contraction and aggregation. The opposition-based learning based on the refraction mechanism is applied to the current worst individual. An opposite candidate solution is generated in another region of the search space. The original worst individual is replaced when the opposite solution has better fitness; otherwise, the original individual is retained. This operation opens a new search direction without disturbing high-quality individuals and provides a direct means of escaping the current local optima of attraction. Finally, the prey impatience coefficient dynamically adjusts the local search intensity according to the distance between the population and the current best solution. This enables the algorithm to adopt a search scale that matches the current population distribution and improves its fine-grained optimization capability in newly identified promising regions.

**Figure 3:**
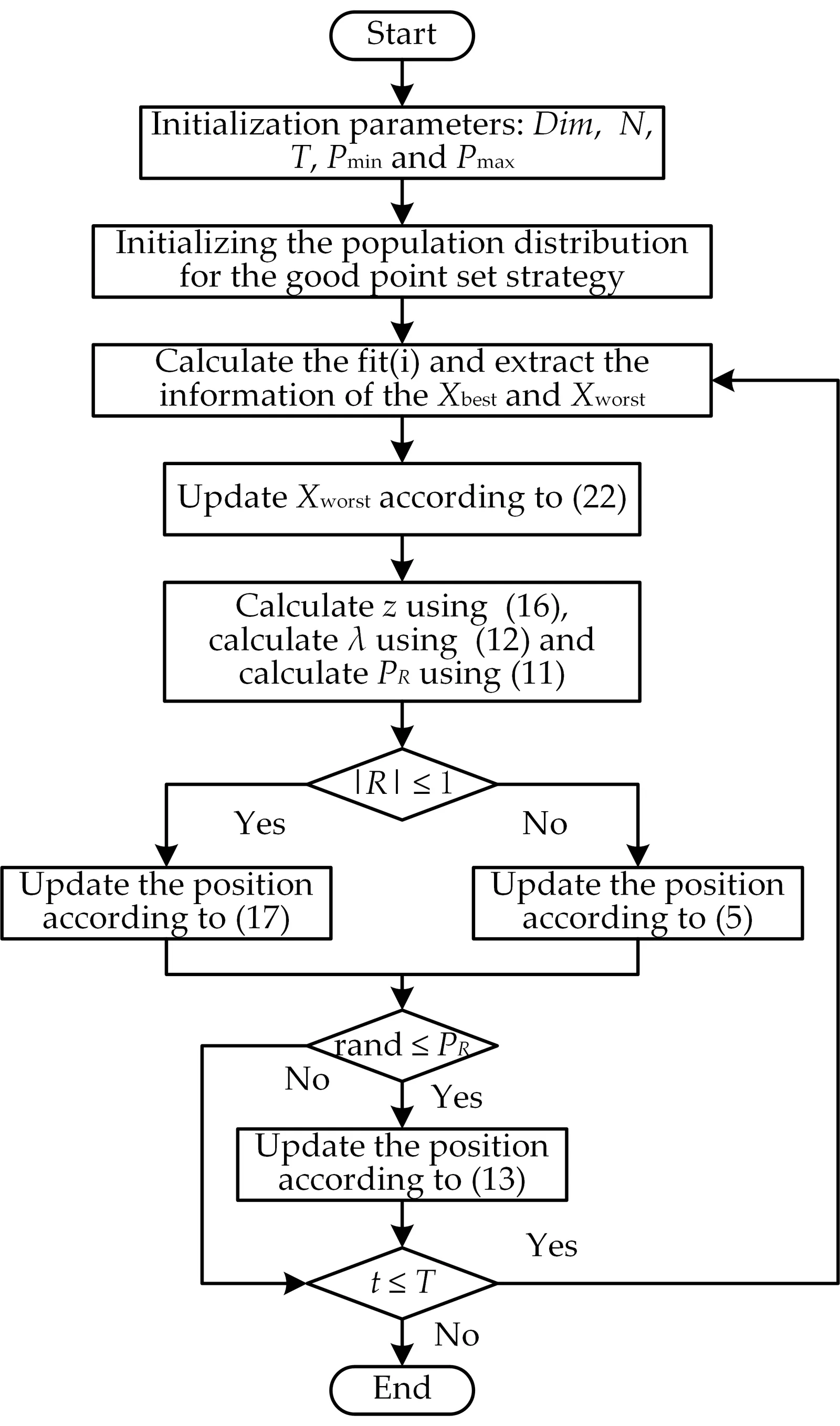
Flowchart of CMSCSO.

Based on the above analysis, the flowchart of CMSCSO is shown in Figure 3, and its pseudocode is given in Algorithm 1. After the algorithm starts, CMSCSO first performs parameter initialization, including the problem dimension, population size, maximum number of iterations, and the probability parameters of the random reuse strategy. Then, the good point set strategy is used to generate the initial population. This allows the initial individuals to be distributed as uniformly as possible within the constrained search space, thereby improving the traversal capability of the algorithm over the solution space. After calculating the fitness values, CMSCSO collects the information of the best and worst individuals. This information provides the input for the subsequent optimization strategies.

On this basis, the opposite position of the worst individual is updated according to (22). The fitness value of the opposite solution is compared with that of the worst individual to determine whether the opposite solution should be retained. This process introduces a higher-quality candidate and improves the overall population quality. Subsequently, the impatience coefficient *z*, the fitness difference factor λ, and the triggering probability *P_R_* of the random reuse strategy are calculated using (16), (12), and (11), respectively. This completes the adaptive parameter update process within a single iteration.

The search behavior of the sand cats is determined according to the value of |*R*|. When |R| > 1, the algorithm enters the global exploration mode, and individual positions are updated using (5). When |R| ≤ 1, the algorithm enters the local exploitation mode, and individual positions are updated using (17). After the above position update is completed, *P_R_* is used to determine whether the random reuse strategy is triggered. If it is triggered, the position is updated according to (13). Otherwise, the current individual position is retained. Finally, CMSCSO determines whether the termination condition is satisfied. If the condition is satisfied, the optimal value and the best individual are output. Otherwise, the algorithm continues to the next iteration.
**Algorithm 1:** Pseudocode of CMSCSO**Input:** Population size *N*, maximum iterations *T*, variable dimension *Dim*, variable lower bound *lb*, variable upper bound *ub*, objective function *fobj* **Output:** Best fitness *Fit_best_*, best position *X_best_*  1:   Initialize the population positions using the Good Point Set method.  2:   Initialize *Fit_best_* and *X_best_*  3:   **while** *t* ≤ *T* **do**  4:        **for** *i* = 1 to *N* **do**  5:          Perform boundary control on individual *X_i_*  6:          Calculate the fitness value *Fit*(*i*)  7:          **if** *Fit*(*i*) < *Fit_best_* **then**  8:            *Fit_best_* = *Fit*(*i*)  9:            *X_best_* = *X_i_*  10:             **end if**  11:        **end for**  12:        Find the worst individual *X_worst_* in the current population  13:        Update the opposite position X* for *X_worst_* according to (22)  14:        Calculate the prey impatience coefficient *z*  15:        Calculate the SCSO sensitivity parameter *r_s_*  16:        Calculate the random reuse parameters *P_R_*   17:        **for** *i* = 1 to *N* **do**  18:             Generate random parameters *r* and *R*  19:             Calculate the fitness difference factor *λ*  20:             **for** *j* = 1 to *d* **do**  21:               Select the search angle *θ* via roulette wheel selection  22:               **if** |*R*| ≤ 1 **then**  23:                 Update the position according to (17)  24:               **else**  25:                 Update the position according to (5)  26:               **end if**  27:             **end for**   28:             **if** rand < *P_R_* **then**  29:               Update the position according to (13)  30:             **else**  31:               Retain the information of the original individual  32:             **end if**  33:        **end for**   34:        *t = t + 1*  35:          **end while**  36:          **return** *Fit_best_* and *X_best_*

### 2.4. CMSCSO Complexity Analysis

The population size of CMSCSO is *N*, the problem dimension is *D*, and the maximum number of iterations is *T*. In the initialization phase, CMSCSO adopts the good point set strategy, whose time complexity is *O*(*ND*).

During each iteration, the algorithm mainly performs worst-value opposition-based learning, prey impatience coefficient calculation, position update, and random reuse operations. The time complexities of prey impatience coefficient calculation, position update, and random reuse are all *O*(*ND*). Worst-value opposition-based learning generates only one additional candidate solution and performs one additional fitness evaluation in each iteration. Therefore, it does not change the overall asymptotic complexity of the algorithm. In summary, when the computational complexity of the objective function is approximately *O*(*D*), the overall time complexity of CMSCSO is *O*(T*ND*). The space complexity is mainly determined by the population matrix, the good point set matrix, and the best solution. Therefore, the overall space complexity is *O*(*ND*).

Compared with the original SCSO, the proposed algorithm does not change the time complexity or space complexity. It only introduces a limited constant-level computational overhead.

## 3. Performance Evaluation and Analysis

### 3.1. Introduction to Benchmark Functions

In this paper, the CEC2017 benchmark test suite [38] and the CEC2022 benchmark test suite [39] are used to evaluate the performance of CMSCSO. Several recently proposed innovative algorithms are also included for comparison. These algorithms include the BBO, CAOA, FGO, GGO, MShOA, POA, and ZOA. All comparison algorithms use their original parameter settings. The specific parameters are listed in the following Table 2.

The CEC2017 and CEC2022 benchmark test suites are optimization benchmark platforms released by the IEEE Congress on Evolutionary Computation. They are widely used to comprehensively evaluate the performance of intelligent optimization algorithms. The CEC2017 benchmark test suite contains 29 multidimensional test functions, which are divided into four categories: unimodal functions, multimodal functions, hybrid functions, and composition functions.

Compared with CEC2017, the number of functions in CEC2022 is reduced from 29 to 12. However, the function structures are more complex. Therefore, CEC2022 can better simulate various challenges in real-world optimization problems. All algorithm tests in this paper are conducted on a Windows platform with a 4.7 GHz CPU and 32 GB of memory. The maximum number of iterations for the algorithm is 500, and the population size is 30. To avoid the randomness of the test results, each algorithm is independently run 30 times. The minimum value, average value, and standard deviation are then calculated.

### 3.2. CEC2017

#### 3.2.1. Optimization Result Analysis

Table 3 presents the test results on the 30-dimensional CEC2017 benchmark test suite. All best results are highlighted. The following observations can be made from the table.

For the unimodal test functions F1 and F3, CMSCSO achieves the best average values. This demonstrates that CMSCSO has good convergence ability and high search accuracy. This improvement is mainly attributed to the introduction of the impatience coefficient. It enhances the search intensity around the current best solution during the exploitation phase. As a result, CMSCSO can better exploit the optimal solution.

For the multimodal test functions F4–F10, CMSCSO obtains the best average values on all functions. The random reuse strategy and opposition-based learning strategy in CMSCSO enhance the global search capability and effectively improve the information of the worst individuals. Therefore, the algorithm can effectively alleviate premature convergence on multimodal functions. However, CMSCSO does not achieve the smallest standard deviation on all multimodal test functions. The BBO obtains smaller standard deviations on most multimodal functions, but its average values are relatively poor. This indicates that the BBO stably converges to inferior regions. In contrast, CMSCSO has larger fluctuations in some cases, but its overall optimization quality is higher.

Hybrid functions are composed of several basic functions and have more complex search spaces. For the hybrid functions F11–F20, CMSCSO performs well on most test functions, such as F11–F19. This indicates that the multi-strategy collaborative mechanism of CMSCSO is effective for complex hybrid functions. The good point set initialization provides a more uniform search basis for the algorithm. The random reuse strategy enhances the perturbation capability of the algorithm. Opposition-based learning continuously improves the worst individuals. The impatience coefficient improves the accuracy of local exploitation in the later stage of iteration. However, CMSCSO does not obtain the best average values on F15, F18, and F20. This is because several improvement strategies in CMSCSO rely on the current best solution. In the later stage of the algorithm, these strategies may cause excessive search around this region, resulting in a decrease in population diversity. For the remaining composition functions, CMSCSO achieves the best average values on all functions. This demonstrates that CMSCSO has good comprehensive optimization capability and robustness.

Overall, CMSCSO shows strong competitiveness on the CEC2017 benchmark test suite. These results verify the correctness, adaptability, and robustness of the proposed method.

**Figure 4:**
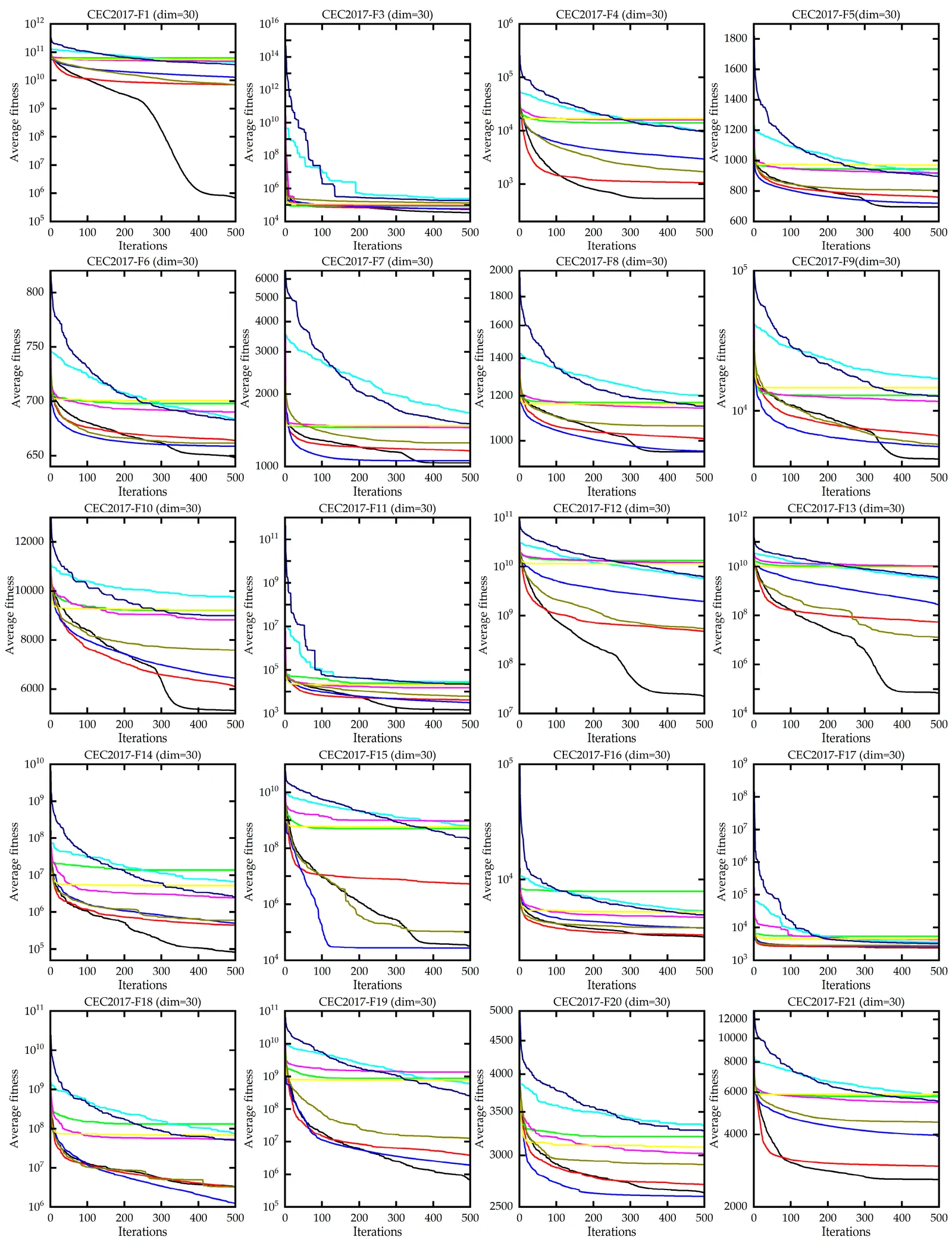

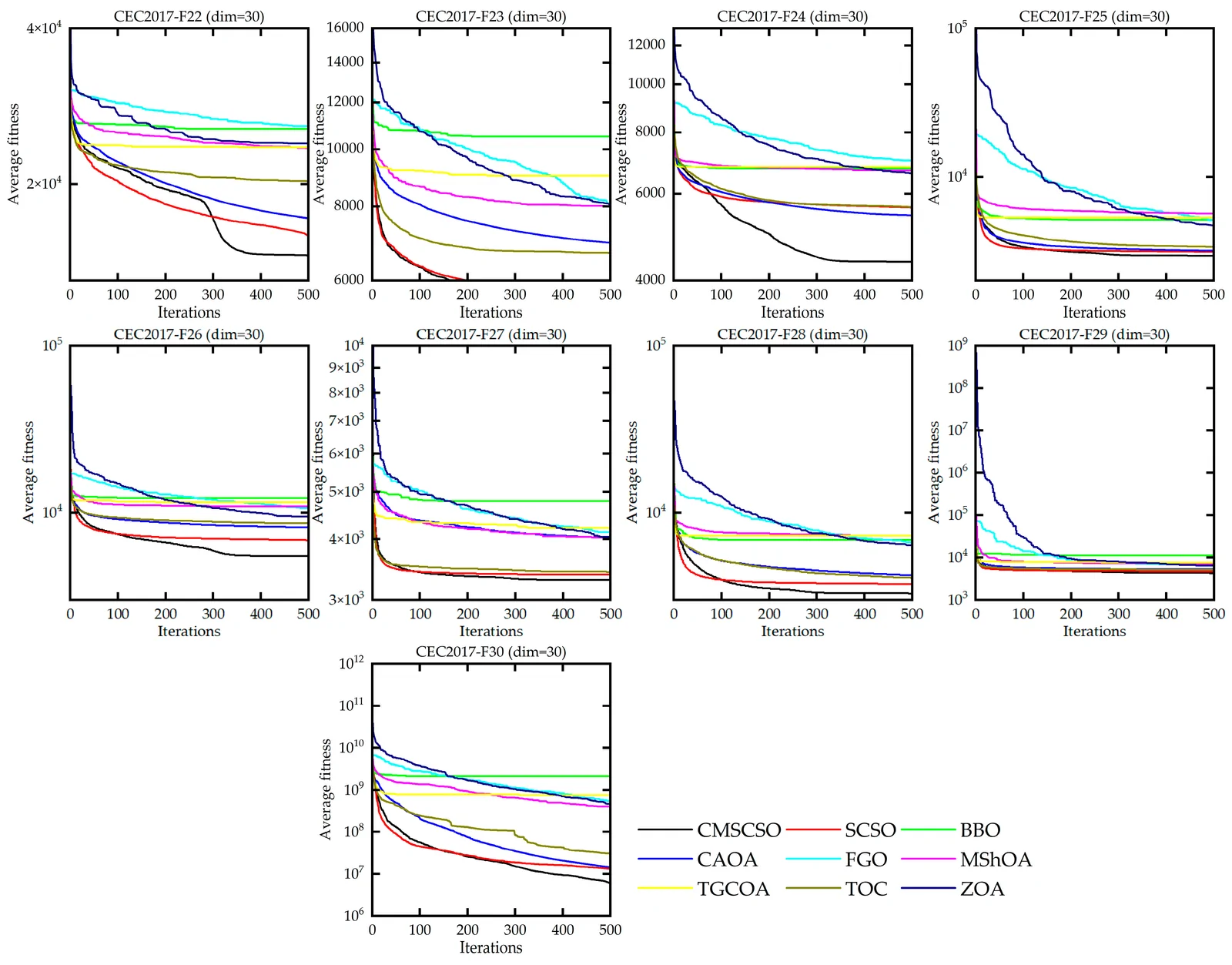
Convergence profiles of various algorithms on selected CEC2017 (dim = 30) benchmark test functions.

Figure 4 presents the average fitness curves of CMSCSO and the comparison algorithms on 29 dim = 30 CEC2017 benchmark functions.

For the unimodal functions F1 and F3, CMSCSO exhibited excellent local refinement capability. On F1, CMSCSO did not achieve the fastest convergence during the early iterations. However, the comparison algorithms gradually stagnated after their fitness values reached approximately 10^9^–10^10^, whereas CMSCSO continued to improve the solution. Its final fitness value was several orders of magnitude lower than those of the comparison algorithms. This result indicates that CMSCSO retains sufficient exploitation capability during the middle and later stages of the search. On F3, CMSCSO rapidly reduced the fitness value during the early iterations and subsequently maintained effective fine-grained exploitation, eventually converging to the lowest fitness level. The results on F1 and F3 demonstrate that CMSCSO can accurately exploit promising regions after the principal search direction has been identified.

For the simple multimodal functions F4-F10, CMSCSO exhibited both rapid early convergence and sustained search capability during the middle and later stages. On F4, the convergence curve of CMSCSO declined rapidly during the early iterations and continued to decrease after several comparison algorithms had entered a stable phase, ultimately reaching the lowest fitness value. On F5–F10, the convergence curves of CMSCSO exhibited secondary declines, indicating that the population could continue to refine the search space. These results demonstrate that CMSCSO can maintain search activity in multimodal landscapes and has a lower probability of becoming trapped in local optima.

For the hybrid functions F11–F20, algorithmic performance was strongly dependent on the characteristics of each function. These functions combine multiple basic landscape structures and exhibit stronger coupling among variables. CMSCSO showed particularly strong performance on most functions, except F15, F18, and F20. On F11, its convergence curve declined rapidly during the early iterations, continued to improve during the middle stage, and ultimately reached the lowest fitness value. On F12 and F13, CMSCSO exhibited substantial reductions during the middle and later stages, whereas most comparison algorithms remained at considerably higher fitness levels. On F14 and F19, the convergence curves of CMSCSO continued to decrease and eventually achieved the best convergence accuracy. Nevertheless, CMSCSO did not achieve an absolute advantage on every function. On F15, F18, and F20, its convergence performance remained competitive, although the final results were slightly inferior to those of the CAOA. This phenomenon indicates that the proposed strategies improve the overall search performance of SCSO, although the magnitude of improvement varies with the landscape structures of the hybrid functions.

For the composition functions F21–F30, CMSCSO generally achieved the lowest or nearly the lowest convergence curves. On F21, CMSCSO rapidly identified promising solutions during the early iterations and continuously refined them throughout the remaining search process. On F22, most comparison algorithms had already entered stable plateaus, whereas CMSCSO continued to perform effective exploitation. On F23 and F24, the convergence curves of CMSCSO declined continuously, and the final fitness values were lower than those obtained by all comparison algorithms. On F25-F30, CMSCSO similarly combined rapid early convergence with sustained improvement during the later iterations.

Overall, the convergence curves of CMSCSO continued to decline during the middle and later stages of the iterative process, which is consistent with the intended design of the proposed improvement strategies. The synergistic multi-strategy mechanism provides the algorithm with more opportunities to generate and evaluate promising candidate solutions.

#### 3.2.2. Exploration and Exploitation Analysis

**Figure 5:**
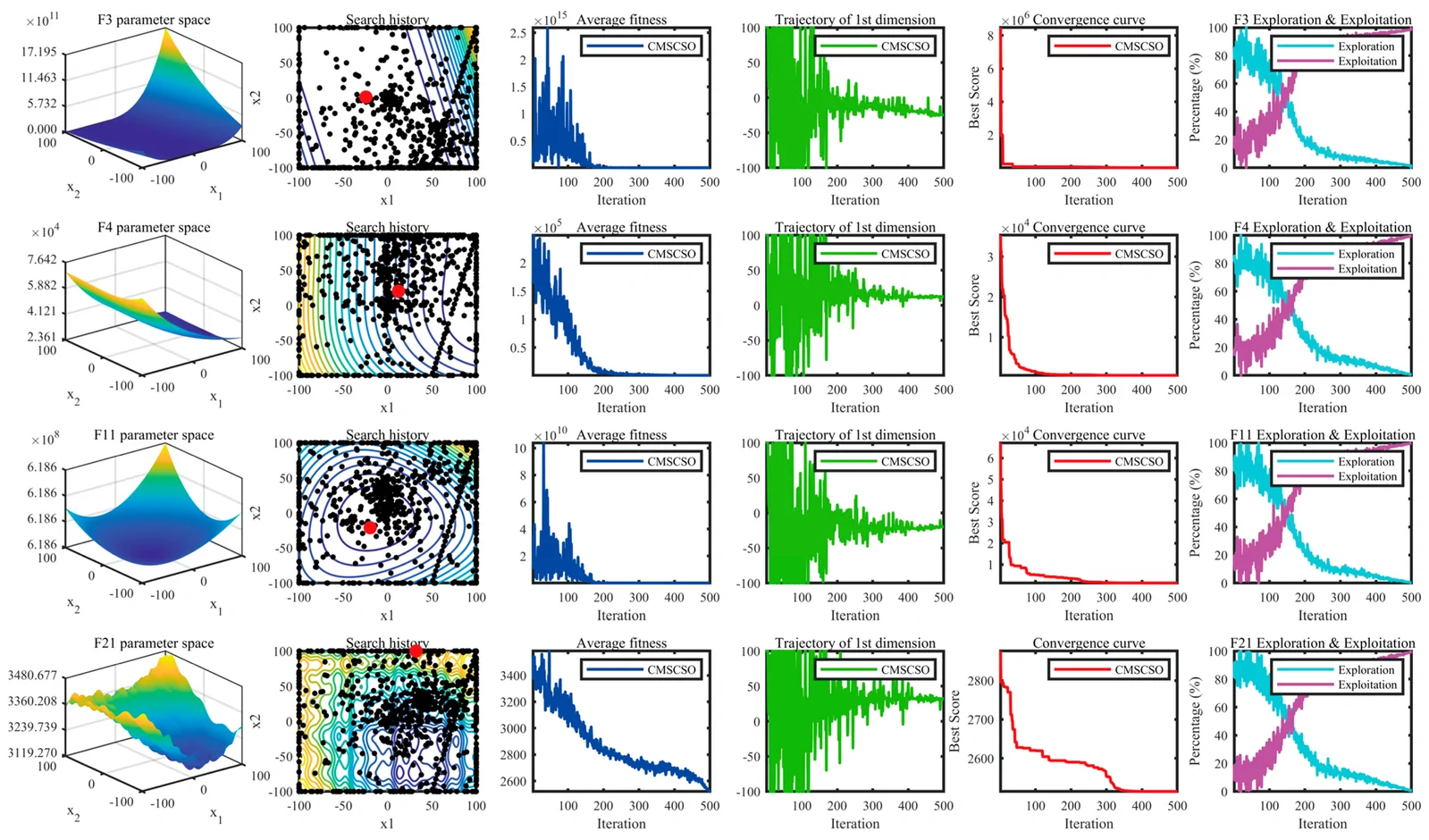
Qualitative search-behavior analysis of CMSCSO on dim = 30 CEC2017. (From left to right: two-dimensional landscape visualization, search history, population-average fitness, the trajectory of the first dimension, best-so-far convergence curve, and exploration–exploitation percentages).

To further analyze the exploration and exploitation behavior of CMSCSO during the optimization process, four representative functions from the dim = 30 CEC2017 test suite were selected for qualitative analysis, including the unimodal function F3, the simple multimodal function F4, the hybrid function F11, and the composition function F21. Figure 5 presents, in sequence, the three-dimensional landscapes of these functions, the search histories, the average population fitness, the trajectory of the first dimension, the convergence curves of the historical best values, and the diversity-based exploration and exploitation ratios.

The three-dimensional function landscapes and search histories were mainly used to observe the distribution and movement range of the search agents in the search space. The black points represent the historical positions visited by the algorithm throughout the entire iterative process, whereas the red circles denote the best positions obtained within the maximum number of iterations. The average population fitness reflects the evolutionary trend of the overall solution quality of all search agents. The convergence curve of the best value represents the best objective function value obtained up to the current iteration. The trajectory of the first dimension was used to observe the process by which an individual search agent gradually shifted from large-scale movement to local search.

The exploration and exploitation curves further reveal the dynamic search pattern of CMSCSO. On all representative functions, the exploration ratio remained at a relatively high level of approximately 70–95% during the early stage, whereas the exploitation ratio remained relatively low. Thereafter, the exploration ratio gradually decreased, while the exploitation ratio continuously increased, and the two crossed near the middle stage. In the later stage, the exploitation ratio gradually approached 100%, indicating that the major computational resources were devoted to the local refinement of promising regions. This trend is consistent with the search stages reflected by the average population fitness, the trajectory of the first dimension, and the convergence curves.

Overall, CMSCSO exhibited a gradual transition from global exploration to local exploitation on all four categories of test functions. For F3 and F4, the algorithm was able to rapidly narrow the search range and obtain stable solutions. For F11 and F21, the best value could still be continuously improved during the middle and later stages, indicating that the proposed random reuse strategy, predator impatience coefficient, and opposition-based learning strategy for the worst individual help maintain a certain degree of search activity. Even on the complex composition function F21, the algorithm was still able to achieve iterative convergence with satisfactory performance. Therefore, CMSCSO is capable of achieving a reasonably balanced stage-wise transition between exploration and exploitation.

### 3.3. CEC2022

Table 4 presents the test results on the 10-dimensional CEC2022 benchmark test suite. All best results are highlighted. The following observations can be made from the table.

For the unimodal test function F1, CMSCSO achieves the best results in all metrics. Specifically, the average value of CMSCSO is 300.04, and the standard deviation is only 0.09. These results are significantly better than those of the other comparison algorithms. This indicates that CMSCSO can maintain strong convergence ability and stable exploitation capability in a unimodal search environment. The good point set strategy provides a better population distribution, which enables the algorithm to obtain a more stable initial search basis. The impatience coefficient further improves the exploitation efficiency around the optimal region, thereby achieving better convergence accuracy.

For the basic functions, CMSCSO obtains the best average values on F2, F3, and F5. On F4, its performance is slightly worse than that of the CAOA, but the difference is small. The test results on the basic functions show that good point set initialization, worst-value opposition-based learning, and the random reuse strategy can effectively improve population quality and search efficiency. These strategies not only enable the algorithm to obtain better solutions but also make the convergence results more stable.

For the hybrid functions F6-F8, CMSCSO fails to obtain the best average value only on F6. This indicates that CMSCSO still has good search capability in hybrid search spaces. On F6, CMSCSO may be affected by local optima, which weakens its global search capability.

For the composition functions F9-F12, CMSCSO shows the most outstanding overall performance and obtains the best average values on all functions. Composition functions usually have more complex search spaces and more local optimum traps. Therefore, they can reflect the adaptability of an algorithm in solving complex optimization problems. The results of CMSCSO on these functions indicate that, under the multi-strategy collaborative mechanism, CMSCSO can maintain good optimization accuracy and robustness.

Based on the overall results of the 10-dimensional CEC2022 benchmark test suite, CMSCSO obtains the best average values on 10 out of 12 test functions and achieves the smallest standard deviations on seven functions. Compared with the original SCSO, CMSCSO performs slightly worse only on F4. These results indicate that the proposed improvement strategies not only enhance the optimization accuracy of the algorithm but also significantly improve its operational stability in low-dimensional test environments.

### 3.4. Computational Efficiency Analysis

In addition to optimization accuracy, computational efficiency is another important criterion for evaluating metaheuristic algorithms. Therefore, the average running times of CMSCSO and the standard SCSO were compared on the dim = 30 CEC2017 and dim = 10 CEC2022 benchmark suites. The two algorithms share the same basic framework. This comparison therefore provides a direct measurement of the computational overhead introduced by the proposed strategies. The experimental results are reported in Table 5.

Across all 29 functions in the 30-dimensional CEC2017 benchmark suite, the average running times of CMSCSO and the standard SCSO were 0.2137 and 0.2027 s, respectively. Thus, CMSCSO introduced an average time overhead of approximately 5.43%. On the 10-dimensional CEC2022 benchmark suite, their average running times were 0.0618 and 0.0583 s, respectively, corresponding to an additional computational overhead of approximately 6.00%.

The additional computational cost mainly arises from the calculation of the predator impatience coefficient and fitness difference factor, the random reuse operation, and the generation and fitness evaluation of opposition-based candidate solutions for the worst individual. Despite these additional operations, CMSCSO and the standard SCSO have the same asymptotic time complexity of *O*(*TND*) and space complexity of *O*(*ND*). The measured increase in running time was limited to approximately 5–6%.

A comprehensive analysis of the benchmark results (Table 3 and Table 4) and convergence curves (Figure 4) indicates that CMSCSO significantly improves optimization accuracy and algorithmic stability while incurring only a negligible computational cost.

### 3.5. Nonparametric Tests

The test results in Table 3 and Table 4 preliminarily demonstrate the effectiveness of CMSCSO. To avoid the influence of random factors and verify the performance differences between CMSCSO and the other comparison algorithms, two nonparametric statistical tests are further used in this paper to analyze the experimental results. These tests are the Wilcoxon rank-sum test and the Friedman test.

Table 6 and Table 7 present the Wilcoxon rank-sum test results of CMSCSO against the other comparison algorithms on the 30-dimensional CEC2017 benchmark test suite and the 10-dimensional CEC2022 benchmark test suite, respectively. The test results with *p*-values greater than 0.05 are marked in bold. The last row of each table reports the Win/Tie/Loss statistics.

The Win/Tie/Loss statistics, together with the *p*-values of the Wilcoxon test and the average fitness values, can intuitively reflect the number of wins, ties, and losses of CMSCSO over all test functions. When the *p*-value is less than 0.05 and the average value of CMSCSO is smaller, CMSCSO is considered to have better performance. Otherwise, the comparison algorithm performs better.

In the 232 comparisons on the 30-dimensional CEC2017 benchmark test suite, CMSCSO significantly outperforms the comparison algorithms in 220 cases. It shows no significant difference in 10 cases and is inferior to the comparison algorithms in only two cases. This indicates that the advantage of CMSCSO on the 30-dimensional CEC2017 benchmark test suite has strong statistical significance.

In the 96 comparisons on the 10-dimensional CEC2022 benchmark test suite, CMSCSO achieves 90 significant wins, three ties, and three losses. This demonstrates that CMSCSO still has a statistical advantage on the low-dimensional test suite.

**Figure 6:**
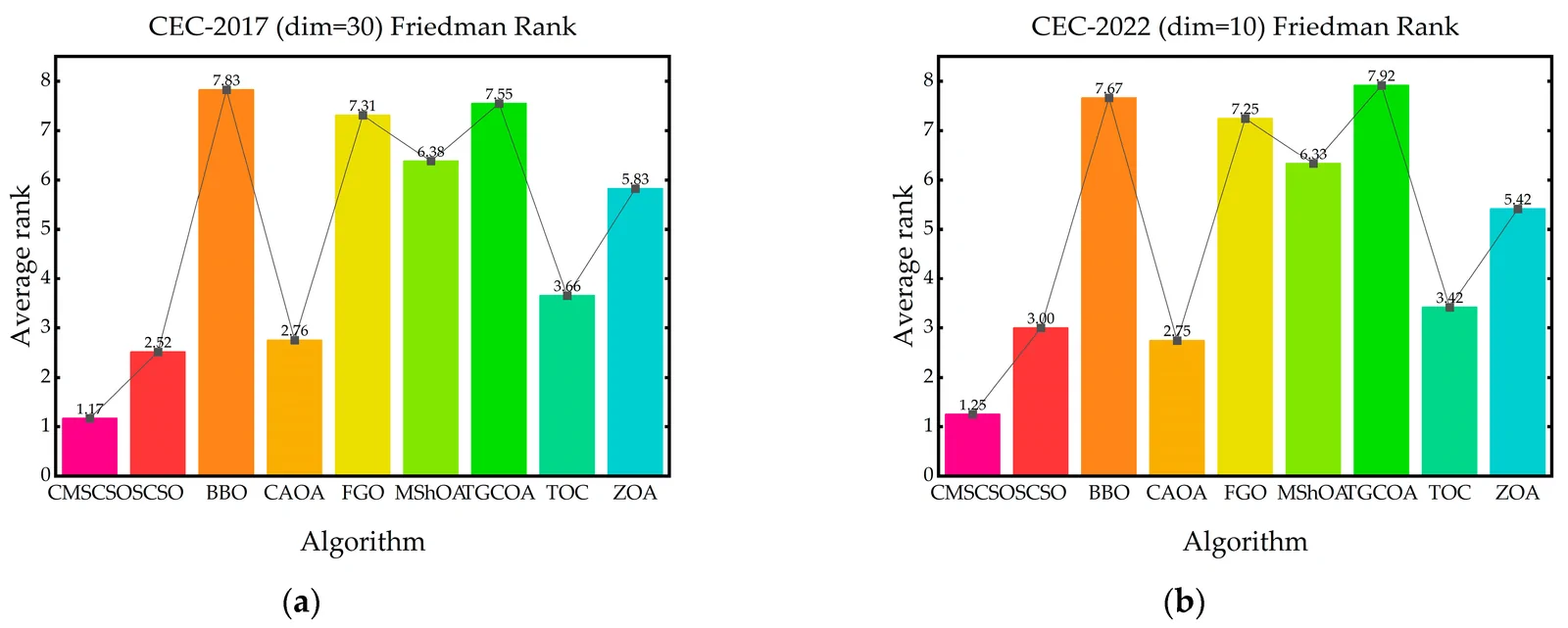
Friedman average rank results, (**a**) CEC-2017; (**b**) CEC-2022.

The Friedman test is usually used to compare the overall performance of multiple algorithms on a test suite. It does not directly compare the original fitness values. Instead, it ranks the performance of each algorithm and then calculates the average ranking over all functions. A lower Friedman ranking indicates better algorithm performance. The Friedman average ranking results of CMSCSO and the other comparison algorithms are shown in Figure 6.

As shown in Figure 6, on the CEC2017 benchmark test suite with dim = 30, CMSCSO obtains an average ranking of 1.17 and ranks first among all algorithms. It is significantly superior to the original SCSO, CAOA, and TOC and the other comparison algorithms. This demonstrates that CMSCSO has strong comprehensive optimization capability in high-dimensional complex optimization problems.

Similarly, on the CEC2022 benchmark test suite with dim = 10, CMSCSO also ranks first. Based on the Friedman ranking results of the two test suites, CMSCSO achieves the lowest ranking in both cases. This verifies its good robustness.

### 3.6. Ablation Experiment

To verify the effectiveness of the proposed strategies in improving the performance of CMSCSO, an ablation experiment is designed in this paper. CMSCSO mainly consists of good point set initialization, worst-value opposition-based learning, the prey impatience coefficient, and the random reuse strategy.

To analyze the contribution of each strategy, several SCSO variants containing only a single strategy are constructed. These variants include SCSOGPS (with the good point set strategy), SCSOOBL (with the worst-value opposition-based learning strategy), SCSOPIC (with the prey impatience coefficient), and SCSORRS (with the random reuse strategy). All ablation experiments are conducted under the same conditions.

The ablation experiments are performed using the dim = 10 CEC2022 test suite. The strategy configurations of SCSO, CMSCSO, and the ablation variants are listed in Table 8.

Each algorithm is independently run 30 times, and its average value and standard deviation are calculated. The statistical results are shown in Table 9. The Friedman ranking results are presented in Table 10.

For the unimodal function F1, the standard SCSO obtained a mean value of 2484.09 and a standard deviation of 2206.77. Among the single-strategy variants, SCSORRS achieved the best performance, reducing the mean value to 343.14 and the standard deviation to 41.38. SCSOPIC also significantly improved the performance of the standard SCSO. CMSCSO further reduced the mean value to 300.04 and the standard deviation to 0.09. These results indicate that the random reuse strategy plays an important role in improving convergence accuracy, whereas the synergistic integration of the four strategies substantially enhances convergence stability.

For the basic functions F2-F5, CMSCSO achieved the best mean value and the smallest standard deviation on every function. SCSOPIC and SCSORRS generally outperformed the standard SCSO on F2, F3, and F5. This finding demonstrates the effectiveness of adaptive local search regulation and dimension-level information reuse for these functions. However, none of the four single-strategy variants surpassed the standard SCSO on F4. The mean values obtained by SCSOGPS, SCSOOBL, SCSOPIC, and SCSORRS were 826.78, 828.44, 828.61, and 826.39, respectively, whereas the standard SCSO obtained a mean value of 826.31. In contrast, CMSCSO achieved the best mean value of 825.60. This result suggests that the improvement on F4 cannot be attributed to any individual strategy. Instead, it results from the complementary effects of the multi-strategy collaborative framework.

For the hybrid functions F6-F8, CMSCSO again achieved the best mean values on all three functions. On F6, SCSOGPS, SCSOOBL, and SCSOPIC improved the average performance of the standard SCSO, whereas SCSORRS alone performed worse than the standard SCSO. On F7 and F8, SCSOPIC and SCSORRS exhibited competitive performance. This finding indicates that the predator impatience coefficient and random reuse strategy are valuable for handling landscapes composed of multiple interacting functional components. Although SCSORRS obtained the smallest standard deviation on F8, CMSCSO achieved a lower mean value. Therefore, CMSCSO provided a better balance between solution quality and stability.

For the composition functions F9-F12, CMSCSO achieved the best mean values on all four functions. SCSORRS performed well on F9 and F11, whereas SCSOGPS obtained relatively competitive results on F10 and F12. SCSOPIC also improved the standard SCSO on all four composition functions. CMSCSO consistently outperformed all single-strategy variants, indicating that the four strategies provide complementary search capabilities in complex composition search spaces.

The Friedman ranking results presented in Table 10 further confirm the above observations. CMSCSO ranked first among all algorithms, with an average rank of 1.08. SCSORRS ranked second, with an average rank of 2.75, followed by SCSOPIC (3.04), SCSOGPS (4.46), the standard SCSO (4.71), and SCSOOBL (4.95). Therefore, on the CEC2022 test suite, the random reuse strategy and predator impatience coefficient produced the strongest independent improvements. The good point set strategy yielded moderate and function-dependent improvements, whereas opposition-based learning improved performance on only some functions when applied independently.

This result demonstrates that the superior performance of CMSCSO is not attributable to any single strategy. Instead, it arises from the synergistic effects of initial population distribution optimization, adaptive information reuse, distance-based local search regulation, and worst-individual reconstruction. Each strategy targets a different stage of the search process. Their collaborative application produces improvements that are considerably greater than those achieved by applying any individual strategy alone.

### 3.7. Sensitivity Analysis

Parameter sensitivity analysis is an important method for evaluating the robustness and parameter adaptability of intelligent optimization algorithms. Different control parameter settings may lead to different experimental results. Therefore, parameter tuning is required to determine the optimal parameter configuration of the algorithm. In CMSCSO, *P*_min_ and *P*_max_ control the triggering probabilities of the random reuse strategy during the early and late stages of iteration, respectively.

In this section, an experimental design framework is employed to determine the optimal parameter settings. Experiments are conducted on two selected benchmark functions from the CEC2022 test suite. The specific experimental configurations are as follows: *N* in {10, 20, 30}, dim in {10, 20}, and *P*_min_ and *P*_max_ in {(0.1, 0.9), (0.2, 0.8), (0.3, 0.7), (0.4, 0.6)}.

Table 11 and Table 12 present the sensitivity analysis results of CMSCSO on CEC2022-F2 and CEC2022-F12, respectively. F2 is a basic function, whereas F12 is a composition function. These two functions can therefore reflect the parameter response characteristics of CMSCSO in a relatively simple search space and a complex composition search space, respectively.

The combined experimental results in Table 11 and Table 12 show that (*P*_min_, *P*_max_) = (0.2, 0.8) achieved the smallest mean value in nine of the 12 parameter–test combinations. Near-optimal results were also obtained in the remaining three combinations.

The results indicate that when *P*_min_= 0.2, the random reuse strategy is not triggered excessively during the early iterations. Sufficient search capacity is therefore retained for global exploration. When *P*_max_= 0.8, the dimensional information of high-quality individuals can be adequately exploited during the later iterations. Meanwhile, excessive dependence of the entire population on the current best solution is avoided. In comparison, (*P*_min_, *P*_max_) = (0.1, 0.9) produces a larger variation in the triggering probability between the early and late stages. This setting may increase the risk of rapid population aggregation during the later iterations. By contrast, (*P*_min_, *P*_max_) = (0.4, 0.6) has a relatively narrow probability range. This may weaken the ability of the random reuse strategy to adaptively regulate the search behavior throughout the iterative process.

As the population size increased, the mean objective function values of the test functions generally decreased. This result indicates that an appropriate increase in population size can improve the coverage of the search space and enhance optimization accuracy. However, the magnitude of performance improvement gradually diminished as the population size continued to increase. Furthermore, under the same population size and parameter settings, the mean values and standard deviations obtained for the dim = 20 problems were generally larger than those obtained for the dim = 10 problems. This finding suggests that the expansion of the search space with increasing dimensionality correspondingly increases the difficulty of solving the problems using CMSCSO.

### 3.8. Scalability Analysis on Higher-Dimensional Benchmark Problems

In this section, the high-dimensional scalability of CMSCSO is evaluated using the CEC2017 test suite at two dimensionalities (dim= 50, 100). The experimental results are presented in Table 13 and Table 14. All experimental settings are consistent with those described above.

The results presented in Table 13 and Table 14 demonstrate that CMSCSO maintains strong performance as the problem dimensionality increases substantially. For the 50-dimensional problems, CMSCSO achieved the best mean values on 27 of the 29 functions and ranked first with an average Friedman rank of 1.07. For the 100-dimensional problems, CMSCSO obtained the best mean values on 26 functions and again ranked first according to the Friedman test.

The high-dimensional scalability experiments indicate that CMSCSO retains high optimization accuracy and the best Friedman ranking when the problem dimensionality increases from 30 to 100. Although solution quality deteriorated on certain functions as the dimensionality increased, the relative advantage of CMSCSO over the comparison algorithms remained stable. Therefore, CMSCSO exhibits good scalability and robustness over the investigated range of dimensionalities.

## 4. Engineering Optimization Evaluation and Analysis

To further verify the practical application value of CMSCSO in engineering problems, this paper conducts performance validation for three types of engineering optimization problems. To avoid the influence of random factors, each algorithm is independently run 10 times.

### 4.1. Real-World Engineering Optimization Problems

#### 4.1.1. Three-Bar Truss Design Problem

The three-bar truss design problem is a common nonlinear optimization problem in the field of civil engineering [40]. The optimization objective is to minimize the total volume of the three-bar truss. During the design process, buckling constraints, deflection constraints, and stress constraints must be imposed on each member. The objective function and constraint expressions are given as follows:

Minimize the following:

(23) $$f(x)=\left(2\sqrt{2}x_{1}+x_{2}\right)\cdot l$$

subject to

(24) $$\left\{\begin{array}{l} g_{1}(x)=\frac{\sqrt{2}x_{1}+x_{2}}{\sqrt{2}x_{1}^{2}+2x_{1}x_{2}}P-\sigma \leq 0\\ g_{2}(x)=\frac{x_{2}}{\sqrt{2}x_{1}^{2}+2x_{1}x_{2}}P-\sigma \leq 0\\ g_{3}(x)=\frac{1}{\sqrt{2}x_{2}+x_{1}}P-\sigma \leq 0 \end{array}\right.$$

with variable bound

(25) $$0\leq x_{1},x_{2}\leq 1$$

where the parameter *l* = 100 cm, *P* = 2 kN/cm^2^, and *σ* = 2 kN/cm^2^.

CMSCSO is applied to the three-bar truss optimization problem to optimize its two design variables and obtain the corresponding results. Table 15 presents the optimization results obtained by each algorithm.

According to the results, the optimal solution obtained by CMSCSO is *x*_1_ = 0.7887 and *x*_2_ = 0.4082, with the corresponding optimal objective value of 263.8958. Although the optimal values obtained by SCSO and the TOC are close to that of CMSCSO, CMSCSO has a smaller standard deviation. This indicates that CMSCSO has stronger convergence stability.

#### 4.1.2. Ten-Bar Truss Design Problem

The ten-bar truss is a classical benchmark problem for structural frequency optimization [41]. The main objective of this problem is to minimize the weight of the truss structure while satisfying multiple-order frequency constraints.

Minimize the following:

(26) $$f(\bar{x})=\sum_{i=1}^{10}L_{i}(x_{i})\rho_{i}A_{i}$$

subject to

(27) $$\left\{\begin{array}{l} g_{1}(\bar{x})=\frac{7}{\omega_{1}(\bar{x})}-1\leq 0\\ g_{2}(\bar{x})=\frac{15}{\omega_{2}(\bar{x})}-1\leq 0\\ g_{3}(\bar{x})=\frac{20}{\omega_{3}(\bar{x})}-1\leq 0 \end{array}\right.$$

with variable bound 6.45 × 10^−5^ ≤ *A_i_* ≤5 × 10^−3^, *i* = 1,2,…,10. *ω*_1_ ≥ 7 Hz, *ω*_2_ ≥ 15 Hz, *ω*_3_ ≥ 20 Hz.

Here, $\bar{x}$ = {*A*_1_, *A*_2_,…, *A*_10_}, *ρ* = 2770 kg/m^3^.

CMSCSO is used to perform the optimization test on this problem. The test results are shown in Table 16.

As shown in Table 16, CMSCSO ranks first in terms of the best value, average value, and standard deviation. The best value and average value obtained by the original SCSO are 528.6700 and 535.4371, respectively. CMSCSO further reduces the structural weight compared with the original SCSO. In contrast, the average values of the comparison algorithms, such as the BBO, FGO, CAOA, and ZOA, are significantly higher. This indicates that these algorithms tend to obtain inferior solutions when dealing with frequency constraints and multivariable coupling relationships.

#### 4.1.3. Weight Minimization of a Speed Reducer

The speed reducer design problem is a typical mechanical engineering optimization problem [42]. The optimization objective is to minimize the total weight of the speed reducer by comprehensively considering the gear weight and shaft deflection. This problem contains seven design variables, namely the gear parameters *b* (width), *m* (module), and *p* (number of teeth), as well as the bearing spans *l*_1_, *l*_2_ and shaft diameter *d*_1_, *d*_2_. By solving engineering constraints involved in this problem, the weight of the speed reducer is minimized as follows.

Minimize the following:

(28) $$\begin{array}{cc} f(\bar{x}) & =0.7854x_{2}^{2}x_{1}\left(14.9334x_{3}-43.0934+3.3333x_{3}^{2}\right)+0.7854\left(x_{5}x_{7}^{2}+x_{4}x_{6}^{2}\right)\\ & \;-1.508x_{1}\left(x_{7}^{2}+x_{6}^{2}\right)+7.477\left(x_{7}^{3}+x_{6}^{3}\right) \end{array}$$

subject to

(29) $$\left\{\begin{array}{l} g_{1}(\bar{x})=-x_{1}x_{2}^{2}x_{3}+27\leq 0\\ g_{2}(\bar{x})=-x_{1}x_{2}^{2}x_{3}^{2}+397.5\leq 0\\ g_{3}(\bar{x})=-x_{2}x_{6}^{4}x_{3}x_{4}^{-3}+1.93\leq 0\\ g_{4}(\bar{x})=-x_{2}x_{7}^{4}x_{3}x_{5}^{-3}+1.93\leq 0\\ g_{5}(\bar{x})=10x_{6}^{-3}\sqrt{16.91\times 10^{6}+{\left(745x_{4}x_{2}^{-1}x_{3}^{-1}\right)}^{2}}-1100\leq 0\\ g_{6}(\bar{x})=10x_{7}^{-3}\sqrt{157.5\times 10^{6}+{\left(745x_{5}x_{2}^{-1}x_{3}^{-1}\right)}^{2}}-850\leq 0\\ g_{7}(\bar{x})=x_{2}x_{3}-40\leq 0\\ g_{8}(\bar{x})=-x_{1}x_{2}^{-1}+5\leq 0\\ g_{9}(\bar{x})=x_{1}x_{2}^{-1}-12\leq 0\\ g_{10}(\bar{x})=1.5x_{6}-x_{4}+1.9\leq 0\\ g_{11}(\bar{x})=1.1x_{7}-x_{5}+1.9\leq 0 \end{array}\right.$$

with variable bound 2.6 < *x*_1_ < 3.6, 0.7 < *x*_2_ < 0.8, 17 < *x*_3_ < 28, 7.3 < *x*_4_, *x*_5_ < 8.3, 2.9 < *x*_6_ < 3.9, 5 < *x*_7_ < 5.5.

Based on the improved SCSO, this problem is optimized and compared with other comparison algorithms. The detailed results are shown in Table 17.

Table 17 shows that CMSCSO achieves the best value of 2998.9595, an average value of 3005.6112, and a standard deviation of 3.5180. These are the best results among all algorithms. It is worth noting that the FGO and ZOA show abnormally large average values and standard deviations on this problem. This indicates that these algorithms may generate infeasible search results under complex constraints. In contrast, CMSCSO can stably search for better design schemes within the constrained space, showing strong adaptability to engineering constraints.

#### 4.1.4. Reinforced Concrete Beam Design Problem

This case takes a simply supported beam with a span of 30 ft as the design object. The beam is subjected to a live load of 2000 lb and a dead load of 1000 lb. Based on the costs of concrete and steel, the objective is to minimize the total structural cost while ensuring safety. The compressive strength of concrete *σ_c_* is 5 ksi, and the yield stress of reinforcing steel *σ_y_* is 50 ksi. The cost of concrete is 0.02 USD/in^2^/linear ft, and the cost of steel is 1.0 USD/in^2^/linear ft.

According to ACI Building Code 318-77, the specified flexural strength must be satisfied:

(30) $$M_{u}=0.9A_{s}\sigma_{y}(0.8h)\left(1.0-0.59\frac{A_{s}\sigma_{y}}{0.8bh\sigma_{c}}\right)\geq 1.4M_{d}+1.7M_{l}$$

where the design variables are the reinforcement area *A_s_*, beam width *b*, and beam depth *h*. *M_u_*, *M_d_* = 1350 kip, and *M_l_* = 2700 kip denote the flexural strength of the beam, the dead load, and the live load, respectively. This optimization problem can be formulated as follows.

Minimize the following:

(31) $$f\left(X\right)\;=\;2.9x_{1}+0.6x_{2}x_{3}$$

subject to

(32) $$\left\{\begin{array}{l} g_{1}(X)=\frac{x_{2}}{x_{3}}-4\leq 0\\ g_{2}(X)=180+7.375\frac{x_{1}^{2}}{x_{3}}-x_{1}x_{2}\leq 0 \end{array}\right.$$

with variable bound

(33) $$\left\{\begin{array}{l} x_{1}\in \{6,6.16,6.32,6.6,7,7.11,7.2,7.8,7.9,8,8.4\}\\ x_{2}\in \{28,29,30,\ldots ,40\}\\ 5\leq x_{3}\leq 10 \end{array}\right.$$

For mixed discrete–continuous variables, a continuous relaxation and mapping strategy was adopted. The CMSCSO algorithm is used to optimize the three design variables of the reinforced concrete beam. The results are shown in Table 18.

As shown in Table 18, CMSCSO obtains the same best value as the TOC, which is the best result among all algorithms. However, the average value of CMSCSO is smaller than that of the TOC. This indicates that CMSCSO is more likely to remain near a better design region over multiple independent runs. Although the standard deviation of SCSO is smaller than that of CMSCSO, SCSO fails to converge to a better position. Overall, CMSCSO demonstrates high optimization accuracy and good average performance in the reinforced concrete beam design problem.

#### 4.1.5. Tubular Column Design Problem

The objective of the tubular column design problem is to design a tubular compression column with a uniform cross section [43]. The manufacturing cost is minimized while satisfying the axial compressive load-bearing requirements. The optimization model of this problem is given as follows.

Minimize the following:

(34) $$f\left(X\right)\;=\;9.8x_{1}x_{2}+2x_{1}$$

subject to

(35) $$\left\{\begin{array}{l} g_{1}(X)=\frac{P}{\pi x_{1}x_{2}\sigma_{y}}-1\leq 0\\ g_{2}(X)=\frac{8PL^{2}}{\pi^{3}Ex_{1}x_{2}\left(x_{1}^{2}+x_{2}^{2}\right)}-1\leq 0\\ g_{3}(X)=\frac{2.0}{x_{1}}-1\leq 0\\ g_{4}(X)=\frac{x_{1}}{14}-1\leq 0\\ g_{5}(X)=\frac{0.2}{x_{2}}-1\leq 0\\ g_{6}(X)=\frac{x_{2}}{8}-1\leq 0 \end{array}\right.$$

with variable bound 2 ≤ *x*_1_ ≤ 14, 0.2 ≤ *x*_2_ ≤ 0.8 and the parameter *σ_y_* = 500 kgf/cm^2^ and E = 0.85 × 10^6^ kgf/cm^2^.

The test results of the tubular column design problem are shown in Table 19.

As shown in Table 19, CMSCSO obtains the design results of *x*_1_ = 6.54744 and *x*_2_ = 0.20870, with the corresponding best value of 26.4860, average value of 26.4860, and standard deviation of 7.72 × 10^−6^. Although the TOC achieves the same best value as CMSCSO, its standard deviation is inferior to that of CMSCSO. Therefore, CMSCSO can not only obtain the optimal objective value in this problem but also produce almost no fluctuation over multiple independent runs, demonstrating extremely strong stability.

#### 4.1.6. Compression Spring Design Problem

The compression spring optimization design problem aims to minimize the spring weight [44]. It includes four constraints, namely the deflection constraint, shear stress constraint, frequency constraint, and outer diameter constraint. The design variables include the mean coil diameter, wire diameter, and number of active coils. The specific mathematical model is given as follows.

Minimize the following:

(36) $$\min\;f(x)=(N+2)Dd^{2}$$

subject to

(37) $$\left\{\begin{array}{l} g_{1}(x)=1-\frac{D^{3}N}{71785d^{4}}\leq 0\\ g_{2}(x)=\frac{4D^{2}-dD}{12566\left(Dd^{3}-d^{4}\right)}+\frac{1}{5108d^{2}}-1\leq 0\\ g_{3}(x)=1-\frac{140.45d}{D^{2}N}\leq 0\\ g_{4}(x)=\frac{D+d}{1.5}-1\leq 0 \end{array}\right.$$

with variable bound 0.05 ≤ *x*_1_ ≤ 2, 0.25 ≤ *x*_2_ ≤ 1.3, 2 ≤ *x*_3_ ≤ 15.

Table 20 presents the optimization results of this problem. As shown in Table 15, CMSCSO, SCSO, and the TOC all obtain the optimal solution for the compression spring design problem. However, CMSCSO has a smaller standard deviation. Therefore, CMSCSO shows strong competitiveness in this problem.

Overall, CMSCSO demonstrates good practical applicability. The proposed improvement strategies not only enhance the performance of the algorithm on standard benchmark functions but also improve its constraint adaptability, optimization accuracy, and operational reliability in real-world engineering problems. In particular, for multivariable optimization and complex constrained problems, CMSCSO can obtain high-quality solutions more stably.

### 4.2. WSN Coverage Optimization

In wireless sensor networks (WSNs), the coverage rate of node deployment is an important indicator of installation cost and monitoring quality. A higher coverage rate indicates better sensor network performance. Therefore, the reasonable deployment of sensor nodes is of great significance for improving monitoring coverage.

#### 4.2.1. 2D WSN Coverage Optimization

To optimize the deployment of the target coverage area, the coverage area and the WSN should first be mathematically modeled. Assume that the target coverage area is *S*, in which *n* sensor nodes are deployed. Each sensor node adopts a Boolean sensing model. First, *S* is discretized into the *e* × *f* subregion. The node of each subregion is denoted as *s_i_* = (*x_i_*,*y_i_*), and each sensor node is denoted as *c_i_* = (*x_i_*,*y_i_*), i = 1, 2,… *n*. In this study, differences between sensors are ignored. All sensors are assumed to have the same sensing radius *r_d_* and communication radius *r_t_*, where *r_t_* ≥ 2*rd*. This assumption is used to focus on the effect of the deployment optimization algorithm. If different sensing ranges, communication abilities, or measurement errors were considered at the same time, the performance differences could be affected by both the optimization method and the sensor settings. Therefore, ignoring the differences between sensors provides a controlled basis for comparing CMSCSO with the other algorithms under the same objective function.

The Euclidean distance between a sensor and a node, as well as the sensing probability, can be calculated as follows:

(38) $$d(s_{i},c_{i})=\sqrt{{\left(s_{x_{i}}-c_{x_{i}}\right)}^{2}+{\left(s_{x_{j}}-c_{x_{j}}\right)}^{2}}$$

(39) $$p(s_{i},c_{i})=\left\{\begin{array}{ll} 1 & d(s_{i},c_{i})\leq r_{d}\\ 0 & d(s_{i},c_{i})>r_{d} \end{array}\right.$$

The final WSN coverage rate is defined as the ratio of the total sensing area covered by all sensor nodes to the target area *S*. A larger value indicates a higher coverage rate. Therefore, Equation (40) is used as the objective function of the WSN node coverage optimization problem.

(40) $$p=\frac{\sum_{i=1}^{e\times f}\left(1-\prod_{j=1}^{n}\left(1-p(s_{i},c_{i})\right)\right)}{S}$$

**Figure 7:**
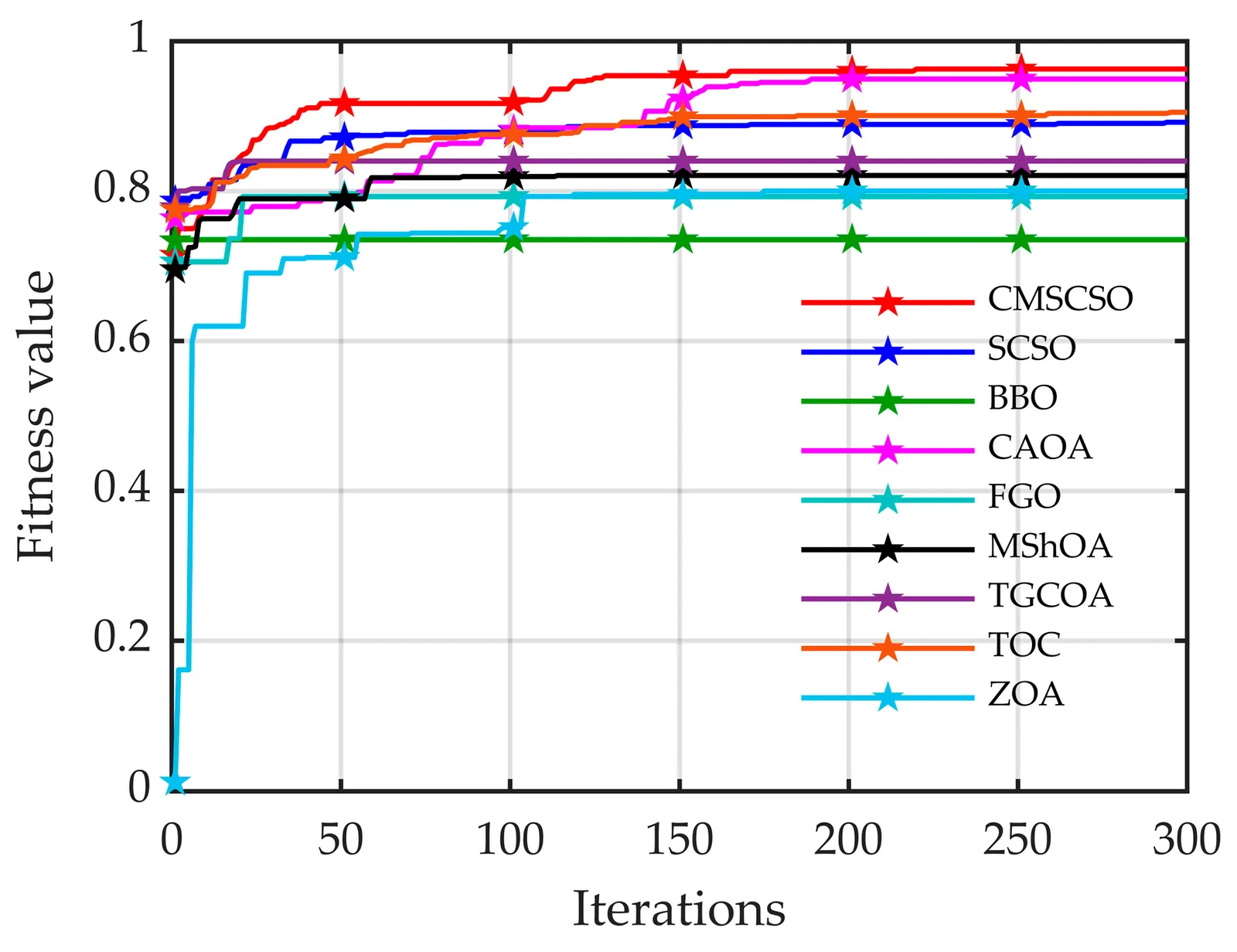
Iterative curves for 2D WSN coverage.

**Figure 8:**
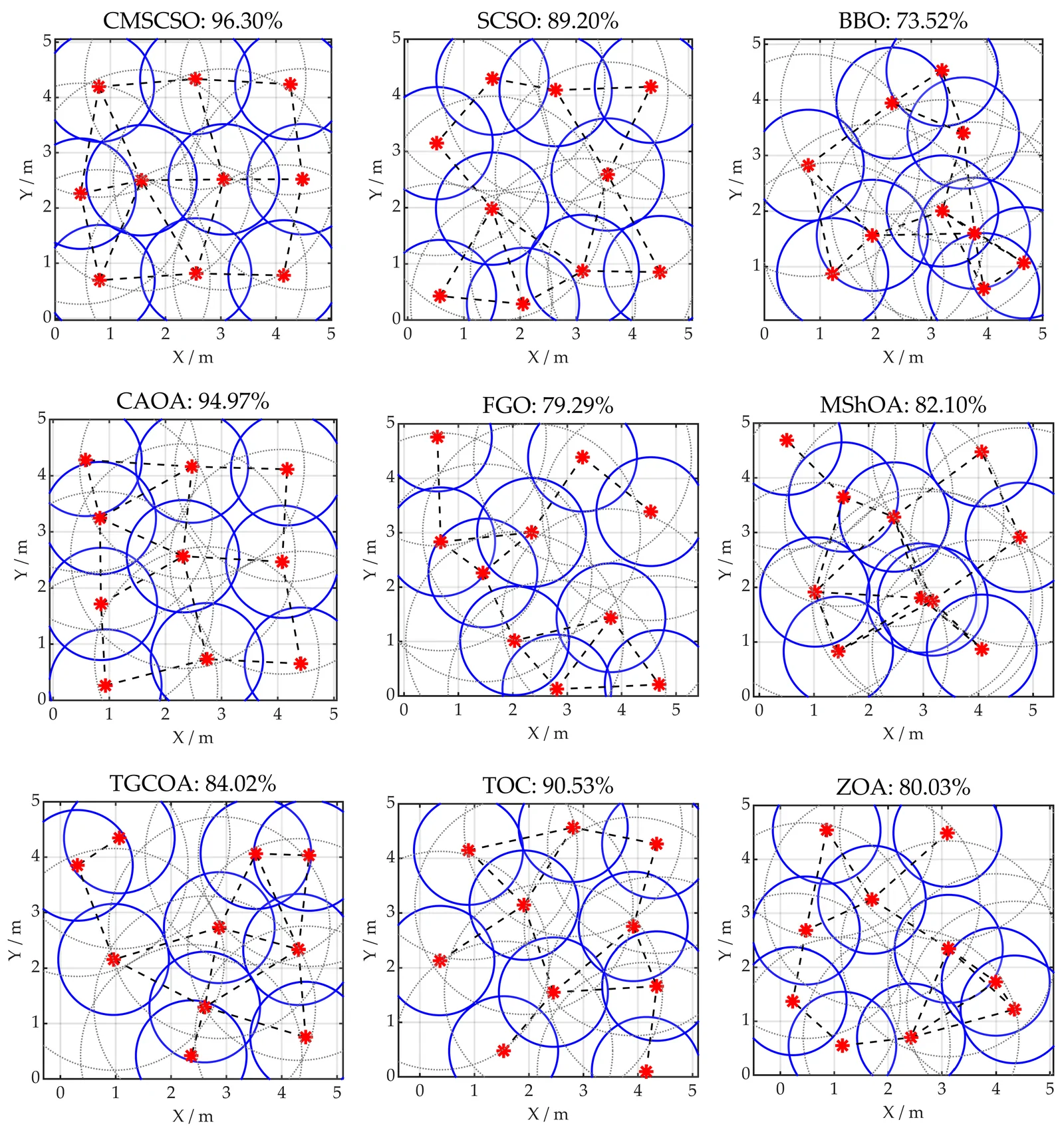
Optimized node deployment map for a 2D WSN (* denote the deployed sensor nodes, the blue circles correspond to sensing radius, the gray dotted circles correspond to communication radius and the dashed lines indicate the communication links established between mutually reachable sensor nodes).

The proposed CMSCSO algorithm and the comparison algorithms are further analyzed through simulations on the WSN coverage optimization problem. The parameters of the 2D WSN problem are set as follows: the coverage area is 5 m × 5 m, the number of WSN nodes is 10, the sensing radius of each node is 1 m, and the communication radius is 2 m. The population size of each algorithm is 20, and the maximum number of iterations is 300. Each algorithm is independently run 10 times, and the best result is recorded. The optimization results are shown in Table 21. The convergence curves and node deployment results are shown in Figure 7 and Figure 8, respectively.

As shown in Table 21, CMSCSO achieves a coverage rate of 96.30%, showing the best performance among all algorithms. Compared with the original SCSO, the coverage rate of CMSCSO is improved by 7.10%. This indicates that the proposed strategies can effectively enhance the optimization capability of the algorithm in the 2D WSN node deployment problem. Compared with the other algorithms, CMSCSO also maintains a clear advantage. This demonstrates that CMSCSO can achieve more sufficient area coverage with a limited number of nodes.

As shown in the training process in Figure 7, CMSCSO exhibits a fast convergence speed in the early stage of iteration and maintains stable optimization capability in the later stage, eventually achieving a good convergence result. Algorithms such as the BBO and ZOA fall into local optima too early and fail to escape effectively, resulting in low convergence accuracy. Figure 8 presents the node deployment results obtained by each algorithm. The node distribution optimized by CMSCSO is more uniform, with relatively smaller overlapping coverage areas and uncovered areas between nodes. In contrast, the node distributions obtained by the BBO, FGO, ZOA, and other algorithms show a certain degree of aggregation, leading to lower overall coverage rates.

Based on the above experimental results, CMSCSO demonstrates better coverage performance and a superior node deployment scheme in the 2D WSN coverage optimization problem.

#### 4.2.2. 3D WSN Coverage Optimization

In a three-dimensional coverage area, the coverage region of each sensor node is a sphere determined by a certain radius. Assume that *N* sensor nodes are randomly deployed in a three-dimensional monitoring area, forming the node set *S* = {*s*_1_, *s*_2_, …, *s*_n_}. The coordinate of the *i*-th node is denoted as *s_i_*(*x_i_*,*y_i_*,*z_i_*). In the three-dimensional region, the distance between the sensor node *l_v_*(*x_v_*, *y_v_*, *z_v_*) and a region node is calculated as follows:

(41) $$d(s_{i},l_{v})=\sqrt{{\left(x_{i}-x_{v}\right)}^{2}+{\left(y_{i}-y_{v}\right)}^{2}+{\left(z_{i}-z_{v}\right)}^{2}}$$

The three-dimensional sensor model also adopts the Boolean model. Its sensing probability is expressed as follows:

(42) $$p(s_{i},l_{v})=\left\{\begin{array}{ll} 1 & d(s_{i},l_{v})\leq r_{d}\\ 0 & d(s_{i},l_{v})>r_{d} \end{array}\right.$$

where *r_d_* denotes the communication radius of the node. The final 3D WSN coverage rate is then defined as follows:

(43) $$p=\frac{\sum_{i=1}^{k}R_{p}(k_{alli},l_{v})}{k}$$

(44) $$R_{p}(k_{alli},l_{v})=1-\prod_{i=1}^{k}\left(1-p(s_{i},l_{v})\right)$$

where *k* denotes the number of nodes to be tested, *k_all_* denotes all nodes to be tested in the monitoring area, and *R_p_* denotes the joint sensing probability.

**Figure 9:**
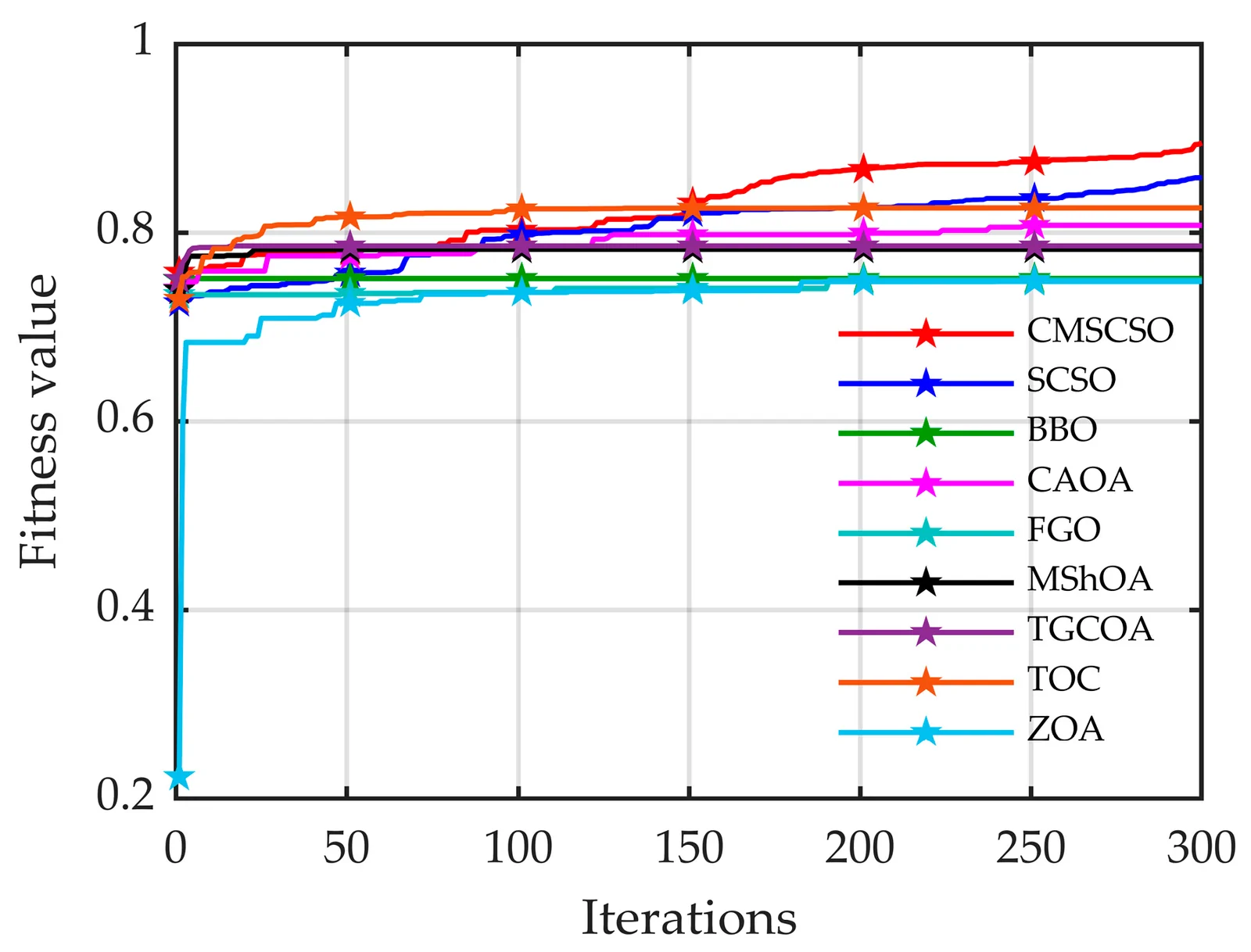
Iterative curves for 3D WSN coverage.

**Figure 10:**
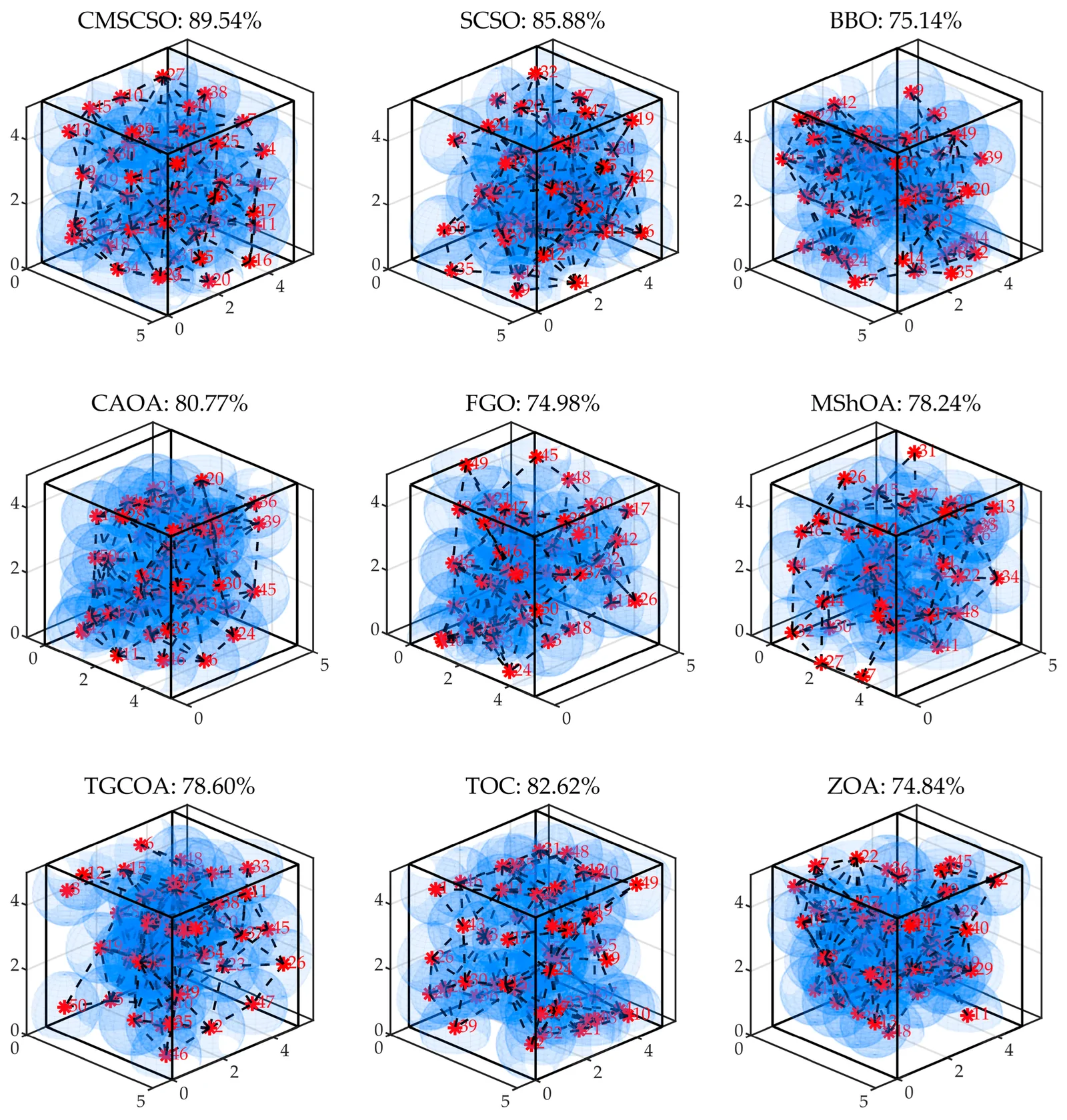
Optimized node deployment map for a 3D WSN (* denote sensor nodes, the translucent blue spheres represent the sensing region and the dashed lines indicate communication links between nodes).

The parameters of the 3D WSN problem are set as follows: the coverage area is 5 m × 5 m × 5 m, and the number of WSN nodes is 40. The remaining parameters are consistent with those of the 2D WSN problem. The optimization results are shown in Table 22. The convergence curves and node deployment results are shown in Figure 9 and Figure 10, respectively.

As shown in Table 22, CMSCSO still achieves the best result in the 3D WSN coverage optimization scenario, obtaining the highest coverage rate. Compared with SCSO, the coverage rate is improved by 3.66%. The improvement is more significant when compared with algorithms such as the BBO, FGO, and ZOA. The experimental results indicate that CMSCSO can still maintain strong global search capability and node deployment optimization ability when the search dimension is extended from a two-dimensional space to a three-dimensional space.

As shown by the convergence curves in Figure 9, CMSCSO can rapidly improve the fitness value in the early stage of iteration and continue to maintain a slow upward trend in the middle and later stages. This indicates that CMSCSO can further improve node positions based on the known high-quality solutions, without falling into local optimum traps. Algorithms such as the BBO, FGO, and TGCOA remain in a stable state for a long time in the later stage of iteration, with only slight improvements in the coverage rate. Therefore, these algorithms are easily affected by the increase in spatial dimensionality in the 3D WSN optimization problem, resulting in insufficient exploitation capability in the later stage. Although SCSO and the TOC show relatively good performance, their final coverage rates are still lower than that of CMSCSO. This indicates that the proposed multi-strategy collaborative mechanism can further improve the quality of three-dimensional node deployment.

As shown in the three-dimensional node deployment results in Figure 10, the nodes obtained by CMSCSO are distributed relatively uniformly within the spatial region, and the sensing spheres can sufficiently fill the three-dimensional space. The coverage rates of the FGO and ZOA are lower than 75%, indicating that their node distributions cannot fully meet the coverage requirements of the three-dimensional space.

Compared with the two-dimensional problem, the 3D WSN coverage optimization problem has a higher search dimension and more complex spatial constraint relationships. Therefore, it places higher requirements on the global search capability and local exploitation capability of the algorithm. CMSCSO achieves the best results in both dimensional scenarios. This indicates that the proposed strategies are not only applicable to two-dimensional planar node deployment but can also be effectively extended to three-dimensional space, demonstrating good robustness and engineering application potential.

### 4.3. Parameter Identification of Photovoltaic Models

To accurately characterize the output behavior of photovoltaic modules and improve the prediction accuracy of maximum power point tracking, the model parameters must be identified using the photovoltaic cell datasheet specifications or measured data. Two commonly used photovoltaic models are the single-diode model (SDM) and the double-diode model (DDM) [45].

#### 4.3.1. Single-Diode Model

**Figure 11:**
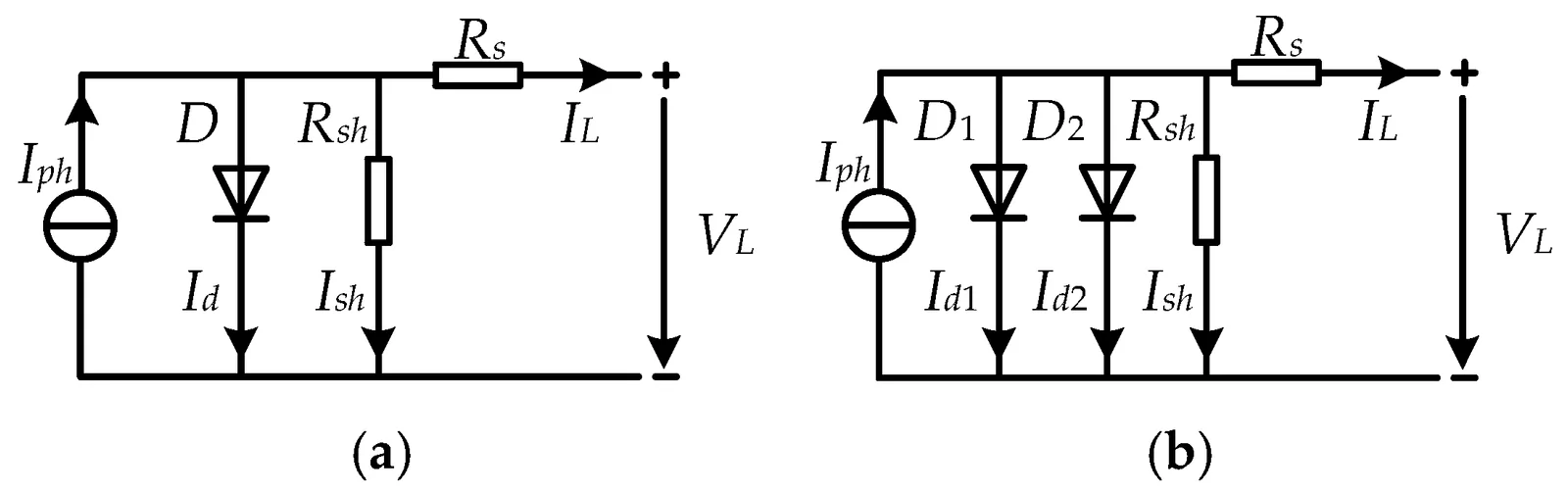
Equivalent circuits of photovoltaic models. (**a**) SDM. (**b**) DDM.

As shown in Figure 11a, the SDM consists of a current source, a diode, a series resistance, and a shunt resistance. It represents the internal equivalent circuit of a photovoltaic cell using a relatively small number of parameters and is commonly employed to describe an individual photovoltaic cell. Based on the equivalent circuit shown in Figure 11a, the output current *I_L_* of the SDM can be derived from Kirchhoff’s voltage law (KVL) and the Shockley diode equation as follows:

(45) $$I_{L}=I_{ph}-I_{0}\left[e^{\frac{q(V_{L}+R_{s}I_{L})}{nkT}}-1\right]-\frac{V_{L}+R_{s}I_{L}}{R_{sh}}$$

where *I_ph_* denotes the photocurrent, *I_0_* is the reverse saturation current of the diode, and *V_L_* represents the output voltage of the photovoltaic cell. *R_s_* and *R_sh_* denote the equivalent series resistance and shunt resistance, respectively, *q* is the elementary charge (q = 1.602 × 10^−19^ C), *n* is the diode ideality factor, *k* is the Boltzmann constant (k = 1.381 × 10^−23^ J/K), and *T* denotes the operating temperature.

#### 4.3.2. Double-Diode Model

As shown in Figure 11b, the DDM introduces an additional diode into the SDM to represent the current loss caused by carrier recombination. Compared with the SDM, the DDM provides a more accurate representation of the internal characteristics of an actual photovoltaic cell. According to KVL, the output current *I_L_* of the DD model can be expressed as follows:

(46) $$I_{L}==I_{ph}-I_{d1}\left[\exp\left(\frac{q(I_{L}R_{s}+V_{L})}{n_{1}kT}\right)-1\right]-I_{d2}\left[\exp\left(\frac{q(I_{L}R_{s}+V_{L})}{n_{2}kT}\right)-1\right]-\frac{V_{L}+I_{L}R_{s}}{R_{sh}}$$

where *I_d_*_1_ and *I_d_*_2_ denote the reverse saturation currents of diodes *D*_1_ and *D*_2_, respectively, and *n*_1_ and *n*_2_ are the ideality factors of *D*_1_ and *D*_2_, respectively. The DDM requires the identification of seven parameters. Although this model can provide higher accuracy, the additional parameters increase the difficulty of parameter identification.

#### 4.3.3. Parameter Identification Results and Analysis

In parameter identification problems, the root mean square error (RMSE) is commonly used to quantify the deviation between predicted and actual values. Based on the requirements of photovoltaic model parameter identification, the optimization objective is formulated as follows:

(47) $$RMSE(x)=\sqrt{\frac{1}{M}\sum_{i=1}^{M}f{\left(V_{L},I_{L},X\right)}^{2}}$$

where *M* is the number of measurement points and *X* denotes the vector of parameters to be identified. In this study, the measured *I*-*V* data of an RTC photovoltaic cell are used as the test benchmark [46]. The decision variables and their corresponding constraints for the two models are listed in Table 23.

**Figure 12:**
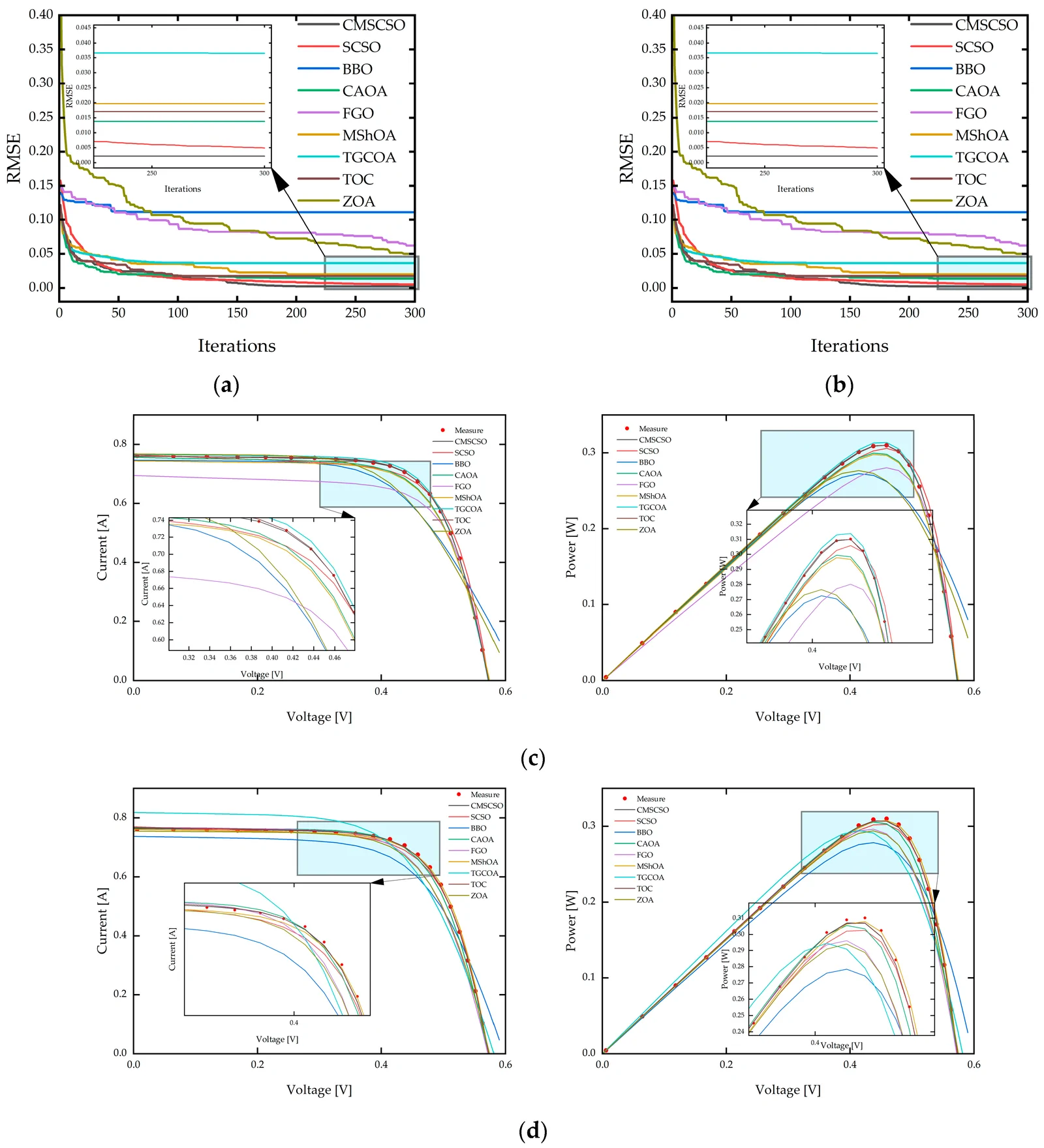
Parameter identification results of photovoltaic models: (**a**) average convergence curve of SDM; (**b**) average convergence curve of DDM; (**c**) characterization curves of SDM (left is *I*-*V* curve, right is *P*-*W* curve); (**d**) characterization curves of DDM (left is *I*-*V* curve, right is *P*-*W* curve).

The experimental settings were identical to those used for the engineering optimization problems described above. Figure 12 presents the convergence curves and characteristic curves obtained by different algorithms for the SDM and DDM. Table 24 and Table 25 report the parameter identification results for the two models, respectively.

Figure 12a and Figure 12b present the average convergence curves of the compared algorithms for the SDM and DDM, respectively. CMSCSO rapidly reduces the objective function value during the early iterations. It continues to identify improved solutions throughout the subsequent search process and ultimately converges to the lowest RMSE level. According to the results presented in Table 24, CMSCSO achieves the best minimum RMSE, mean RMSE, and RMSE standard deviation for the SDM. These results demonstrate its high accuracy and robustness in parameter identification. As shown by the fitting results in Figure 12c, the photovoltaic characteristic curves obtained by CMSCSO are highly consistent with the measured data points. In particular, the output characteristics of the photovoltaic cell are accurately reproduced near the knee point of the curves.

Table 25 presents the parameter identification results for the DDM. Owing to the increased number of parameters to be identified, the DDM involves a higher-dimensional and more challenging search problem than the SDM. Therefore, greater optimization capability is required. Nevertheless, CMSCSO still achieves the best parameter identification performance. As shown in Figure 12d, CMSCSO accurately reconstructs the output characteristics of the photovoltaic cell and produces the smallest deviation from the measured values among all the compared algorithms.

### 4.4. Quantitative Summary of Engineering Optimization Results

The engineering applications have demonstrated the applicability of the proposed method. To further quantify the practical significance of CMSCSO, this subsection calculates its performance improvements over the standard SCSO and the other comparison algorithms based on the mean results obtained from multiple independent runs on the engineering problems described above. Mean values, rather than only the best values, were selected as the evaluation criterion because they provide a more reliable representation of the overall performance of stochastic optimization algorithms across repeated trials. Table 26 presents the percentage improvements achieved by CMSCSO relative to the original SCSO and the other competing algorithms.

As shown in Table 26, CMSCSO achieved better average optimization results than the standard SCSO on all six classical engineering design problems. The most substantial improvements were observed for the compression spring and ten-bar truss problems, for which CMSCSO reduced the average objective values by 1.4925% and 0.6297%, respectively. The improvement for the speed reducer problem was only 0.0804%, whereas the gains over SCSO were marginal for the three-bar truss, reinforced concrete beam, and tubular column problems. Most classical engineering design problems have relatively low-dimensional decision spaces. Consequently, several comparison algorithms can converge to the same or neighboring high-quality feasible regions, leaving limited room for further improvement in the objective value. Although the reductions in objective value were small for some problems, CMSCSO not only achieved lower average objective values on most classical optimization problems but also reduced the variability among multiple independent runs. It should also be noted that the FGO and ZOA generated infeasible solutions in repeated runs on several constrained design problems. This phenomenon further confirms the importance of consistently satisfying constraints in engineering optimization. Algorithm performance should not be evaluated solely on the basis of a low objective value obtained from a single run.

CMSCSO exhibited more pronounced practical optimization performance in the wireless sensor network (WSN) cases. Compared with the standard SCSO, CMSCSO increased the relative coverage rate by 7.9596% for the two-dimensional WSN and by 4.2618% for the three-dimensional WSN. In absolute terms, these improvements corresponded to increases of 7.10 and 3.66 percentage points, respectively. Compared with the other algorithms, CMSCSO improved the coverage rate by 30.9848% over the BBO in the two-dimensional scenario and by 19.6419% over the ZOA in the three-dimensional scenario.

The most substantial performance improvements were obtained for photovoltaic model parameter identification. For the SDM, CMSCSO reduced the RMSE by 54.0000% relative to the standard SCSO. Compared with the other algorithms, the RMSE reductions ranged from 83.2117% to 97.9279%. For the DDM, CMSCSO reduced the RMSE by 35.4167% relative to SCSO and by 73.2759–96.9903% relative to the other comparison algorithms. The CAOA was the best-performing comparison algorithm for both photovoltaic parameter identification problems. Nevertheless, CMSCSO reduced the RMSE by 83.2117% relative to the CAOA for the SDM and by 73.2759% for the DDM. These results demonstrate that CMSCSO has a particularly strong advantage in nonlinear parameter identification problems, where even small deviations in the identified parameters can cause large discrepancies between the measured and reconstructed current–voltage characteristic curves.

Overall, the engineering applicability of CMSCSO varies with the type of optimization problem. Its advantages are reflected not only in the reduction of objective values but also in its ability to consistently produce high-quality feasible solutions across diverse engineering domains.

## 5. Conclusions

To address the insufficient search accuracy of standard SCSO in high-dimensional, nonlinear, and complex optimization problems, this paper proposes a CMSCSO algorithm based on multi-strategy collaborative search. By introducing the good point set initialization strategy, worst-value opposition-based learning, the prey impatience coefficient, and the random reuse strategy into standard SCSO, the proposed algorithm effectively improves the global search and local exploitation capabilities without changing the time complexity and space complexity of standard SCSO. As a result, the optimization accuracy is effectively enhanced.

According to the test results on the CEC2017 and CEC2022 benchmark test suites, CMSCSO outperforms SCSO and recent state-of-the-art algorithms in terms of solution accuracy and stability. The nonparametric experimental results of the Wilcoxon rank-sum test and Friedman ranking test demonstrate that the performance of CMSCSO is statistically significant. CMSCSO ranks first in both types of nonparametric tests, which further verifies its superiority. The ablation experimental results confirm that all four improvement strategies have positive effects on algorithm performance, and the comprehensive optimization effect of multi-strategy collaboration is better than that of any single strategy. These results fully demonstrate the rationality and effectiveness of the collaborative search framework constructed in this paper. In real-world engineering optimization problems, six classical optimization problems from different fields, WSN coverage optimization problems under two different dimensional scales, and photovoltaic model parameter identification problems are selected to verify the performance of the algorithm. The test results show that CMSCSO can stably search for high-quality solutions satisfying the constraints in the six classical engineering problems, while achieving better stability. In both 2D and 3D WSN coverage optimization problems, CMSCSO obtains the highest regional coverage rate. The optimized node distribution is more uniform, and the overlapping coverage area is smaller. CMSCSO also achieved the best performance in the parameter identification problem.

The above results fully indicate that CMSCSO not only shows outstanding performance in standard benchmark tests but also has good applicability and reliability in real-world complex engineering problems. It can provide an efficient and stable solution for complex optimization problems in mechanical engineering, structural design, sensor deployment optimization, and related fields.

Although CMSCSO achieves good optimization results, there is still room for further improvement:

1. The current algorithm mainly focuses on single-objective optimization scenarios. In the future, it can be extended to discrete, multi-objective, and dynamic optimization problems.

2. The engineering validation conducted in this study was limited to six engineering optimization problems, WSN coverage optimization problems in two different dimensional settings, and photovoltaic model parameter identification problems. Future studies can further explore its application value in more engineering fields, such as path planning, energy scheduling, and image segmentation.

3. A new adaptive regulation mechanism can be designed to dynamically adjust the contribution weights of different strategies, thereby achieving a more refined balance between global search and local exploitation.

## Figures and Tables

**Table 1:** CMSCSO improvement strategy.

Improvement Stage	Adopted Strategy	Effect
Population Initialization	Good Point Set	Improve the uniformity of the initial population
Exploration and Exploitation Phase	Random Reuse Strategy + Prey Impatience Coefficient	Enhance global search capability and improve local search accuracy
Local Optimum Escaping	Worst-Individual Opposition-Based Learning	Prevent premature convergence

**Table 2:** Algorithm parameter settings.

Algorithm	References	Parameter	Value
BBO	Ouyang et al., 2025 [21]	*r*_6_	𝒩(0,1)
CAOA	Xu et al., 2025 [22]	*α*	0.5
		*β*	0.1
		*γ*	0.1
		*δ*	1 × 10^−4^
		*E*_0_	100
FGO	Abdel-Basset al., 2025 [23]	*M*	0.6
		*E_p_*	0.7
		*R*	0.9
TGCOA	Gámez et al., 2025 [25]	*T_l_*	10
		*T_u_*	40
		*k*	0.03
		*α*	50
		*b*	10
TOC	Braik et al., 2025 [26]	*b_r_*	1 × 10^5^
		*χ*	4.1
		*Ω*	7.29 × 10^−5^
MshOA	Sánchez et al., 2025 [24]	*D*	random number in [−1, 1]
ZOA	Fatoorchi et al., 2026 [27]	*ω*	0.1
		*μ*	4
SCSO	Seyyedabbasi et al., 2023 [28]	*S_M_*	2
CMSCSO		*P_min_*	0.2
		*P_max_*	0.8

**Table 3:** Test results for the dim = 30 CEC2017 test suite (the optimal results are shown in bold).

Func	Metrics	CMSCSO	SCSO	BBO	CAOA	FGO	MShOA	TGCOA	TOC	ZOA
F1	min	**2.48 × 10^4^**	3.20 × 10^9^	4.47 × 10^0^	1.02 × 10^0^	3.09 × 10^10^	1.98 × 10^10^	3.88 × 10^10^	1.28 × 10^9^	2.34 × 10^10^
	std	**7.77 × 10^5^**	2.72 × 10^9^	5.77 × 10^9^	1.58 × 10^9^	8.21 × 10^9^	1.33 × 10^10^	8.25 × 10^9^	3.70 × 10^9^	9.70 × 10^9^
	avg	**6.59 × 10^5^**	7.05 × 10^9^	6.28 × 10^10^	1.28 × 10^10^	4.48 × 10^10^	4.86 × 10^10^	5.67 × 10^10^	7.06 × 10^9^	3.52 × 10^10^
F3	min	**2.49 × 10^4^**	3.90 × 10^4^	6.65 × 10^4^	3.84 × 10^4^	1.19 × 10^5^	7.13 × 10^4^	7.71 × 10^4^	3.39 × 10^4^	1.01 × 10^5^
	std	6.74 × 10^3^	1.01 × 10^4^	**5.27 × 10^3^**	6.51 × 10^3^	7.71 × 10^4^	9.06 × 10^3^	3.32 × 10^4^	1.12 × 10^5^	4.30 × 10^4^
	avg	**3.36 × 10^4^**	5.90 × 10^4^	8.46 × 10^4^	5.34 × 10^4^	2.44 × 10^5^	9.06 × 10^4^	1.15 × 10^5^	1.35 × 10^5^	1.91 × 10^5^
F4	min	**4.77 × 10^2^**	5.62 × 10^2^	9.35 × 10^3^	2.30 × 10^3^	4.54 × 10^3^	5.40 × 10^3^	1.09 × 10^4^	8.23 × 10^2^	4.31 × 10^3^
	std	**2.88 × 10^1^**	6.20 × 10^2^	2.77 × 10^3^	3.35 × 10^2^	2.27 × 10^3^	5.98 × 10^3^	3.93 × 10^3^	8.28 × 10^2^	3.40 × 10^3^
	avg	**5.36 × 10^2^**	1.06 × 10^3^	1.40 × 10^4^	2.94 × 10^3^	9.65 × 10^3^	1.59 × 10^4^	1.69 × 10^4^	1.70 × 10^3^	9.39 × 10^3^
F5	min	**6.15 × 10^2^**	6.92 × 10^2^	8.96 × 10^2^	6.46 × 10^2^	7.96 × 10^2^	8.32 × 10^2^	8.84 × 10^2^	7.10 × 10^2^	8.10 × 10^2^
	std	4.50 × 10^1^	3.92 × 10^1^	**2.14 × 10^1^**	3.72 × 10^1^	4.10 × 10^1^	4.39 × 10^1^	4.69 × 10^1^	5.42 × 10^1^	3.08 × 10^1^
	avg	**6.94 × 10^2^**	7.60 × 10^2^	9.46 × 10^2^	7.21 × 10^2^	9.12 × 10^2^	9.19 × 10^2^	9.71 × 10^2^	8.04 × 10^2^	8.95 × 10^2^
F6	min	**6.36 × 10^2^**	6.47 × 10^2^	6.83 × 10^2^	6.50 × 10^2^	6.63 × 10^2^	6.70 × 10^2^	6.80 × 10^2^	6.41 × 10^2^	6.61 × 10^2^
	std	8.01 × 10^0^	8.67 × 10^0^	**5.50 × 10^0^**	7.02 × 10^0^	8.70 × 10^0^	1.18 × 10^1^	1.11 × 10^1^	1.38 × 10^1^	1.00 × 10^1^
	avg	**6.48 × 10^2^**	6.64 × 10^2^	6.98 × 10^2^	6.59 × 10^2^	6.84 × 10^2^	6.90 × 10^2^	7.01 × 10^2^	6.61 × 10^2^	6.83 × 10^2^
F7	min	**8.85 × 10^2^**	9.79 × 10^2^	1.40 × 10^3^	9.45 × 10^2^	1.28 × 10^3^	1.32 × 10^3^	1.28 × 10^3^	1.03 × 10^3^	1.33 × 10^3^
	std	8.41 × 10^1^	8.57 × 10^1^	**2.86 × 10^1^**	6.44 × 10^1^	1.61 × 10^2^	7.30 × 10^1^	7.23 × 10^1^	1.02 × 10^2^	9.81 × 10^1^
	avg	**1.03 × 10^3^**	1.16 × 10^3^	1.45 × 10^3^	1.06 × 10^3^	1.67 × 10^3^	1.45 × 10^3^	1.47 × 10^3^	1.25 × 10^3^	1.51 × 10^3^
F8	min	**8.89 × 10^2^**	9.36 × 10^2^	1.12 × 10^3^	9.04 × 10^2^	1.15 × 10^3^	1.09 × 10^3^	1.08 × 10^3^	9.42 × 10^2^	1.06 × 10^3^
	std	3.50 × 10^1^	3.58 × 10^1^	**1.64 × 10^1^**	3.30 × 10^1^	2.35 × 10^1^	3.29 × 10^1^	3.40 × 10^1^	4.85 × 10^1^	3.22 × 10^1^
	avg	**9.55 × 10^2^**	1.01 × 10^3^	1.17 × 10^3^	9.58 × 10^2^	1.20 × 10^3^	1.14 × 10^3^	1.16 × 10^3^	1.06 × 10^3^	1.15 × 10^3^
F9	min	2.28 × 10^3^	4.50 × 10^3^	1.12 × 10^4^	2.85 × 10^3^	1.11 × 10^4^	6.25 × 10^3^	9.44 × 10^3^	**2.06 × 10^3^**	8.09 × 10^3^
	std	1.07 × 10^3^	1.27 × 10^3^	**8.27 × 10^2^**	1.38 × 10^3^	3.09 × 10^3^	2.06 × 10^3^	5.73 × 10^3^	2.06 × 10^3^	2.83 × 10^3^
	avg	**4.51 × 10^3^**	6.59 × 10^3^	1.29 × 10^4^	5.55 × 10^3^	1.70 × 10^4^	1.17 × 10^4^	1.46 × 10^4^	5.78 × 10^3^	1.27 × 10^4^
F10	min	4.22 × 10^3^	4.70 × 10^3^	8.36 × 10^3^	**3.79 × 10^3^**	8.46 × 10^3^	7.90 × 10^3^	8.39 × 10^3^	5.83 × 10^3^	6.97 × 10^3^
	std	5.33 × 10^2^	6.43 × 10^2^	**3.73 × 10^2^**	1.15 × 10^3^	4.50 × 10^2^	5.95 × 10^2^	4.69 × 10^2^	8.85 × 10^2^	7.28 × 10^2^
	avg	**5.11 × 10^3^**	6.09 × 10^3^	9.21 × 10^3^	6.44 × 10^3^	9.75 × 10^3^	8.83 × 10^3^	9.22 × 10^3^	7.58 × 10^3^	9.00 × 10^3^
F11	min	**1.25 × 10^3^**	1.84 × 10^3^	1.27 × 10^4^	2.39 × 10^3^	1.64 × 10^4^	7.46 × 10^3^	8.58 × 10^3^	1.74 × 10^3^	1.26 × 10^4^
	std	**1.07 × 10^2^**	1.42 × 10^3^	1.04 × 10^4^	4.88 × 10^2^	7.95 × 10^3^	5.42 × 10^3^	7.39 × 10^3^	6.57 × 10^3^	5.69 × 10^3^
	avg	**1.44 × 10^3^**	4.20 × 10^3^	2.41 × 10^4^	3.15 × 10^3^	2.78 × 10^4^	1.52 × 10^4^	2.06 × 10^4^	6.10 × 10^3^	2.22 × 10^4^
F12	min	**3.83 × 10^6^**	4.29 × 10^7^	6.54 × 10^9^	1.13 × 10^9^	2.83 × 10^9^	2.41 × 10^9^	4.06 × 10^9^	6.76 × 10^7^	2.15 × 10^9^
	std	**1.46 × 10^7^**	5.31 × 10^8^	3.31 × 10^9^	4.11 × 10^8^	1.71 × 10^9^	5.15 × 10^9^	4.52 × 10^9^	5.57 × 10^8^	2.54 × 10^9^
	avg	**2.20 × 10^7^**	4.77 × 10^8^	1.33 × 10^10^	1.93 × 10^9^	5.76 × 10^9^	1.20 × 10^10^	1.15 × 10^10^	5.38 × 10^8^	6.16 × 10^9^
F13	min	**1.42 × 10^4^**	2.64 × 10^4^	2.01 × 10^9^	3.87 × 10^4^	4.67 × 10^8^	2.95 × 10^8^	6.03 × 10^8^	1.45 × 10^5^	9.67 × 10^8^
	std	**5.51 × 10^4^**	1.02 × 10^8^	5.19 × 10^9^	1.70 × 10^8^	1.48 × 10^9^	8.59 × 10^9^	7.32 × 10^9^	1.88 × 10^7^	2.31 × 10^9^
	avg	**6.72 × 10^4^**	5.38 × 10^7^	1.04 × 10^10^	2.79 × 10^8^	3.08 × 10^9^	1.05 × 10^10^	9.35 × 10^9^	1.25 × 10^7^	3.60 × 10^9^
F14	min	**3.62 × 10^3^**	8.23 × 10^3^	3.47 × 10^6^	2.10 × 10^4^	1.20 × 10^6^	5.12 × 10^5^	3.19 × 10^5^	2.31 × 10^3^	5.73 × 10^5^
	std	**8.25 × 10^4^**	5.97 × 10^5^	8.10 × 10^6^	2.99 × 10^5^	3.61 × 10^6^	2.05 × 10^6^	7.98 × 10^6^	2.91 × 10^6^	1.38 × 10^6^
	avg	**8.29 × 10^4^**	4.40 × 10^5^	1.36 × 10^7^	4.92 × 10^5^	6.67 × 10^6^	2.43 × 10^6^	5.22 × 10^6^	5.99 × 10^5^	2.53 × 10^6^
F15	min	8.61 × 10^3^	1.19 × 10^4^	2.65 × 10^8^	**7.68 × 10^3^**	1.07 × 10^8^	1.32 × 10^7^	7.53 × 10^6^	9.74 × 10^3^	3.02 × 10^7^
	std	1.83 × 10^4^	2.28 × 10^7^	1.29 × 10^8^	**1.42 × 10^4^**	3.64 × 10^8^	1.51 × 10^9^	6.40 × 10^8^	1.53 × 10^5^	1.42 × 10^8^
	avg	3.12 × 10^4^	5.31 × 10^6^	5.01 × 10^8^	**2.67 × 10^4^**	6.07 × 10^8^	9.22 × 10^8^	5.82 × 10^8^	1.03 × 10^5^	2.15 × 10^8^
F16	min	2.61 × 10^3^	**2.50 × 10^3^**	5.80 × 10^3^	2.92 × 10^3^	4.73 × 10^3^	3.65 × 10^3^	3.81 × 10^3^	3.17 × 10^3^	4.17 × 10^3^
	std	**3.29 × 10^2^**	4.33 × 10^2^	1.69 × 10^3^	4.71 × 10^2^	4.35 × 10^2^	7.12 × 10^2^	1.09 × 10^3^	4.67 × 10^2^	4.71 × 10^2^
	avg	**3.15 × 10^3^**	3.29 × 10^3^	7.89 × 10^3^	3.80 × 10^3^	5.30 × 10^3^	4.71 × 10^3^	5.25 × 10^3^	3.82 × 10^3^	4.94 × 10^3^
F17	min	1.99 × 10^3^	**1.88 × 10^3^**	3.49 × 10^3^	2.12 × 10^3^	2.86 × 10^3^	2.46 × 10^3^	2.52 × 10^3^	2.04 × 10^3^	2.51 × 10^3^
	std	**2.52 × 10^2^**	2.68 × 10^2^	2.05 × 10^3^	2.70 × 10^2^	3.35 × 10^2^	3.28 × 10^3^	2.43 × 10^3^	3.03 × 10^2^	3.17 × 10^2^
	avg	**2.38 × 10^3^**	2.45 × 10^3^	5.29 × 10^3^	2.62 × 10^3^	3.53 × 10^3^	4.23 × 10^3^	4.42 × 10^3^	2.79 × 10^3^	3.22 × 10^3^
F18	min	1.41 × 10^5^	7.62 × 10^4^	7.49 × 10^6^	1.90 × 10^5^	6.38 × 10^6^	1.84 × 10^6^	1.73 × 10^6^	**5.46 × 10^4^**	6.92 × 10^6^
	std	3.16 × 10^6^	3.50 × 10^6^	1.00 × 10^8^	**7.89 × 10^5^**	3.79 × 10^7^	4.27 × 10^7^	9.38 × 10^7^	1.07 × 10^7^	3.35 × 10^7^
	avg	3.29 × 10^6^	3.23 × 10^6^	1.31 × 10^8^	**1.24 × 10^6^**	7.42 × 10^7^	5.36 × 10^7^	6.70 × 10^7^	3.25 × 10^6^	4.97 × 10^7^
F19	min	9.26 × 10^3^	**7.88 × 10^3^**	1.38 × 10^8^	1.73 × 10^5^	1.22 × 10^8^	5.81 × 10^6^	3.52 × 10^7^	4.18 × 10^4^	2.15 × 10^7^
	std	**5.45 × 10^5^**	6.20 × 10^6^	5.83 × 10^8^	1.17 × 10^6^	3.00 × 10^8^	1.63 × 10^9^	6.06 × 10^8^	2.68 × 10^7^	1.86 × 10^8^
	avg	**6.30 × 10^5^**	3.82 × 10^6^	8.77 × 10^8^	1.91 × 10^6^	5.98 × 10^8^	1.35 × 10^9^	7.66 × 10^8^	1.26 × 10^7^	2.43 × 10^8^
F20	min	2.31 × 10^3^	2.33 × 10^3^	2.87 × 10^3^	**2.24 × 10^3^**	3.00 × 10^3^	2.64 × 10^3^	2.61 × 10^3^	2.33 × 10^3^	2.69 × 10^3^
	std	1.81 × 10^2^	1.89 × 10^2^	1.72 × 10^2^	1.92 × 10^2^	**1.53 × 10^2^**	2.01 × 10^2^	2.66 × 10^2^	3.12 × 10^2^	2.46 × 10^2^
	avg	2.63 × 10^3^	2.71 × 10^3^	3.21 × 10^3^	**2.59 × 10^3^**	3.33 × 10^3^	3.01 × 10^3^	3.09 × 10^3^	2.90 × 10^3^	3.28 × 10^3^
F21	min	**2.22 × 10^3^**	2.36 × 10^3^	4.92 × 10^3^	3.05 × 10^3^	4.30 × 10^3^	3.40 × 10^3^	4.05 × 10^3^	2.40 × 10^3^	3.56 × 10^3^
	std	7.88 × 10^2^	9.12 × 10^2^	**2.89 × 10^2^**	4.69 × 10^2^	5.80 × 10^2^	6.93 × 10^2^	5.21 × 10^2^	9.04 × 10^2^	6.49 × 10^2^
	avg	**2.60 × 10^3^**	2.95 × 10^3^	5.74 × 10^3^	3.92 × 10^3^	5.76 × 10^3^	5.43 × 10^3^	5.86 × 10^3^	4.49 × 10^3^	5.49 × 10^3^
F22	min	**1.07 × 10^4^**	1.17 × 10^4^	2.32 × 10^4^	1.33 × 10^4^	2.35 × 10^4^	2.18 × 10^4^	1.96 × 10^4^	1.56 × 10^4^	1.98 × 10^4^
	std	1.66 × 10^3^	1.83 × 10^3^	**8.83 × 10^2^**	2.12 × 10^3^	1.10 × 10^3^	1.06 × 10^3^	1.87 × 10^3^	2.07 × 10^3^	1.45 × 10^3^
	avg	**1.45 × 10^4^**	1.59 × 10^4^	2.56 × 10^4^	1.72 × 10^4^	2.59 × 10^4^	2.34 × 10^4^	2.35 × 10^4^	2.02 × 10^4^	2.40 × 10^4^
F23	min	**4.02 × 10^3^**	4.35 × 10^3^	8.92 × 10^3^	5.06 × 10^3^	7.17 × 10^3^	6.54 × 10^3^	6.78 × 10^3^	5.14 × 10^3^	7.09 × 10^3^
	std	**4.13 × 10^2^**	7.07 × 10^2^	8.31 × 10^2^	8.49 × 10^2^	4.74 × 10^2^	6.83 × 10^2^	1.01 × 10^3^	8.08 × 10^2^	6.83 × 10^2^
	avg	**4.70 × 10^3^**	5.57 × 10^3^	1.05 × 10^4^	6.95 × 10^3^	8.10 × 10^3^	8.01 × 10^3^	9.02 × 10^3^	6.68 × 10^3^	8.06 × 10^3^
F24	min	**2.50 × 10^3^**	2.99 × 10^3^	6.24 × 10^3^	4.37 × 10^3^	6.36 × 10^3^	5.74 × 10^3^	6.41 × 10^3^	3.17 × 10^3^	5.51 × 10^3^
	std	1.59 × 10^3^	6.55 × 10^2^	**1.62 × 10^2^**	3.68 × 10^2^	2.96 × 10^2^	3.40 × 10^2^	1.81 × 10^2^	8.65 × 10^2^	4.61 × 10^2^
	avg	**4.37 × 10^3^**	5.63 × 10^3^	6.75 × 10^3^	5.43 × 10^3^	7.01 × 10^3^	6.69 × 10^3^	6.81 × 10^3^	5.64 × 10^3^	6.61 × 10^3^
F25	min	**2.90 × 10^3^**	2.97 × 10^3^	4.48 × 10^3^	3.10 × 10^3^	4.10 × 10^3^	4.08 × 10^3^	4.51 × 10^3^	3.05 × 10^3^	3.70 × 10^3^
	std	**2.18 × 10^1^**	1.30 × 10^2^	4.53 × 10^2^	3.42 × 10^1^	6.93 × 10^2^	1.10 × 10^3^	5.61 × 10^2^	2.19 × 10^2^	5.84 × 10^2^
	avg	**2.94 × 10^3^**	3.12 × 10^3^	5.14 × 10^3^	3.18 × 10^3^	5.10 × 10^3^	5.65 × 10^3^	5.32 × 10^3^	3.38 × 10^3^	4.68 × 10^3^
F26	min	**2.82 × 10^3^**	4.40 × 10^3^	1.07 × 10^4^	6.48 × 10^3^	9.12 × 10^3^	8.22 × 10^3^	9.30 × 10^3^	5.00 × 10^3^	7.69 × 10^3^
	std	1.36 × 10^3^	1.23 × 10^3^	**6.45 × 10^2^**	8.21 × 10^2^	7.67 × 10^2^	1.60 × 10^3^	1.24 × 10^3^	1.40 × 10^3^	8.62 × 10^2^
	avg	**5.50 × 10^3^**	6.85 × 10^3^	1.23 × 10^4^	8.17 × 10^3^	1.06 × 10^4^	1.09 × 10^4^	1.16 × 10^4^	8.65 × 10^3^	9.45 × 10^3^
F27	min	**3.24 × 10^3^**	3.27 × 10^3^	4.23 × 10^3^	3.68 × 10^3^	3.87 × 10^3^	3.41 × 10^3^	3.61 × 10^3^	3.29 × 10^3^	3.56 × 10^3^
	std	**4.33 × 10^1^**	8.44 × 10^1^	3.43 × 10^2^	1.52 × 10^2^	1.63 × 10^2^	2.50 × 10^2^	2.99 × 10^2^	1.23 × 10^2^	2.00 × 10^2^
	avg	**3.30 × 10^3^**	3.38 × 10^3^	4.79 × 10^3^	4.03 × 10^3^	4.13 × 10^3^	4.03 × 10^3^	4.22 × 10^3^	3.43 × 10^3^	4.03 × 10^3^
F28	min	**3.22 × 10^3^**	3.38 × 10^3^	5.61 × 10^3^	3.93 × 10^3^	5.29 × 10^3^	5.08 × 10^3^	5.39 × 10^3^	3.50 × 10^3^	4.87 × 10^3^
	std	**3.29 × 10^1^**	2.63 × 10^2^	6.73 × 10^2^	1.57 × 10^2^	5.70 × 10^2^	1.42 × 10^3^	8.00 × 10^2^	6.61 × 10^2^	7.75 × 10^2^
	avg	**3.29 × 10^3^**	3.74 × 10^3^	6.88 × 10^3^	4.22 × 10^3^	6.63 × 10^3^	7.29 × 10^3^	7.28 × 10^3^	4.07 × 10^3^	6.37 × 10^3^
F29	min	**3.99 × 10^3^**	4.06 × 10^3^	6.06 × 10^3^	4.20 × 10^3^	5.61 × 10^3^	4.98 × 10^3^	5.17 × 10^3^	4.35 × 10^3^	5.34 × 10^3^
	std	**2.01 × 10^2^**	3.81 × 10^2^	4.42 × 10^3^	4.49 × 10^2^	6.25 × 10^2^	2.96 × 10^3^	2.11 × 10^3^	5.76 × 10^2^	8.39 × 10^2^
	avg	**4.33 × 10^3^**	4.72 × 10^3^	1.15 × 10^4^	5.27 × 10^3^	6.65 × 10^3^	6.97 × 10^3^	7.68 × 10^3^	5.25 × 10^3^	6.41 × 10^3^
F30	min	2.14 × 10^6^	9.02 × 10^5^	5.32 × 10^8^	5.38 × 10^6^	1.13 × 10^8^	1.53 × 10^7^	1.17 × 10^8^	**8.55 × 10^5^**	9.81 × 10^7^
	std	**3.98 × 10^6^**	9.17 × 10^6^	9.56 × 10^8^	6.55 × 10^6^	2.05 × 10^8^	6.66 × 10^8^	4.73 × 10^8^	3.50 × 10^7^	2.92 × 10^8^
	avg	**6.00 × 10^6^**	1.30 × 10^7^	2.12 × 10^9^	1.45 × 10^7^	5.41 × 10^8^	3.99 × 10^8^	7.53 × 10^8^	3.05 × 10^7^	4.63 × 10^8^

**Table 4:** Test results for the dim = 10 CEC2022 test suite (the optimal results are shown in bold).

Func	Metrics	CMSCSO	SCSO	BBO	CAOA	FGO	MShOA	TGCOA	TOC	ZOA
F1	min	**300.00**	344.50	7600.20	438.03	8766.91	4271.50	10,254.10	445.10	3930.36
	std	**0.09**	2206.77	4585.50	416.40	5771.77	3917.54	5548.49	3131.85	6665.89
	avg	**300.04**	2484.09	15,524.17	1199.73	19,460.69	11,068.71	17,729.11	2252.33	17,172.87
F2	min	**400.00**	400.17	899.01	400.00	499.99	437.82	460.39	401.20	496.40
	std	**19.82**	32.16	433.59	41.68	91.81	573.43	498.47	33.31	200.90
	avg	**411.87**	447.30	1491.33	446.77	681.47	945.77	932.79	449.38	682.80
F3	min	**600.79**	604.85	629.64	610.84	623.51	622.70	626.05	605.10	622.86
	std	**5.69**	12.49	8.04	8.09	7.66	14.84	16.20	11.99	9.76
	avg	**610.33**	619.52	655.72	627.23	639.82	645.22	656.29	618.84	638.00
F4	min	812.93	806.87	846.56	**803.98**	849.23	833.19	827.98	813.51	835.96
	std	7.30	10.27	**6.06**	11.55	10.11	13.96	13.47	10.09	10.87
	avg	825.60	826.31	859.22	**820.93**	871.46	853.96	862.62	827.60	856.20
F5	min	**900.04**	902.45	1337.91	928.87	1124.50	978.86	1346.09	924.20	1060.19
	std	**78.83**	188.62	154.81	107.72	360.69	336.35	262.57	162.04	331.84
	avg	**950.22**	1104.66	1663.38	1040.91	1715.28	1523.68	1748.40	1036.31	1415.08
F6	min	1891.08	1975.19	1.42 × 10^8^	1919.21	1.27 × 10^7^	1.56 × 10^5^	2346.85	**1853.66**	1.50 × 10^5^
	std	1647.18	1878.99	3.15 × 10^8^	**637.10**	5.46 × 10^7^	1.12 × 10^8^	2.15 × 10^7^	2117.54	1.44 × 10^7^
	avg	4398.09	4953.74	5.30 × 10^8^	**2600.55**	5.86 × 10^7^	2.91 × 10^7^	8.10 × 10^6^	3371.53	1.26 × 10^7^
F7	min	**2021.06**	2026.70	2096.09	2031.40	2074.86	2049.51	2052.49	2027.00	2043.04
	std	11.73	20.05	15.73	**11.16**	29.16	28.92	53.90	36.95	22.74
	avg	**2037.24**	2051.08	2129.59	2056.35	2117.48	2098.09	2131.72	2072.06	2097.93
F8	min	**2206.71**	2221.74	2232.33	2222.72	2235.07	2228.26	2236.56	2211.17	2225.12
	std	**3.88**	6.23	80.32	4.95	20.63	39.17	88.57	22.87	11.13
	avg	**2223.68**	2229.17	2274.80	2230.19	2259.40	2244.52	2323.01	2234.70	2242.09
F9	min	2529.32	2531.15	2667.96	2613.64	2613.77	2587.36	2602.79	**2529.29**	2603.72
	std	37.17	40.01	56.16	**16.54**	25.19	66.20	78.17	47.47	41.65
	avg	**2540.54**	2590.11	2758.54	2649.56	2687.74	2721.82	2730.55	2557.77	2682.21
F10	min	**2500.24**	2500.33	2543.32	2501.40	2522.70	2502.43	2502.22	2500.45	2501.76
	std	**0.14**	133.26	175.30	38.32	78.35	116.06	453.78	288.63	92.53
	avg	**2500.54**	2599.00	2784.56	2514.33	2612.84	2617.03	2795.83	2624.59	2579.15
F11	min	**2600.00**	2606.28	2963.69	2600.99	7963.93	2907.20	2918.22	2751.75	4321.19
	std	164.37	223.78	357.87	**125.73**	4126.65	586.94	450.53	450.57	3363.17
	avg	**2729.53**	2873.07	3867.37	2892.66	14,756.63	4216.72	3799.37	3226.08	9119.91
F12	min	**2859.53**	2863.76	2876.42	2896.34	2892.52	2875.20	2886.05	2862.34	2873.67
	std	**1.47**	11.99	55.53	24.35	33.05	53.99	78.22	26.53	26.74
	avg	**2864.39**	2871.47	2948.82	2936.08	2969.31	2942.07	2979.35	2881.79	2928.94

**Table 5:** Average time comparison between CMSCSO and SCSO.

Benchmark Suite	Dim	CMSCSO/s	SCSO/s	Difference/s	Relative Overhead
CEC2017	30	0.2137	0.2027	0.011	5.43%
CEC2022	10	0.0618	0.0583	0.0035	6.00%

**Table 6:** Wilcoxon rank-sum test results for the dim = 30 CEC2017 test suite (*p* < 0.05: significant difference between the two algorithms; *p* ≥ 0.05: no significant difference between the two algorithms, with corresponding entries bold).

Func	SCSO	BBO	CAOA	FGO	MShOA	TGCOA	TOC	ZOA
F1	3.0199 × 10^−11^	3.0199 × 10^−11^	3.0199 × 10^−11^	3.0199 × 10^−11^	3.0199 × 10^−11^	3.0199 × 10^−11^	3.0199 × 10^−11^	3.0199 × 10^−11^
F3	1.4643 × 10^−10^	3.0199 × 10^−11^	2.6099 × 10^−10^	3.0199 × 10^−11^	3.0199 × 10^−11^	3.0199 × 10^−11^	3.1589 × 10^−10^	3.0199 × 10^−11^
F4	5.4941 × 10^−11^	3.0199 × 10^−11^	3.0199 × 10^−11^	3.0199 × 10^−11^	3.0199 × 10^−11^	3.0199 × 10^−11^	3.0199 × 10^−11^	3.0199 × 10^−11^
F5	1.2860 × 10^−6^	3.0199 × 10^−11^	1.1711 × 10^−2^	3.0199 × 10^−11^	3.0199 × 10^−11^	3.0199 × 10^−11^	3.4971 × 10^−9^	3.0199 × 10^−11^
F6	6.0104 × 10^−8^	3.0199 × 10^−11^	5.8587 × 10^−6^	4.5043 × 10^−11^	3.0199 × 10^−11^	3.0199 × 10^−11^	9.7917 × 10^−5^	3.6897 × 10^−11^
F7	1.8608 × 10^−6^	3.0199 × 10^−11^	**1.4945 × 10^−1^**	3.0199 × 10^−11^	3.0199 × 10^−11^	3.0199 × 10^−11^	1.4110 × 10^−9^	3.0199 × 10^−11^
F8	2.1540 × 10^−6^	3.0199 × 10^−11^	**8.1875 × 10^−1^**	3.0199 × 10^−11^	3.0199 × 10^−11^	3.0199 × 10^−11^	9.7555 × 10^−10^	3.0199 × 10^−11^
F9	3.0811 × 10^−8^	3.0199 × 10^−11^	3.5012 × 10^−3^	3.0199 × 10^−11^	3.3384 × 10^−11^	3.0199 × 10^−11^	2.7086 × 10^−2^	3.0199 × 10^−11^
F10	3.2555 × 10^−7^	3.0199 × 10^−11^	2.4913 × 10^−6^	3.0199 × 10^−11^	3.0199 × 10^−11^	3.0199 × 10^−11^	6.0658 × 10^−11^	3.0199 × 10^−11^
F11	3.0199 × 10^−11^	3.0199 × 10^−11^	3.0199 × 10^−11^	3.0199 × 10^−11^	3.0199 × 10^−11^	3.0199 × 10^−11^	3.0199 × 10^−11^	3.0199 × 10^−11^
F12	4.5043 × 10^−11^	3.0199 × 10^−11^	3.0199 × 10^−11^	3.0199 × 10^−11^	3.0199 × 10^−11^	3.0199 × 10^−11^	3.0199 × 10^−11^	3.0199 × 10^−11^
F13	2.3768 × 10^−7^	3.0199 × 10^−11^	1.0702 × 10^−9^	3.0199 × 10^−11^	3.0199 × 10^−11^	3.0199 × 10^−11^	6.0658 × 10^−11^	3.0199 × 10^−11^
F14	2.1566 × 10^−3^	3.0199 × 10^−11^	1.1023 × 10^−8^	3.0199 × 10^−11^	3.0199 × 10^−11^	3.0199 × 10^−11^	**1.1199 × 10^−1^**	3.0199 × 10^−11^
F15	3.0811 × 10^−8^	3.0199 × 10^−11^	**5.0114 × 10^−1^**	3.0199 × 10^−11^	3.0199 × 10^−11^	3.0199 × 10^−11^	4.2175 × 10^−4^	3.0199 × 10^−11^
F16	**2.0095 × 10^−1^**	3.0199 × 10^−11^	1.1077 × 10^−6^	3.0199 × 10^−11^	4.9752 × 10^−11^	3.6897 × 10^−11^	1.7294 × 10^−7^	3.0199 × 10^−11^
F17	**1.5798 × 10^−1^**	3.0199 × 10^−11^	7.6973 × 10^−4^	4.0772 × 10^−11^	1.6947 × 10^−9^	8.9934 × 10^−11^	2.6784 × 10^−6^	2.8716 × 10^−10^
F18	**5.2014 × 10^−1^**	4.5043 × 10^−11^	1.9527 × 10^−3^	4.5043 × 10^−11^	1.4294 × 10^−8^	8.4848 × 10^−9^	**4.9426 × 10^−5^**	5.4941 × 10^−11^
F19	9.7917 × 10^−5^	3.0199 × 10^−11^	2.2780 × 10^−5^	3.0199 × 10^−11^	3.0199 × 10^−11^	3.0199 × 10^−11^	1.2477 × 10^−4^	3.0199 × 10^−11^
F20	**8.5000 × 10^−2^**	8.1527 × 10^−11^	**4.6427 × 10^−1^**	4.5043 × 10^−11^	3.3520 × 10^−8^	1.1023 × 10^−8^	2.6806 × 10^−4^	2.1544 × 10^−10^
F21	1.1937 × 10^−6^	3.0199 × 10^−11^	9.5332 × 10^−7^	5.4941 × 10^−11^	1.2057 × 10^−10^	4.9752 × 10^−11^	1.8567 × 10^−9^	7.3891 × 10^−11^
F22	1.0763 × 10^−2^	3.0199 × 10^−11^	4.4205 × 10^−6^	3.0199 × 10^−11^	3.0199 × 10^−11^	3.0199 × 10^−11^	8.9934 × 10^−11^	3.0199 × 10^−11^
F23	2.4913 × 10^−6^	3.0199 × 10^−11^	8.1527 × 10^−11^	3.0199 × 10^−11^	3.0199 × 10^−11^	3.0199 × 10^−11^	7.3891 × 10^−11^	3.0199 × 10^−11^
F24	1.5846 × 10^−4^	3.0199 × 10^−11^	**3.7108 × 10^−1^**	3.0199 × 10^−11^	8.1527 × 10^−11^	3.0199 × 10^−11^	3.6709 × 10^−3^	8.1014 × 10^−10^
F25	4.9752 × 10^−11^	3.0199 × 10^−11^	3.0199 × 10^−11^	3.0199 × 10^−11^	3.0199 × 10^−11^	3.0199 × 10^−11^	3.0199 × 10^−11^	3.0199 × 10^−11^
F26	4.4592 × 10^−4^	3.0199 × 10^−11^	8.1014 × 10^−10^	3.0199 × 10^−11^	3.0199 × 10^−11^	3.0199 × 10^−11^	2.9215 × 10^−9^	3.6897 × 10^−11^
F27	4.0840 × 10^−5^	3.0199 × 10^−11^	3.0199 × 10^−11^	3.0199 × 10^−11^	3.0199 × 10^−11^	3.0199 × 10^−11^	5.1857 × 10^−7^	3.0199 × 10^−11^
F28	3.0199 × 10^−11^	3.0199 × 10^−11^	3.0199 × 10^−11^	3.0199 × 10^−11^	3.0199 × 10^−11^	3.0199 × 10^−11^	3.0199 × 10^−11^	3.0199 × 10^−11^
F29	4.9426 × 10^−5^	3.0199 × 10^−11^	1.4110 × 10^−9^	3.0199 × 10^−11^	3.0199 × 10^−11^	3.0199 × 10^−11^	9.7555 × 10^−10^	3.0199 × 10^−11^
F30	3.1830 × 10^−3^	3.0199 × 10^−11^	6.0104 × 10^−8^	3.0199 × 10^−11^	3.3384 × 10^−11^	3.0199 × 10^−11^	1.3367 × 10^−5^	3.0199 × 10^−11^
Win/Tie/Loss	25/4/0	29/0/0	23/5/1	29/0/0	29/0/0	29/0/0	27/1/1	29/0/0

**Table 7:** Wilcoxon rank-sum test results for the dim = 10 CEC2022 test suite (*p* < 0.05: significant difference between the two algorithms; *p* ≥ 0.05: no significant difference between the two algorithms, with corresponding entries bold).

Func	SCSO	BBO	CAOA	FGO	MShOA	TGCOA	TOC	ZOA
F1	3.0199 × 10^−11^	3.0199 × 10^−11^	3.0199 × 10^−11^	3.0199 × 10^−11^	3.0199 × 10^−11^	3.0199 × 10^−11^	3.0199 × 10^−11^	3.0199 × 10^−11^
F2	1.4067 × 10^−4^	3.0199 × 10^−11^	3.3679 × 10^−4^	3.0199 × 10^−11^	5.4941 × 10^−11^	4.0772 × 10^−11^	2.4913 × 10^−6^	3.0199 × 10^−11^
F3	1.6798 × 10^−3^	3.0199 × 10^−11^	1.8567 × 10^−9^	3.6897 × 10^−11^	3.6897 × 10^−11^	3.0199 × 10^−11^	3.5638 × 10^−4^	4.5043 × 10^−11^
F4	6.1001 × 10^−1^	3.0199 × 10^−11^	2.7086 × 10^−2^	3.0199 × 10^−11^	8.1527 × 10^−11^	1.2057 × 10^−10^	**6.7350 × 10^−1^**	3.6897 × 10^−11^
F5	6.7362 × 10^−6^	3.0199 × 10^−11^	2.8790 × 10^−6^	3.6897 × 10^−11^	1.4643 × 10^−10^	3.0199 × 10^−11^	8.8829 × 10^−6^	9.9186 × 10^−11^
F6	2.3985 × 10^−1^	3.0180 × 10^−11^	5.4620 × 10^−6^	3.0199 × 10^−11^	3.0199 × 10^−11^	1.3853 × 10^−6^	**2.7548 × 10^−3^**	3.0199 × 10^−11^
F7	1.9527 × 10^−3^	3.0199 × 10^−11^	4.8011 × 10^−7^	3.0199 × 10^−11^	9.9186 × 10^−11^	5.4941 × 10^−11^	1.2860 × 10^−6^	6.0658 × 10^−11^
F8	2.9590 × 10^−5^	3.0199 × 10^−11^	2.7829 × 10^−7^	3.0199 × 10^−11^	3.0199 × 10^−11^	3.0199 × 10^−11^	1.0907 × 10^−5^	8.1527 × 10^−11^
F9	1.6980 × 10^−8^	3.6897 × 10^−11^	7.1186 × 10^−9^	1.2057 × 10^−10^	1.2057 × 10^−10^	9.9186 × 10^−11^	1.1738 × 10^−3^	3.1589 × 10^−10^
F10	4.4440 × 10^−7^	3.0199 × 10^−11^	3.0199 × 10^−11^	3.0199 × 10^−11^	3.0199 × 10^−11^	3.0199 × 10^−11^	7.0881 × 10^−8^	3.0199 × 10^−11^
F11	1.9527 × 10^−3^	3.3384 × 10^−11^	3.0811 × 10^−8^	3.0199 × 10^−11^	3.6897 × 10^−11^	4.9752 × 10^−11^	1.4733 × 10^−7^	3.0199 × 10^−11^
F12	1.8608 × 10^−6^	2.9991 × 10^−11^	3.0199 × 10^−11^	3.0199 × 10^−11^	3.0199 × 10^−11^	3.0199 × 10^−11^	9.5332 × 10^−7^	3.0199 × 10^−11^
Win/Tie/Loss	10/2/0	12/0/0	10/0/2	12/0/0	12/0/0	12/0/0	10/1/1	12/0/0

**Table 8:** Strategy configurations of SCSO, CMSCSO, and the ablation variants (×: this improvement strategy is not included; √: this improvement strategy is not included).

Algorithm	GPS	OBL	PIC	RRS
SCSO	×	×	×	×
CMSCSO	√	√	√	√
SCSOGPS	√	×	×	×
SCSOOBL	×	√	×	×
SCSOPIC	×	×	√	×
SCSORRS	×	×	×	√

**Table 9:** Ablation experiment test results for the dim = 10 CEC2022 test suite (the optimal results are shown in bold).

Func	Metrics	SCSOGPS	SCSOOBL	SCSOPIC	SCSORRS	CMSCSO	SCSO
F1	std	2312.96	2013.65	1149.68	41.38	**0.09**	2206.77
	avg	2701.29	3213.13	1297.27	343.14	**300.04**	2484.09
F2	std	33.42	29.55	26.57	25.35	**19.82**	32.16
	avg	446.80	438.15	426.39	418.89	**411.87**	447.30
F3	std	9.86	11.63	9.01	10.07	**5.69**	12.49
	avg	613.35	618.63	614.11	615.94	**610.33**	619.52
F4	std	9.53	8.14	7.62	7.42	**7.30**	10.27
	avg	826.78	828.44	828.61	826.39	**825.60**	826.31
F5	std	195.54	195.60	142.05	106.60	**78.83**	188.62
	avg	1161.10	1169.62	1071.85	979.92	**950.22**	1104.66
F6	std	2094.54	1919.28	2054.91	2057.25	**1647.18**	1878.99
	avg	4583.81	4641.85	4482.02	4780.07	**4398.09**	4953.74
F7	std	27.09	23.07	14.55	13.58	**11.73**	20.05
	avg	2062.64	2055.23	2042.68	2045.33	**2037.24**	2051.08
F8	std	6.64	4.84	4.81	**3.36**	3.88	6.23
	avg	2228.76	2229.84	2227.85	2224.31	**2223.68**	2229.17
F9	std	52.88	46.35	38.16	**31.43**	37.17	40.01
	avg	2619.68	2601.97	2587.87	2563.45	**2540.54**	2590.11
F10	std	22.51	63.95	61.25	62.95	**0.14**	133.26
	avg	2504.81	2572.31	2549.51	2562.35	**2500.54**	2599.00
F11	std	207.13	236.42	186.17	172.13	**164.37**	223.78
	avg	2913.90	2885.65	2855.73	2796.25	**2729.53**	2873.07
F12	std	3.04	17.51	6.94	9.84	**1.47**	11.99
	avg	2865.24	2875.95	2867.66	2868.27	**2864.39**	2871.47

**Table 10:** Friedman average rank results for the dim = 10 CEC2022 test suite.

Algorithm	SCSOGPS	SCSOOBL	SCSOPIC	SCSORRS	CMSCSO	SCSO
Average rank	4.46	4.95	3.04	2.75	1.08	4.71
Final rank	4	6	3	2	1	5

**Table 11:** Sensitivity analysis results of CMSCSO on the CEC2022-F2 (the optimal results are shown in bold).

*N*	Dim	*p*_min_	*p*_max_	Ave ± Std
10	10	0.1	0.9	437.19 ± 28.84
10	10	0.2	0.8	**424.97 ± 25.79**
10	10	0.3	0.7	428.98 ± 26.48
10	10	0.4	0.6	429.96 ± 29.59
10	20	0.1	0.9	517.44 ± 41.92
10	20	0.2	0.8	**499.82 ± 36.04**
10	20	0.3	0.7	510.51 ± 44.63
10	20	0.4	0.6	516.37 ± 47.36
20	10	0.1	0.9	417.44 ± 21.97
20	10	0.2	0.8	**416.10 ± 23.55**
20	10	0.3	0.7	425.41 ± 27.97
20	10	0.4	0.6	420.15 ± 23.93
20	20	0.1	0.9	**474.46 ± 22.22**
20	20	0.2	0.8	474.57 ± 21.14
20	20	0.3	0.7	485.42 ± 38.82
20	20	0.4	0.6	481.56 ± 26.23
30	10	0.1	0.9	417.45 ± 23.48
30	10	0.2	0.8	**413.81 ± 21.29**
30	10	0.3	0.7	420.91 ± 26.64
30	10	0.4	0.6	416.07 ± 23.28
30	20	0.1	0.9	473.02 ± 26.84
30	20	0.2	0.8	470.91 ± 22.11
30	20	0.3	0.7	477.01 ± 32.45
30	20	0.4	0.6	**470.35 ± 19.53**

**Table 12:** Sensitivity analysis results of CMSCSO on the CEC2022-F12 (the optimal results are shown in bold).

*N*	Dim	*p*_min_	*p*_max_	Ave ± Std
10	10	0.1	0.9	2868.80 ± 6.25
10	10	0.2	0.8	**2866.06 ± 1.93**
10	10	0.3	0.7	2871.94 ± 8.66
10	10	0.4	0.6	2869.92 ± 7.96
10	20	0.1	0.9	3028.39 ± 49.22
10	20	0.2	0.8	**3001.46 ± 38.60**
10	20	0.3	0.7	3031.91 ± 63.92
10	20	0.4	0.6	3033.54 ± 56.43
20	10	0.1	0.9	2865.93 ± 3.01
20	10	0.2	0.8	**2864.87 ± 2.19**
20	10	0.3	0.7	2866.22 ± 2.12
20	10	0.4	0.6	2865.15 ± 1.76
20	20	0.1	0.9	3018.19 ± 51.59
20	20	0.2	0.8	**2994.54 ± 43.62**
20	20	0.3	0.7	3020.75 ± 41.92
20	20	0.4	0.6	3026.33 ± 54.36
30	10	0.1	0.9	**2864.57 ± 1.97**
30	10	0.2	0.8	2864.95 ± 1.50
30	10	0.3	0.7	2864.94 ± 1.35
30	10	0.4	0.6	2865.46 ± 2.00
30	20	0.1	0.9	3004.27 ± 32.21
30	20	0.2	0.8	**3003.27 ± 44.67**
30	20	0.3	0.7	3013.71 ± 51.17
30	20	0.4	0.6	3016.58 ± 55.54

**Table 13:** Test results for the dim = 50 CEC2017 test suite.

Func	Metrics	CMSCSO	SCSO	BBO	CAOA	FGO	MShOA	TGCOA	TOC	ZOA
F1	min	3.30 × 10^7^	1.12 × 10^10^	9.24 × 10^10^	4.00 × 10^10^	8.57 × 10^10^	9.29 × 10^10^	7.40 × 10^10^	1.70 × 10^10^	6.06 × 10^10^
	std	7.52 × 10^7^	8.04 × 10^9^	6.99 × 10^9^	3.70 × 10^9^	1.52 × 10^10^	1.02 × 10^10^	1.32 × 10^10^	1.38 × 10^10^	1.65 × 10^10^
	avg	1.35 × 10^8^	2.88 × 10^10^	1.16 × 10^11^	4.48 × 10^10^	1.10 × 10^11^	1.11 × 10^11^	1.07 × 10^11^	3.81 × 10^10^	9.26 × 10^10^
F3	min	9.37 × 10^4^	8.70 × 10^4^	1.47 × 10^5^	1.18 × 10^5^	2.57 × 10^5^	1.57 × 10^5^	1.41 × 10^5^	1.30 × 10^5^	2.47 × 10^5^
	std	2.84 × 10^4^	1.73 × 10^4^	1.66 × 10^5^	1.57 × 10^4^	6.52 × 10^5^	4.92 × 10^4^	7.76 × 10^4^	9.84 × 10^4^	1.68 × 10^5^
	avg	1.35 × 10^5^	1.30 × 10^5^	2.98 × 10^5^	1.48 × 10^5^	5.96 × 10^5^	2.14 × 10^5^	3.18 × 10^5^	2.10 × 10^5^	4.30 × 10^5^
F4	min	5.04 × 10^2^	1.34 × 10^3^	2.38 × 10^4^	8.50 × 10^3^	1.79 × 10^4^	2.04 × 10^4^	2.03 × 10^4^	1.91 × 10^3^	1.42 × 10^4^
	std	7.04 × 10^1^	2.52 × 10^3^	4.06 × 10^3^	6.90 × 10^2^	7.19 × 10^3^	7.64 × 10^3^	8.07 × 10^3^	2.70 × 10^3^	6.49 × 10^3^
	avg	7.09 × 10^2^	4.75 × 10^3^	3.72 × 10^4^	9.94 × 10^3^	2.97 × 10^4^	3.47 × 10^4^	3.45 × 10^4^	6.60 × 10^3^	2.51 × 10^4^
F5	min	7.99 × 10^2^	8.80 × 10^2^	1.18 × 10^3^	7.97 × 10^2^	1.20 × 10^3^	1.07 × 10^3^	1.11 × 10^3^	1.01 × 10^3^	1.13 × 10^3^
	std	3.34 × 10^1^	4.72 × 10^1^	1.73 × 10^1^	6.23 × 10^1^	4.13 × 10^1^	4.74 × 10^1^	4.04 × 10^1^	4.80 × 10^1^	5.39 × 10^1^
	avg	8.65 × 10^2^	9.63 × 10^2^	1.22 × 10^3^	9.00 × 10^2^	1.28 × 10^3^	1.19 × 10^3^	1.22 × 10^3^	1.10 × 10^3^	1.24 × 10^3^
F6	min	6.48 × 10^2^	6.58 × 10^2^	7.04 × 10^2^	6.56 × 10^2^	6.80 × 10^2^	6.92 × 10^2^	6.94 × 10^2^	6.59 × 10^2^	6.86 × 10^2^
	std	5.86 × 10^0^	7.49 × 10^0^	3.35 × 10^0^	5.35 × 10^0^	8.95 × 10^0^	7.16 × 10^0^	8.71 × 10^0^	9.27 × 10^0^	7.26 × 10^0^
	avg	6.63 × 10^2^	6.76 × 10^2^	7.11 × 10^2^	6.67 × 10^2^	7.02 × 10^2^	7.02 × 10^2^	7.12 × 10^2^	6.84 × 10^2^	7.03 × 10^2^
F7	min	1.17 × 10^3^	1.45 × 10^3^	1.97 × 10^3^	1.26 × 10^3^	2.25 × 10^3^	1.94 × 10^3^	1.98 × 10^3^	1.54 × 10^3^	2.12 × 10^3^
	std	1.64 × 10^2^	1.25 × 10^2^	4.53 × 10^1^	1.38 × 10^2^	2.48 × 10^2^	8.06 × 10^1^	5.24 × 10^1^	1.96 × 10^2^	2.14 × 10^2^
	avg	1.47 × 10^3^	1.71 × 10^3^	2.07 × 10^3^	1.54 × 10^3^	2.73 × 10^3^	2.10 × 10^3^	2.09 × 10^3^	1.94 × 10^3^	2.45 × 10^3^
F8	min	1.08 × 10^3^	1.22 × 10^3^	1.51 × 10^3^	1.07 × 10^3^	1.50 × 10^3^	1.45 × 10^3^	1.44 × 10^3^	1.29 × 10^3^	1.39 × 10^3^
	std	4.95 × 10^1^	3.72 × 10^1^	1.87 × 10^1^	6.01 × 10^1^	5.23 × 10^1^	4.40 × 10^1^	4.50 × 10^1^	5.30 × 10^1^	5.52 × 10^1^
	avg	1.19 × 10^3^	1.28 × 10^3^	1.56 × 10^3^	1.22 × 10^3^	1.59 × 10^3^	1.53 × 10^3^	1.56 × 10^3^	1.40 × 10^3^	1.53 × 10^3^
F9	min	9.06 × 10^3^	1.52 × 10^4^	3.30 × 10^4^	1.17 × 10^4^	3.32 × 10^4^	3.06 × 10^4^	3.41 × 10^4^	1.41 × 10^4^	2.96 × 10^4^
	std	2.59 × 10^3^	3.16 × 10^3^	2.77 × 10^3^	4.97 × 10^3^	6.21 × 10^3^	5.18 × 10^3^	6.48 × 10^3^	5.58 × 10^3^	7.98 × 10^3^
	avg	1.52 × 10^4^	2.17 × 10^4^	4.19 × 10^4^	1.96 × 10^4^	4.62 × 10^4^	4.00 × 10^4^	4.47 × 10^4^	2.23 × 10^4^	4.38 × 10^4^
F10	min	6.71 × 10^3^	8.85 × 10^3^	1.50 × 10^4^	8.16 × 10^3^	1.48 × 10^4^	1.21 × 10^4^	1.38 × 10^4^	1.11 × 10^4^	1.40 × 10^4^
	std	1.00 × 10^3^	7.66 × 10^2^	4.53 × 10^2^	1.78 × 10^3^	5.66 × 10^2^	1.05 × 10^3^	7.48 × 10^2^	1.05 × 10^3^	6.75 × 10^2^
	avg	8.82 × 10^3^	1.03 × 10^4^	1.60 × 10^4^	1.16 × 10^4^	1.64 × 10^4^	1.46 × 10^4^	1.54 × 10^4^	1.39 × 10^4^	1.56 × 10^4^
F11	min	2.25 × 10^3^	5.82 × 10^3^	2.98 × 10^4^	1.04 × 10^4^	2.97 × 10^4^	2.51 × 10^4^	2.42 × 10^4^	8.39 × 10^3^	2.60 × 10^4^
	std	1.28 × 10^3^	4.96 × 10^3^	1.00 × 10^4^	2.64 × 10^3^	1.60 × 10^4^	5.57 × 10^3^	1.19 × 10^4^	1.23 × 10^4^	1.57 × 10^4^
	avg	3.98 × 10^3^	1.43 × 10^4^	4.17 × 10^4^	1.49 × 10^4^	6.27 × 10^4^	3.46 × 10^4^	5.28 × 10^4^	2.20 × 10^4^	5.80 × 10^4^
F12	min	2.72 × 10^7^	2.06 × 10^8^	5.63 × 10^10^	1.83 × 10^10^	3.49 × 10^10^	2.74 × 10^10^	2.82 × 10^10^	1.40 × 10^9^	2.54 × 10^10^
	std	8.60 × 10^7^	4.60 × 10^9^	1.42 × 10^10^	3.33 × 10^9^	8.85 × 10^9^	2.04 × 10^10^	2.40 × 10^10^	3.04 × 10^9^	9.89 × 10^9^
	avg	1.45 × 10^8^	5.61 × 10^9^	8.43 × 10^10^	2.41 × 10^10^	4.79 × 10^10^	6.68 × 10^10^	8.22 × 10^10^	4.96 × 10^9^	4.65 × 10^10^
F13	min	2.90 × 10^4^	2.60 × 10^7^	3.48 × 10^10^	5.42 × 10^9^	1.17 × 10^10^	2.52 × 10^9^	8.62 × 10^9^	8.13 × 10^7^	8.98 × 10^9^
	std	6.20 × 10^4^	3.68 × 10^9^	1.14 × 10^10^	2.12 × 10^9^	6.41 × 10^9^	2.39 × 10^10^	1.33 × 10^10^	1.30 × 10^9^	8.18 × 10^9^
	avg	1.25 × 10^5^	1.73 × 10^9^	5.78 × 10^10^	8.90 × 10^9^	2.06 × 10^10^	3.54 × 10^10^	3.31 × 10^10^	9.43 × 10^8^	1.98 × 10^10^
F14	min	9.79 × 10^4^	9.87 × 10^4^	6.23 × 10^7^	1.65 × 10^6^	1.52 × 10^7^	2.11 × 10^6^	2.26 × 10^6^	5.02 × 10^4^	1.21 × 10^7^
	std	4.86 × 10^5^	5.02 × 10^6^	7.46 × 10^7^	5.25 × 10^6^	3.64 × 10^7^	3.06 × 10^7^	7.10 × 10^7^	7.71 × 10^6^	2.45 × 10^7^
	avg	7.90 × 10^5^	3.07 × 10^6^	1.73 × 10^8^	9.85 × 10^6^	5.71 × 10^7^	3.57 × 10^7^	7.21 × 10^7^	5.09 × 10^6^	4.64 × 10^7^
F15	min	1.53 × 10^4^	4.79 × 10^4^	5.15 × 10^9^	4.13 × 10^4^	2.06 × 10^9^	1.54 × 10^9^	6.84 × 10^8^	3.09 × 10^6^	8.94 × 10^8^
	std	1.61 × 10^4^	4.26 × 10^8^	2.96 × 10^9^	6.60 × 10^7^	2.12 × 10^9^	5.97 × 10^9^	3.82 × 10^9^	1.82 × 10^8^	1.93 × 10^9^
	avg	4.06 × 10^4^	2.72 × 10^8^	1.19 × 10^10^	6.58 × 10^7^	5.89 × 10^9^	8.61 × 10^9^	7.05 × 10^9^	9.11 × 10^7^	3.58 × 10^9^
F16	min	3.01 × 10^3^	3.78 × 10^3^	8.31 × 10^3^	4.13 × 10^3^	6.50 × 10^3^	6.15 × 10^3^	6.66 × 10^3^	4.98 × 10^3^	5.90 × 10^3^
	std	5.80 × 10^2^	6.16 × 10^2^	1.46 × 10^3^	8.51 × 10^2^	8.29 × 10^2^	1.26 × 10^3^	2.39 × 10^3^	4.81 × 10^2^	8.46 × 10^2^
	avg	4.18 × 10^3^	4.67 × 10^3^	1.20 × 10^4^	6.04 × 10^3^	8.18 × 10^3^	8.04 × 10^3^	9.60 × 10^3^	5.81 × 10^3^	7.71 × 10^3^
F17	min	2.83 × 10^3^	3.14 × 10^3^	7.42 × 10^3^	2.93 × 10^3^	5.38 × 10^3^	4.37 × 10^3^	5.00 × 10^3^	3.82 × 10^3^	4.59 × 10^3^
	std	3.42 × 10^2^	4.63 × 10^2^	6.28 × 10^3^	4.82 × 10^2^	3.74 × 10^3^	2.23 × 10^4^	1.00 × 10^4^	3.72 × 10^2^	6.32 × 10^3^
	avg	3.51 × 10^3^	3.86 × 10^3^	1.62 × 10^4^	3.66 × 10^3^	9.19 × 10^3^	2.03 × 10^4^	1.40 × 10^4^	4.39 × 10^3^	7.64 × 10^3^
F18	min	3.73 × 10^5^	1.44 × 10^6^	1.05 × 10^8^	4.98 × 10^6^	3.23 × 10^7^	3.24 × 10^7^	1.53 × 10^7^	5.29 × 10^5^	4.49 × 10^7^
	std	4.40 × 10^6^	1.27 × 10^7^	5.74 × 10^7^	7.51 × 10^6^	9.56 × 10^7^	1.34 × 10^8^	9.19 × 10^7^	2.04 × 10^7^	7.29 × 10^7^
	avg	5.07 × 10^6^	1.47 × 10^7^	2.18 × 10^8^	2.20 × 10^7^	2.37 × 10^8^	1.59 × 10^8^	1.54 × 10^8^	1.55 × 10^7^	1.63 × 10^8^
F19	min	6.83 × 10^4^	2.01 × 10^5^	9.03 × 10^8^	1.30 × 10^5^	6.21 × 10^8^	2.89 × 10^8^	4.32 × 10^8^	2.55 × 10^6^	3.87 × 10^8^
	std	4.30 × 10^5^	1.43 × 10^8^	1.58 × 10^9^	6.38 × 10^6^	5.87 × 10^8^	3.13 × 10^9^	2.11 × 10^9^	5.02 × 10^7^	7.97 × 10^8^
	avg	6.19 × 10^5^	3.71 × 10^7^	3.90 × 10^9^	3.83 × 10^6^	1.64 × 10^9^	5.26 × 10^9^	3.40 × 10^9^	4.39 × 10^7^	1.60 × 10^9^
F20	min	2.55 × 10^3^	3.12 × 10^3^	4.01 × 10^3^	2.82 × 10^3^	4.56 × 10^3^	3.77 × 10^3^	3.74 × 10^3^	3.10 × 10^3^	4.30 × 10^3^
	std	3.79 × 10^2^	2.54 × 10^2^	1.72 × 10^2^	3.01 × 10^2^	1.93 × 10^2^	2.95 × 10^2^	2.85 × 10^2^	4.35 × 10^2^	2.19 × 10^2^
	avg	3.48 × 10^3^	3.54 × 10^3^	4.46 × 10^3^	3.47 × 10^3^	4.90 × 10^3^	4.32 × 10^3^	4.56 × 10^3^	3.77 × 10^3^	4.71 × 10^3^
F21	min	2.29 × 10^3^	3.06 × 10^3^	8.54 × 10^3^	5.25 × 10^3^	8.69 × 10^3^	7.70 × 10^3^	8.18 × 10^3^	7.10 × 10^3^	8.41 × 10^3^
	std	1.02 × 10^3^	1.11 × 10^3^	2.03 × 10^2^	4.89 × 10^2^	4.53 × 10^2^	4.50 × 10^2^	3.20 × 10^2^	4.67 × 10^2^	5.31 × 10^2^
	avg	5.87 × 10^3^	6.39 × 10^3^	8.99 × 10^3^	6.26 × 10^3^	9.62 × 10^3^	8.86 × 10^3^	8.99 × 10^3^	8.04 × 10^3^	9.42 × 10^3^
F22	min	2.04 × 10^4^	1.99 × 10^4^	3.84 × 10^4^	2.36 × 10^4^	4.18 × 10^4^	3.73 × 10^4^	3.40 × 10^4^	3.02 × 10^4^	3.82 × 10^4^
	std	2.69 × 10^3^	3.60 × 10^3^	1.57 × 10^3^	3.26 × 10^3^	1.10 × 10^3^	2.16 × 10^3^	2.68 × 10^3^	2.50 × 10^3^	1.69 × 10^3^
	avg	2.48 × 10^4^	2.84 × 10^4^	4.34 × 10^4^	3.05 × 10^4^	4.37 × 10^4^	4.12 × 10^4^	4.06 × 10^4^	3.46 × 10^4^	4.18 × 10^4^
F23	min	6.43 × 10^3^	7.03 × 10^3^	1.38 × 10^4^	9.18 × 10^3^	1.22 × 10^4^	1.09 × 10^4^	1.27 × 10^4^	8.78 × 10^3^	1.13 × 10^4^
	std	9.05 × 10^2^	1.08 × 10^3^	1.07 × 10^3^	1.16 × 10^3^	7.33 × 10^2^	1.48 × 10^3^	1.32 × 10^3^	1.55 × 10^3^	7.99 × 10^2^
	avg	7.85 × 10^3^	9.15 × 10^3^	1.70 × 10^4^	1.14 × 10^4^	1.39 × 10^4^	1.37 × 10^4^	1.46 × 10^4^	1.15 × 10^4^	1.31 × 10^4^
F24	min	2.65 × 10^3^	8.02 × 10^3^	9.72 × 10^3^	6.82 × 10^3^	1.03 × 10^4^	9.70 × 10^3^	9.62 × 10^3^	8.55 × 10^3^	9.56 × 10^3^
	std	1.03 × 10^3^	2.79 × 10^2^	1.76 × 10^2^	5.69 × 10^2^	3.46 × 10^2^	2.49 × 10^2^	2.59 × 10^2^	5.29 × 10^2^	4.24 × 10^2^
	avg	7.98 × 10^3^	8.62 × 10^3^	1.02 × 10^4^	8.11 × 10^3^	1.10 × 10^4^	1.02 × 10^4^	1.03 × 10^4^	9.47 × 10^3^	1.05 × 10^4^
F25	min	3.12 × 10^3^	3.93 × 10^3^	9.76 × 10^3^	6.10 × 10^3^	1.31 × 10^4^	1.18 × 10^4^	1.11 × 10^4^	4.91 × 10^3^	1.12 × 10^4^
	std	6.68 × 10^1^	6.88 × 10^2^	1.39 × 10^3^	3.31 × 10^2^	2.41 × 10^3^	1.99 × 10^3^	1.60 × 10^3^	1.05 × 10^3^	2.01 × 10^3^
	avg	3.22 × 10^3^	5.20 × 10^3^	1.43 × 10^4^	6.78 × 10^3^	1.68 × 10^4^	1.61 × 10^4^	1.49 × 10^4^	6.70 × 10^3^	1.41 × 10^4^
F26	min	3.47 × 10^3^	7.85 × 10^3^	1.73 × 10^4^	1.14 × 10^4^	1.68 × 10^4^	1.57 × 10^4^	1.62 × 10^4^	1.14 × 10^4^	1.46 × 10^4^
	std	3.02 × 10^3^	1.64 × 10^3^	4.48 × 10^2^	7.52 × 10^2^	1.38 × 10^3^	1.24 × 10^3^	7.77 × 10^2^	1.72 × 10^3^	1.69 × 10^3^
	avg	6.46 × 10^3^	1.21 × 10^4^	1.85 × 10^4^	1.31 × 10^4^	1.94 × 10^4^	1.83 × 10^4^	1.78 × 10^4^	1.48 × 10^4^	1.70 × 10^4^
F27	min	3.58 × 10^3^	3.86 × 10^3^	6.01 × 10^3^	4.99 × 10^3^	5.62 × 10^3^	4.89 × 10^3^	4.82 × 10^3^	3.87 × 10^3^	5.35 × 10^3^
	std	1.85 × 10^2^	2.64 × 10^2^	6.80 × 10^2^	4.57 × 10^2^	4.96 × 10^2^	5.46 × 10^2^	8.81 × 10^2^	4.74 × 10^2^	5.91 × 10^2^
	avg	3.88 × 10^3^	4.27 × 10^3^	7.39 × 10^3^	6.21 × 10^3^	6.61 × 10^3^	6.10 × 10^3^	6.30 × 10^3^	4.34 × 10^3^	6.45 × 10^3^
F28	min	3.41 × 10^3^	4.73 × 10^3^	1.20 × 10^4^	6.70 × 10^3^	1.05 × 10^4^	8.56 × 10^3^	1.08 × 10^4^	4.62 × 10^3^	9.17 × 10^3^
	std	1.20 × 10^2^	5.25 × 10^2^	1.25 × 10^3^	3.74 × 10^2^	1.28 × 10^3^	2.28 × 10^3^	1.58 × 10^3^	1.44 × 10^3^	1.36 × 10^3^
	avg	3.63 × 10^3^	5.65 × 10^3^	1.42 × 10^4^	7.27 × 10^3^	1.33 × 10^4^	1.35 × 10^4^	1.37 × 10^4^	6.72 × 10^3^	1.12 × 10^4^
F29	min	4.48 × 10^3^	5.59 × 10^3^	1.45 × 10^4^	7.76 × 10^3^	1.11 × 10^4^	7.34 × 10^3^	8.20 × 10^3^	6.45 × 10^3^	7.84 × 10^3^
	std	5.19 × 10^2^	8.08 × 10^2^	1.14 × 10^5^	1.54 × 10^3^	7.85 × 10^3^	1.13 × 10^5^	1.81 × 10^4^	1.65 × 10^3^	8.98 × 10^3^
	avg	5.59 × 10^3^	6.86 × 10^3^	1.17 × 10^5^	9.99 × 10^3^	2.01 × 10^4^	5.98 × 10^4^	2.74 × 10^4^	8.73 × 10^3^	1.84 × 10^4^
F30	min	6.30 × 10^7^	8.61 × 10^7^	3.45 × 10^9^	2.22 × 10^8^	2.34 × 10^9^	6.90 × 10^8^	7.27 × 10^8^	4.36 × 10^7^	6.11 × 10^8^
	std	3.02 × 10^7^	1.01 × 10^8^	2.22 × 10^9^	1.07 × 10^8^	7.86 × 10^8^	4.16 × 10^9^	3.47 × 10^9^	1.87 × 10^8^	1.48 × 10^9^
	avg	1.17 × 10^8^	2.11 × 10^8^	8.06 × 10^9^	4.13 × 10^8^	3.64 × 10^9^	6.04 × 10^9^	6.39 × 10^9^	3.22 × 10^8^	2.73 × 10^9^
Friedman Rank		1.07	2.45	7.79	3.03	7.62	6.41	6.97	3.48	6.17
Rank		1	2	9	3	8	6	7	4	5

**Table 14:** Test results for the dim = 100 CEC2017 test suite.

Func	Metrics	CMSCSO	SCSO	BBO	CAOA	FGO	MShOA	TGCOA	TOC	ZOA
F1	min	2.92 × 10^9^	8.41 × 10^10^	2.41 × 10^11^	1.31 × 10^11^	2.19 × 10^11^	2.29 × 10^11^	2.36 × 10^11^	1.05 × 10^11^	2.12 × 10^11^
	std	1.84 × 10^9^	1.46 × 10^10^	1.07 × 10^10^	5.53 × 10^9^	3.32 × 10^10^	1.40 × 10^10^	1.33 × 10^10^	2.81 × 10^10^	2.80 × 10^10^
	avg	6.21 × 10^9^	1.09 × 10^11^	2.62 × 10^11^	1.46 × 10^11^	2.93 × 10^11^	2.71 × 10^11^	2.66 × 10^11^	1.61 × 10^11^	2.71 × 10^11^
F3	min	2.98 × 10^5^	3.00 × 10^5^	3.16 × 10^5^	2.97 × 10^5^	6.62 × 10^5^	3.15 × 10^5^	5.13 × 10^5^	2.71 × 10^5^	5.45 × 10^5^
	std	1.04 × 10^5^	5.22 × 10^4^	2.60 × 10^5^	3.22 × 10^4^	1.05 × 10^8^	3.67 × 10^4^	1.07 × 10^5^	3.28 × 10^5^	2.00 × 10^5^
	avg	3.66 × 10^5^	3.36 × 10^5^	4.40 × 10^5^	3.53 × 10^5^	4.50 × 10^7^	3.68 × 10^5^	6.95 × 10^5^	6.05 × 10^5^	9.22 × 10^5^
F4	min	1.37 × 10^3^	7.69 × 10^3^	8.08 × 10^4^	2.77 × 10^4^	7.49 × 10^4^	6.01 × 10^4^	7.84 × 10^4^	1.77 × 10^4^	5.00 × 10^4^
	std	2.17 × 10^2^	4.22 × 10^3^	1.32 × 10^4^	2.68 × 10^3^	1.53 × 10^4^	2.33 × 10^4^	1.14 × 10^4^	1.05 × 10^4^	2.22 × 10^4^
	avg	1.72 × 10^3^	1.38 × 10^4^	1.13 × 10^5^	3.61 × 10^4^	9.89 × 10^4^	1.03 × 10^5^	1.04 × 10^5^	3.32 × 10^4^	8.38 × 10^4^
F5	min	1.31 × 10^3^	1.51 × 10^3^	2.06 × 10^3^	1.36 × 10^3^	2.05 × 10^3^	1.98 × 10^3^	2.03 × 10^3^	1.85 × 10^3^	2.09 × 10^3^
	std	5.26 × 10^1^	7.82 × 10^1^	3.30 × 10^1^	1.07 × 10^2^	1.03 × 10^2^	5.84 × 10^1^	5.21 × 10^1^	9.49 × 10^1^	8.66 × 10^1^
	avg	1.46 × 10^3^	1.63 × 10^3^	2.15 × 10^3^	1.58 × 10^3^	2.28 × 10^3^	2.10 × 10^3^	2.14 × 10^3^	2.05 × 10^3^	2.25 × 10^3^
F6	min	6.67 × 10^2^	6.80 × 10^2^	7.11 × 10^2^	6.65 × 10^2^	7.13 × 10^2^	6.97 × 10^2^	7.09 × 10^2^	6.88 × 10^2^	7.04 × 10^2^
	std	3.69 × 10^0^	3.64 × 10^0^	2.51 × 10^0^	6.18 × 10^0^	5.13 × 10^0^	6.27 × 10^0^	4.00 × 10^0^	6.55 × 10^0^	6.43 × 10^0^
	avg	6.73 × 10^2^	6.87 × 10^2^	7.16 × 10^2^	6.76 × 10^2^	7.21 × 10^2^	7.13 × 10^2^	7.17 × 10^2^	7.01 × 10^2^	7.18 × 10^2^
F7	min	2.70 × 10^3^	3.20 × 10^3^	3.90 × 10^3^	2.60 × 10^3^	5.10 × 10^3^	3.88 × 10^3^	3.68 × 10^3^	3.56 × 10^3^	4.26 × 10^3^
	std	2.10 × 10^2^	1.21 × 10^2^	5.79 × 10^1^	2.58 × 10^2^	5.29 × 10^2^	8.96 × 10^1^	1.19 × 10^2^	3.66 × 10^2^	5.08 × 10^2^
	avg	3.04 × 10^3^	3.45 × 10^3^	4.03 × 10^3^	2.96 × 10^3^	6.06 × 10^3^	4.08 × 10^3^	4.06 × 10^3^	4.01 × 10^3^	5.24 × 10^3^
F8	min	1.72 × 10^3^	1.87 × 10^3^	2.59 × 10^3^	1.77 × 10^3^	2.51 × 10^3^	2.33 × 10^3^	2.50 × 10^3^	2.22 × 10^3^	2.44 × 10^3^
	std	8.16 × 10^1^	9.20 × 10^1^	3.28 × 10^1^	1.02 × 10^2^	8.84 × 10^1^	9.28 × 10^1^	5.61 × 10^1^	1.17 × 10^2^	9.05 × 10^1^
	avg	1.93 × 10^3^	2.08 × 10^3^	2.65 × 10^3^	1.95 × 10^3^	2.68 × 10^3^	2.56 × 10^3^	2.63 × 10^3^	2.47 × 10^3^	2.67 × 10^3^
F9	min	2.92 × 10^4^	3.59 × 10^4^	7.84 × 10^4^	3.28 × 10^4^	9.72 × 10^4^	6.47 × 10^4^	6.87 × 10^4^	4.81 × 10^4^	6.96 × 10^4^
	std	3.42 × 10^3^	6.58 × 10^3^	3.73 × 10^3^	8.74 × 10^3^	1.09 × 10^4^	7.53 × 10^3^	8.32 × 10^3^	1.07 × 10^4^	1.27 × 10^4^
	avg	3.54 × 10^4^	4.78 × 10^4^	8.57 × 10^4^	4.79 × 10^4^	1.19 × 10^5^	8.41 × 10^4^	8.81 × 10^4^	6.75 × 10^4^	1.11 × 10^5^
F10	min	1.74 × 10^4^	1.96 × 10^4^	3.21 × 10^4^	2.16 × 10^4^	3.22 × 10^4^	2.94 × 10^4^	3.08 × 10^4^	2.74 × 10^4^	3.14 × 10^4^
	std	1.54 × 10^3^	1.93 × 10^3^	6.09 × 10^2^	2.40 × 10^3^	8.54 × 10^2^	1.24 × 10^3^	1.21 × 10^3^	1.40 × 10^3^	9.26 × 10^2^
	avg	1.98 × 10^4^	2.28 × 10^4^	3.35 × 10^4^	2.53 × 10^4^	3.45 × 10^4^	3.23 × 10^4^	3.32 × 10^4^	3.01 × 10^4^	3.37 × 10^4^
F11	min	7.68 × 10^4^	8.39 × 10^4^	2.06 × 10^5^	1.21 × 10^5^	2.60 × 10^5^	1.85 × 10^5^	1.70 × 10^5^	1.03 × 10^5^	2.92 × 10^5^
	std	1.80 × 10^4^	1.65 × 10^4^	2.69 × 10^4^	1.48 × 10^4^	9.74 × 10^4^	2.47 × 10^4^	7.32 × 10^4^	1.08 × 10^5^	1.16 × 10^5^
	avg	1.07 × 10^5^	1.25 × 10^5^	2.40 × 10^5^	1.45 × 10^5^	4.96 × 10^5^	2.26 × 10^5^	3.53 × 10^5^	2.16 × 10^5^	4.90 × 10^5^
F12	min	4.30 × 10^8^	1.37 × 10^10^	1.41 × 10^11^	6.74 × 10^10^	1.15 × 10^11^	1.43 × 10^11^	1.45 × 10^11^	2.16 × 10^10^	1.08 × 10^11^
	std	3.70 × 10^8^	1.31 × 10^10^	1.85 × 10^10^	6.54 × 10^9^	2.18 × 10^10^	2.95 × 10^10^	2.37 × 10^10^	1.61 × 10^10^	2.30 × 10^10^
	avg	1.12 × 10^9^	3.42 × 10^10^	2.00 × 10^11^	8.59 × 10^10^	1.58 × 10^11^	1.93 × 10^11^	1.97 × 10^11^	4.26 × 10^10^	1.44 × 10^11^
F13	min	2.93 × 10^4^	8.24 × 10^8^	3.14 × 10^10^	1.03 × 10^10^	2.38 × 10^10^	3.09 × 10^10^	2.30 × 10^10^	1.21 × 10^9^	1.87 × 10^10^
	std	4.63 × 10^4^	3.37 × 10^9^	4.45 × 10^9^	1.67 × 10^9^	7.45 × 10^9^	6.97 × 10^9^	7.48 × 10^9^	2.31 × 10^9^	6.21 × 10^9^
	avg	8.24 × 10^4^	5.94 × 10^9^	4.68 × 10^10^	1.43 × 10^10^	3.41 × 10^10^	4.52 × 10^10^	4.55 × 10^10^	4.40 × 10^9^	3.26 × 10^10^
F14	min	2.20 × 10^6^	4.33 × 10^6^	8.86 × 10^7^	2.29 × 10^6^	6.12 × 10^7^	2.64 × 10^7^	4.45 × 10^7^	5.52 × 10^6^	4.58 × 10^7^
	std	3.47 × 10^6^	6.07 × 10^6^	1.01 × 10^8^	2.47 × 10^6^	7.40 × 10^7^	7.81 × 10^7^	1.15 × 10^8^	1.65 × 10^7^	4.60 × 10^7^
	avg	7.28 × 10^6^	1.18 × 10^7^	2.27 × 10^8^	7.65 × 10^6^	2.00 × 10^8^	1.22 × 10^8^	1.73 × 10^8^	2.24 × 10^7^	1.12 × 10^8^
F15	min	2.04 × 10^4^	9.90 × 10^7^	1.94 × 10^10^	2.98 × 10^9^	9.09 × 10^9^	3.96 × 10^9^	8.97 × 10^9^	2.17 × 10^8^	5.12 × 10^9^
	std	2.92 × 10^4^	1.43 × 10^9^	3.59 × 10^9^	9.06 × 10^8^	3.54 × 10^9^	7.86 × 10^9^	6.39 × 10^9^	7.49 × 10^8^	4.19 × 10^9^
	avg	5.56 × 10^4^	1.50 × 10^9^	2.91 × 10^10^	4.62 × 10^9^	1.53 × 10^10^	2.36 × 10^10^	2.55 × 10^10^	1.12 × 10^9^	1.26 × 10^10^
F16	min	6.26 × 10^3^	8.16 × 10^3^	2.10 × 10^4^	1.14 × 10^4^	1.63 × 10^4^	1.42 × 10^4^	1.80 × 10^4^	1.08 × 10^4^	1.43 × 10^4^
	std	9.86 × 10^2^	1.31 × 10^3^	2.43 × 10^3^	1.31 × 10^3^	2.08 × 10^3^	4.35 × 10^3^	4.02 × 10^3^	2.37 × 10^3^	2.31 × 10^3^
	avg	8.14 × 10^3^	1.04 × 10^4^	2.61 × 10^4^	1.40 × 10^4^	2.00 × 10^4^	2.15 × 10^4^	2.56 × 10^4^	1.52 × 10^4^	1.85 × 10^4^
F17	min	4.86 × 10^3^	5.83 × 10^3^	2.44 × 10^6^	1.71 × 10^4^	1.47 × 10^5^	2.42 × 10^5^	2.15 × 10^5^	8.17 × 10^3^	2.51 × 10^4^
	std	6.65 × 10^2^	2.51 × 10^4^	1.04 × 10^7^	2.89 × 10^4^	3.28 × 10^6^	9.03 × 10^6^	1.09 × 10^7^	1.17 × 10^5^	1.74 × 10^6^
	avg	6.24 × 10^3^	1.82 × 10^4^	1.72 × 10^7^	5.43 × 10^4^	3.68 × 10^6^	6.52 × 10^6^	1.14 × 10^7^	3.78 × 10^4^	1.12 × 10^6^
F18	min	2.28 × 10^6^	3.03 × 10^6^	6.92 × 10^7^	2.82 × 10^6^	1.86 × 10^8^	4.16 × 10^7^	7.25 × 10^7^	3.99 × 10^6^	6.66 × 10^7^
	std	6.89 × 10^6^	4.69 × 10^6^	1.01 × 10^8^	1.90 × 10^6^	1.14 × 10^8^	2.38 × 10^8^	1.85 × 10^8^	2.76 × 10^7^	6.58 × 10^7^
	avg	8.50 × 10^6^	9.44 × 10^6^	2.73 × 10^8^	5.97 × 10^6^	3.71 × 10^8^	2.59 × 10^8^	3.14 × 10^8^	3.01 × 10^7^	1.90 × 10^8^
F19	min	2.35 × 10^5^	4.36 × 10^7^	1.94 × 10^10^	3.14 × 10^9^	9.66 × 10^9^	4.39 × 10^9^	1.47 × 10^10^	2.29 × 10^8^	6.07 × 10^9^
	std	2.35 × 10^6^	1.59 × 10^9^	2.99 × 10^9^	1.15 × 10^9^	3.33 × 10^9^	7.54 × 10^9^	4.66 × 10^9^	1.24 × 10^9^	5.09 × 10^9^
	avg	3.78 × 10^6^	1.52 × 10^9^	2.63 × 10^10^	4.94 × 10^9^	1.56 × 10^10^	2.18 × 10^10^	2.57 × 10^10^	1.39 × 10^9^	1.45 × 10^10^
F20	min	4.67 × 10^3^	5.22 × 10^3^	7.38 × 10^3^	3.89 × 10^3^	8.19 × 10^3^	6.99 × 10^3^	7.07 × 10^3^	5.86 × 10^3^	7.80 × 10^3^
	std	6.02 × 10^2^	5.34 × 10^2^	2.95 × 10^2^	6.97 × 10^2^	3.24 × 10^2^	4.37 × 10^2^	4.48 × 10^2^	6.21 × 10^2^	4.48 × 10^2^
	avg	5.63 × 10^3^	6.03 × 10^3^	8.31 × 10^3^	5.73 × 10^3^	8.82 × 10^3^	7.81 × 10^3^	8.17 × 10^3^	7.02 × 10^3^	8.64 × 10^3^
F21	min	1.15 × 10^4^	1.38 × 10^4^	2.04 × 10^4^	1.24 × 10^4^	1.77 × 10^4^	1.83 × 10^4^	1.95 × 10^4^	1.64 × 10^4^	1.82 × 10^4^
	std	1.01 × 10^3^	7.63 × 10^2^	5.49 × 10^2^	1.10 × 10^3^	9.86 × 10^2^	1.10 × 10^3^	1.05 × 10^3^	1.15 × 10^3^	1.02 × 10^3^
	avg	1.36 × 10^4^	1.51 × 10^4^	2.15 × 10^4^	1.46 × 10^4^	2.01 × 10^4^	2.08 × 10^4^	2.18 × 10^4^	1.88 × 10^4^	2.02 × 10^4^
F22	min	5.03 × 10^4^	5.63 × 10^4^	8.57 × 10^4^	6.09 × 10^4^	8.73 × 10^4^	8.44 × 10^4^	7.51 × 10^4^	6.94 × 10^4^	8.60 × 10^4^
	std	3.88 × 10^3^	4.35 × 10^3^	2.01 × 10^3^	6.43 × 10^3^	2.07 × 10^3^	2.39 × 10^3^	3.83 × 10^3^	3.47 × 10^3^	2.23 × 10^3^
	avg	5.76 × 10^4^	6.41 × 10^4^	9.10 × 10^4^	7.11 × 10^4^	9.40 × 10^4^	8.89 × 10^4^	8.73 × 10^4^	7.70 × 10^4^	9.08 × 10^4^
F23	min	1.19 × 10^4^	1.49 × 10^4^	2.76 × 10^4^	2.02 × 10^4^	2.32 × 10^4^	2.03 × 10^4^	2.25 × 10^4^	1.96 × 10^4^	2.11 × 10^4^
	std	1.42 × 10^3^	1.33 × 10^3^	1.28 × 10^3^	1.14 × 10^3^	1.19 × 10^3^	1.80 × 10^3^	1.88 × 10^3^	1.72 × 10^3^	1.74 × 10^3^
	avg	1.46 × 10^4^	1.76 × 10^4^	3.08 × 10^4^	2.25 × 10^4^	2.55 × 10^4^	2.51 × 10^4^	2.65 × 10^4^	2.30 × 10^4^	2.46 × 10^4^
F24	min	1.79 × 10^4^	1.90 × 10^4^	2.40 × 10^4^	1.86 × 10^4^	2.37 × 10^4^	2.33 × 10^4^	2.41 × 10^4^	2.24 × 10^4^	2.33 × 10^4^
	std	7.74 × 10^2^	7.95 × 10^2^	2.34 × 10^2^	6.53 × 10^2^	9.07 × 10^2^	5.97 × 10^2^	4.54 × 10^2^	1.14 × 10^3^	1.16 × 10^3^
	avg	1.95 × 10^4^	2.11 × 10^4^	2.47 × 10^4^	2.01 × 10^4^	2.62 × 10^4^	2.47 × 10^4^	2.49 × 10^4^	2.40 × 10^4^	2.55 × 10^4^
F25	min	3.90 × 10^3^	6.96 × 10^3^	2.73 × 10^4^	1.15 × 10^4^	2.64 × 10^4^	2.08 × 10^4^	2.42 × 10^4^	1.18 × 10^4^	2.28 × 10^4^
	std	1.85 × 10^2^	2.04 × 10^3^	1.30 × 10^3^	5.63 × 10^2^	6.51 × 10^3^	3.49 × 10^3^	2.48 × 10^3^	2.27 × 10^3^	5.30 × 10^3^
	avg	4.34 × 10^3^	1.08 × 10^4^	3.06 × 10^4^	1.29 × 10^4^	4.12 × 10^4^	2.93 × 10^4^	2.87 × 10^4^	1.73 × 10^4^	3.11 × 10^4^
F26	min	8.35 × 10^3^	2.15 × 10^4^	5.36 × 10^4^	3.47 × 10^4^	4.88 × 10^4^	4.55 × 10^4^	5.15 × 10^4^	3.11 × 10^4^	4.17 × 10^4^
	std	6.97 × 10^3^	3.95 × 10^3^	2.09 × 10^3^	1.76 × 10^3^	4.64 × 10^3^	4.63 × 10^3^	3.08 × 10^3^	7.11 × 10^3^	3.98 × 10^3^
	avg	2.09 × 10^4^	3.21 × 10^4^	5.80 × 10^4^	3.74 × 10^4^	5.70 × 10^4^	5.55 × 10^4^	5.67 × 10^4^	3.96 × 10^4^	4.97 × 10^4^
F27	min	3.98 × 10^3^	4.57 × 10^3^	1.24 × 10^4^	9.91 × 10^3^	9.60 × 10^3^	8.13 × 10^3^	9.49 × 10^3^	4.72 × 10^3^	8.74 × 10^3^
	std	3.02 × 10^2^	6.26 × 10^2^	1.36 × 10^3^	9.51 × 10^2^	9.86 × 10^2^	1.74 × 10^3^	1.35 × 10^3^	6.67 × 10^2^	1.48 × 10^3^
	avg	4.50 × 10^3^	5.54 × 10^3^	1.57 × 10^4^	1.14 × 10^4^	1.19 × 10^4^	1.13 × 10^4^	1.22 × 10^4^	5.76 × 10^3^	1.13 × 10^4^
F28	min	4.25 × 10^3^	1.12 × 10^4^	3.52 × 10^4^	1.85 × 10^4^	3.32 × 10^4^	2.99 × 10^4^	3.18 × 10^4^	1.34 × 10^4^	2.50 × 10^4^
	std	3.81 × 10^2^	1.74 × 10^3^	1.66 × 10^3^	6.62 × 10^2^	3.86 × 10^3^	3.40 × 10^3^	2.25 × 10^3^	2.70 × 10^3^	4.16 × 10^3^
	avg	4.83 × 10^3^	1.38 × 10^4^	3.84 × 10^4^	1.98 × 10^4^	4.09 × 10^4^	3.58 × 10^4^	3.56 × 10^4^	2.04 × 10^4^	3.43 × 10^4^
F29	min	8.79 × 10^3^	1.09 × 10^4^	1.53 × 10^5^	1.65 × 10^4^	5.12 × 10^4^	3.73 × 10^4^	6.35 × 10^4^	1.46 × 10^4^	3.35 × 10^4^
	std	9.57 × 10^2^	3.10 × 10^3^	4.26 × 10^5^	3.99 × 10^3^	2.65 × 10^5^	6.47 × 10^5^	4.50 × 10^5^	7.63 × 10^3^	1.57 × 10^5^
	avg	1.05 × 10^4^	1.54 × 10^4^	8.09 × 10^5^	2.41 × 10^4^	3.76 × 10^5^	5.87 × 10^5^	6.87 × 10^5^	2.23 × 10^4^	1.69 × 10^5^
F30	min	8.78 × 10^7^	2.24 × 10^8^	3.57 × 10^10^	1.05 × 10^10^	1.59 × 10^10^	2.33 × 10^10^	2.12 × 10^10^	1.29 × 10^9^	1.49 × 10^10^
	std	9.18 × 10^7^	3.45 × 10^9^	4.30 × 10^9^	2.09 × 10^9^	5.18 × 10^9^	8.98 × 10^9^	9.05 × 10^9^	1.05 × 10^9^	7.73 × 10^9^
	avg	2.32 × 10^8^	4.86 × 10^9^	4.37 × 10^10^	1.43 × 10^10^	2.71 × 10^10^	3.75 × 10^10^	4.16 × 10^10^	2.97 × 10^9^	2.76 × 10^10^
Friedman Rank		1.14	2.41	7.62	2.97	7.69	6.10	7.10	3.62	6.34
Rank		1	2	8	3	9	5	7	4	66

**Table 15:** Comparison of test results for the three-bar truss problem (the optimal results are shown in bold).

Algorithm	*x*_1_	*x*_2_	*f*_min_	*f*_avg_	std
CMSCSO	0.7887	0.4082	**263.8958**	**263.8959**	**3.42 × 10^−5^**
SCSO	0.7887	0.4081	263.8962	263.8991	0.0045
BBO	0.7808	0.4311	263.9442	265.0993	0.8359
CAOA	0.7878	0.4107	263.9018	263.9806	0.0645
FGO	0.7874	0.4135	264.0479	265.9784	1.7157
MShOA	0.7856	0.4169	263.9029	264.1430	0.1947
TGCOA	0.7861	0.4155	263.9006	264.2864	0.3807
TOC	0.7881	0.4098	263.8961	263.9151	0.0328
ZOA	0.7843	0.4207	263.9147	264.6625	0.5974

**Table 16:** Comparison of test results for the ten-bar truss problem (the optimal results are shown in bold).

Algorithm	*x*_1_	*x*_2_	*x*_3_	*x*_4_	*x*_5_	*x*_6_	*x*_7_	*x*_8_	*x*_9_	*x*_10_	*f*_min_	*f*_avg_	std
CMSCSO	0.0034	0.0016	0.0037	0.0016	0.0001	0.0004	0.0024	0.0022	0.0011	0.0014	**526.4586**	**532.0657**	**3.9242**
SCSO	0.0038	0.0013	0.0034	0.0017	0.0001	0.0005	0.0020	0.0028	0.0014	0.0010	528.6700	535.4371	4.9830
BBO	0.0026	0.0018	0.0035	0.0019	0.0002	0.0018	0.0027	0.0032	0.0018	0.0035	695.0843	822.4402	55.4846
CAOA	0.0026	0.0012	0.0039	0.0021	0.0009	0.0004	0.0027	0.0021	0.0020	0.0021	599.5651	630.3708	16.6458
FGO	0.0047	0.0018	0.0041	0.0023	0.0023	0.0006	0.0014	0.0022	0.0022	0.0014	660.1505	700.0796	26.1880
MShOA	0.0036	0.0019	0.0036	0.0019	0.0002	0.0005	0.0035	0.0018	0.0007	0.0014	564.7215	611.2504	30.9666
TGCOA	0.0039	0.0008	0.0025	0.0023	0.0010	0.0005	0.0023	0.0035	0.0028	0.0005	603.2874	637.3016	17.0242
TOC	0.0045	0.0009	0.0029	0.0018	0.0001	0.0006	0.0018	0.0032	0.0015	0.0009	540.3194	562.6218	26.1734
ZOA	0.0034	0.0022	0.0032	0.0016	0.0011	0.0007	0.0022	0.0034	0.0010	0.0023	625.5134	662.5328	33.8602

**Table 17:** Comparison of test results for the speed reducer problem (the optimal results are shown in bold).

Algorithm	*x*_1_	*x*_2_	*x*_3_	*x*_4_	*x*_5_	*x*_6_	*x*_7_	*f*_min_	*f*_avg_	std
CMSCSO	3.5000	0.7000	17	7.3902	7.8821	3.3507	5.2867	**2998.9595**	**3005.6112**	**3.5180**
SCSO	3.5008	0.7000	17	7.3028	7.8483	3.3607	5.2886	3001.4599	3008.0283	7.0349
BBO	3.5037	0.7000	27	8.0780	8.0780	3.7957	5.3529	5429.8540	5460.6476	25.4076
CAOA	3.5099	0.7000	17	7.7523	7.9168	3.3677	5.3046	3098.0135	3177.3153	59.8154
FGO	3.5364	0.7068	24	7.8650	7.8717	3.5704	5.3780	4652.0117	5.41 × 10^12^	1.06 × 10^13^
MShOA	3.5758	0.7032	17	7.4084	7.9750	3.3638	5.2868	3086.0228	3454.3703	467.7416
TGCOA	3.5347	0.7034	17	8.1226	8.0081	3.4015	5.3434	3087.0526	3470.5069	398.3089
TOC	3.5000	0.7000	17	7.3000	8.3000	3.3505	5.2869	3007.3900	3078.9839	63.1213
ZOA	3.5883	0.7079	18	7.7083	7.8417	3.4945	5.2902	3265.9664	4.55 × 10^11^	9.59 × 10^11^

**Table 18:** Comparison of test results for the reinforced concrete beam design problem (the optimal results are shown in bold).

Algorithm	*x*_1_	*x*_2_	*x*_3_	*f*_min_	*f*_avg_	std
CMSCSO	8.2086	30.0352	7.5000	**158.8050**	**158.8160**	0.0255
SCSO	8.2088	30.0356	7.5003	158.8110	158.8214	**0.0173**
BBO	8.2951	29.2765	8.4714	171.4578	182.6696	4.3313
CAOA	8.2125	30.3241	7.5003	158.8217	161.0306	3.5061
FGO	8.3335	29.7710	7.5931	160.8424	170.2947	5.1196
MShOA	8.2692	29.9540	7.5006	158.9912	159.7139	0.8503
TGCOA	8.2161	29.5144	7.5000	158.8266	159.2440	0.3502
TOC	8.2086	29.6327	7.5000	**158.8050**	158.9388	0.2237
ZOA	8.2363	30.3672	7.5034	158.9461	165.9697	5.2964

**Table 19:** Comparison of test results for the tubular column design problem (the optimal results are shown in bold).

Algorithm	x1	x2	*f*_min_	*f*_avg_	std
CMSCSO	6.54744	0.20870	**26.4860**	**26.4860**	**7.72** × 10**^−6^**
SCSO	6.54737	0.20872	26.4868	26.4882	0.0012
BBO	6.60565	0.20702	26.6130	27.0405	0.1984
CAOA	6.55147	0.20862	26.4974	26.5389	0.0272
FGO	6.45345	0.22682	27.2520	28.6121	0.8772
MShOA	7.00000	0.20848	26.5022	26.6629	0.1456
TGCOA	6.54678	0.20876	26.4874	26.6415	0.2164
TOC	6.54744	0.20870	**26.4860**	26.5999	0.2401
ZOA	6.52157	0.21136	26.5513	27.1959	0.5882

**Table 20:** Comparison of test results for the compression spring design problem (the optimal results are shown in bold).

Algorithm	*x*_1_	*x*_2_	*x*_3_	*f*_min_	*f*_avg_	std
CMSCSO	0.0500	0.3174	14.0280	**0.0127**	**0.0132**	0.0005
SCSO	0.0525	0.3755	10.2735	**0.0127**	0.0134	0.0007
BBO	0.0522	0.3648	11.2160	0.0132	**0.0132**	**0.0000**
CAOA	0.0524	0.3735	10.5264	0.0128	0.0141	0.0007
FGO	0.0665	0.6305	6.1569	0.0227	2.69 × 10^12^	8.51 × 10^12^
MShOA	0.0519	0.3625	11.1787	0.0129	0.0153	0.0020
TGCOA	0.0570	0.4994	6.1885	0.0133	0.0192	0.0064
TOC	0.0516	0.3541	11.4419	**0.0127**	**0.0132**	0.0012
ZOA	0.0541	0.3873	12.2073	0.0161	0.0292	0.0108

**Table 21:** Results of 2D WSN coverage optimization.

Algorithm	Coverage Rate
CMSCSO	96.30%
SCSO	89.20%
BBO	73.52%
CAOA	94.97%
FGO	79.29%
MShOA	82.10%
TGCOA	84.02%
TOC	90.53%
ZOA	80.03%

**Table 22:** Results of 3D WSN coverage optimization.

Algorithm	Coverage Rate
CMSCSO	89.54%
SCSO	85.88%
BBO	75.14%
CAOA	80.77%
FGO	74.98%
MShOA	78.24%
TGCOA	78.60%
TOC	82.62%
ZOA	74.84%

**Table 23:** Variables and constraints for the photovoltaic cell parameter identification problem.

Model	Parameters	*lb*	*ub*
SDM	*I_ph_*	0	1
	*I*_0_	0	1 × 10^−6^
	*R_s_*	0	0.5
	*R_sh_*	0	100
	*n*	1	2
DDM	*I_ph_*	0	1
	*I_0_*_1_	0	1 × 10^−6^
	*R_s_*	0	0.5
	*R_sh_*	0	100
	*I_0_*_2_	0	1 × 10^−6^
	*n*_1_	1	2
	*n*_2_	1	2

**Table 24:** Parameter identification results of SDM (the optimal results are shown in bold).

Algorithm	*I_ph_*	*I*_0_	*Rs*	*R_sh_*	*n*	*f*_min_	*f*_avg_	std
CMSCSO	0.76238	2.04 × 10^−7^	0.03790	34.25195	1.43663	**8.30 × 10^−4^**	**2.30 × 10^−3^**	**1.40 × 10^−3^**
SCSO	0.76622	9.98 × 10^−7^	0.01448	12.06178	1.61139	1.20 × 10^−3^	5.00 × 10^−3^	5.70 × 10^−3^
BBO	0.74940	1.96 × 10^−7^	0.18015	62.11395	1.55901	3.78 × 10^−2^	1.11 × 10^−1^	3.33 × 10^−2^
CAOA	0.75701	1.00 × 10^−6^	0.03880	28.79147	1.60855	1.15 × 10^−2^	1.37 × 10^−2^	2.00 × 10^−3^
FGO	0.69480	8.43 × 10^−7^	0.01422	15.99037	1.59888	2.47 × 10^−2^	6.23 × 10^−2^	1.95 × 10^−2^
MShOA	0.74462	5.16 × 10^−7^	0.04568	46.80310	1.53765	6.30 × 10^−3^	1.97 × 10^−2^	1.23 × 10^−2^
TGCOA	0.76719	1.02 × 10^−7^	0.04233	45.66122	1.37230	2.00 × 10^−3^	3.65 × 10^−2^	4.24 × 10^−2^
TOC	0.75951	4.65 × 10^−7^	0.03512	100.00000	1.51843	1.30 × 10^−3^	1.71 × 10^−2^	1.56 × 10^−2^
ZOA	0.76917	3.67 × 10^−7^	0.16365	76.45727	1.59290	2.15 × 10^−2^	4.99 × 10^−2^	2.37 × 10^−2^

**Table 25:** Parameter identification results of DDM (the optimal results are shown in bold).

Algorithm	*I_ph_*	*I*_01_	*Rs*	*R_sh_*	*n*_1_	*I*_02_	*N*_2_	*f*_min_	*f*_avg_	std
CMSCSO	0.76461	7.47 × 10^−^^7^	0.03786	45.66474	1.59754	7.50 × 10^−^^7^	1.92227	**1.10** **× 10^−3^**	**3.10 × 10^−3^**	**1.20 × 10^−3^**
SCSO	0.77172	9.92 × 10^−7^	0.03513	14.96124	1.86599	9.49 × 10^−^^7^	1.65134	1.60 × 10^−3^	4.80 × 10^−3^	3.20 × 10^−3^
BBO	0.74213	7.03 × 10^−^^7^	0.17089	28.74598	1.91260	5.14 × 10^−^^7^	1.84156	6.05 × 10^−2^	1.03 × 10^−1^	1.99 × 10^−2^
CAOA	0.76700	1.00 × 10^− 7^	0.04047	64.28091	1.62129	1.00 × 10^−^^6^	1.60120	5.60 × 10^−3^	1.16 × 10^−2^	3.80 × 10^−3^
FGO	0.76958	8.92 × 10^−^^8^	0.10807	48.00546	1.56475	5.43 × 10^−^^7^	1.68113	2.44 × 10^−2^	5.01 × 10^−2^	1.59 × 10^−2^
MShOA	0.76231	3.48 × 10^−^^7^	0.02896	28.62574	1.77175	8.74 × 10^−^^7^	1.61807	9.70 × 10^−3^	1.89 × 10^−2^	7.60 × 10^−3^
TGCOA	0.82236	6.69 × 10^−^^7^	0.17350	35.14014	1.83664	8.80 × 10^−^^7^	1.91287	1.80 × 10^−3^	1.86 × 10^−2^	2.07 × 10^−2^
TOC	0.76952	4.83 × 10^−^^7^	0.05548	31.42264	1.69094	4.67 × 10^−^^7^	1.62804	3.00 × 10^−3^	1.30 × 10^−2^	1.22 × 10^−2^
ZOA	0.75631	1.67 × 10^−^^7^	0.10297	70.24333	1.53014	8.59 × 10^−^^7^	1.87876	1.04 × 10^−2^	2.64 × 10^−2^	1.64 × 10^−2^

**Table 26:** Relative percentage improvements of CMSCSO over competing algorithms in engineering applications. (When a comparison algorithm generated infeasible solutions during repeated runs and a valid comparable mean objective value could not be obtained, the corresponding percentage was marked as “N/A”).

Problem	vs. SCSO	vs. BBO	vs. CAOA	vs. FGO	vs. MShOA	vs. TGCOA	vs. TOC	vs. ZOA
Three-Bar Trus	0.0012%	0.4539%	0.0321%	0.7830%	0.0935%	0.1478%	0.0073%	0.2897%
Ten-Bar Truss Design	0.6297%	35.3065%	15.5948%	23.9993%	12.9545%	16.5127%	5.4310%	19.6922%
Weight Min of a Speed Reducer	0.0804%	44.9587%	5.4041%	N/A	12.9911%	13.3956%	2.3830%	N/A
Reinforced Concrete Beam	0.0034%	13.0583%	1.3753%	6.7405%	0.5622%	0.2688%	0.0773%	4.3102%
Tubular Column	0.0083%	2.0506%	0.1993%	7.4308%	0.6635%	0.5837%	0.4282%	2.6103%
Compression Spring	1.4925%	0.0000%	6.3830%	N/A	13.7255%	31.2500%	0.0000%	54.7945%
2D WSN Coverage	7.9596%	30.9848%	1.4004%	21.4529%	17.2960%	14.6156%	6.3736%	20.3299%
3D WSN Coverage	4.2618%	19.1642%	10.8580%	19.4185%	14.4427%	13.9186%	8.3757%	19.6419%
SDM Identification	54.0000%	97.9279%	83.2117%	96.3082%	88.3249%	93.6986%	86.5497%	95.3908%
DDM Identification	35.4167%	96.9903%	73.2759%	93.8124%	83.5979%	83.3333%	76.1538%	88.2576%

## Data Availability

Data are contained within the article.

## Abbreviations

SCSO	Sand Cat Swarm Optimization
CMSCSO	Collaborative Multi-Strategy Sand Cat Swarm Optimization
GA	Genetic Algorithm
DE	Differential Evolution
ICA	Imperialist Competitive Algorithm
GSA	Gravitational Search Algorithm
BHO	Black Hole Algorithm
AEFA	Artificial Electric Field Algorithm
GWO	Grey Wolf Optimizer
HHO	Harris Hawks Optimization
SSA	Sparrow Search Algorithm
BBO	Beaver Behavior Optimizer
CAOA	Crocodile Ambush Optimization Algorithm
FGO	Fungal Growth Optimizer
MShOA	Mantis Shrimp Optimization Algorithm
TGCOA	Tetragonula Carbonaria Optimization Algorithm
TOC	Tornado Optimizer with Coriolis Force
ZOA	Zahhak Optimization Algorithm
WSNs	Wireless sensor networks
std	Standard deviation
SDM	Single-diode model
DDM	Double-diode model
